# Supplementary material for: Effectiveness of Implementing Hospital Wastewater Treatment Systems as a Measure to Mitigate the Microbial and Antimicrobial Burden on the Environment
Source: Antibiotics (Basel). 2025 Aug 7;14(8):807. doi: 10.3390/antibiotics14080807 (PMC12382850; doi:10.3390/antibiotics14080807)
Supplement: Supplementary file 1 [file antibiotics-14-00807-s001.zip › Table-S1 All raw read sequence files are available from the DRA SRA database.pdf]

Table S1. All raw read sequence files are available from the DRA/SRA database

Run Files	BioProject	BioSample	Experiment	Run
TOHO-20240907-Influent	PRJDB20415	SAMD00889682	DRX637899	DRR657618
TOHO-20240908-Influent	PRJDB20415	SAMD00889683	DRX637900	DRR657619
TOHO-20240908-Ozone	PRJDB20415	SAMD00889684	DRX637901	DRR657620
TOHO-20240908-OzoneUV	PRJDB20415	SAMD00889685	DRX637902	DRR657621
TOHO-20240909-Influent	PRJDB20415	SAMD00889686	DRX637903	DRR657622
TOHO-20240909-Ozone	PRJDB20415	SAMD00889687	DRX637904	DRR657623
TOHO-20240909-OzoneUV	PRJDB20415	SAMD00889688	DRX637905	DRR657624
TOHO-20240910-Influent	PRJDB20415	SAMD00889689	DRX637906	DRR657625
TOHO-20240910-Ozone	PRJDB20415	SAMD00889690	DRX637907	DRR657626
TOHO-20240910-OzoneUV	PRJDB20415	SAMD00889691	DRX637908	DRR657627
TOHO-20240911-Influent	PRJDB20415	SAMD00889692	DRX637909	DRR657628
TOHO-20240911-Ozone	PRJDB20415	SAMD00889693	DRX637910	DRR657629
TOHO-20240911-OzoneUV	PRJDB20415	SAMD00889694	DRX637911	DRR657630
TOHO-20240912-Influent	PRJDB20415	SAMD00889695	DRX637912	DRR657631
TOHO-20240912-Ozone	PRJDB20415	SAMD00889696	DRX637913	DRR657632
TOHO-20240912-OzoneUV	PRJDB20415	SAMD00889697	DRX637914	DRR657633
