# Supplementary material for: Effectiveness of Implementing Hospital Wastewater Treatment Systems as a Measure to Mitigate the Microbial and Antimicrobial Burden on the Environment
Source: Antibiotics (Basel). 2025 Aug 7;14(8):807. doi: 10.3390/antibiotics14080807 (PMC12382850; doi:10.3390/antibiotics14080807)
Supplement: Supplementary file 1 [file antibiotics-14-00807-s001.zip › Table-S2.pdf]

Table S2. Classification and LC-MS/MS parameters of each antimicrobials.

Classification	Compound	Ionization mode	Precursor ion (*m/z*)	Product ion (*m/z*)	Cone voltage (V)	Collision energy (eV)
β-lactams	Ampicillin	ESI+	350.2	*105.9*, 192.0	29	24
	Benzylpenicillin	ESI+	335.2	*160.1*, 174.0	32	23
	Cefdinir	ESI+	369.2	170.0, *227.0*	30	20
	Cefpodoxime	ESI+	427.8	*240.5*, 395.9	31	15
	Cefpodoxime proxetil	ESI+	557.5	*409.8*, 525.2	30	18
	Ceftiofur	ESI+	524.1	*240.9*	38	18
New quinolones	Ciprofloxacin	ESI+	332.2	*288.2*, 314.2	40	25
	Enrofloxacin	ESI+	360.0	*316.2*, 245.2	37	19
	Levofloxacin	ESI+	362.2	261.2, *318.2*	40	21
Macrolides	Azithromycin	ESI+	350.2	*105.9*, 192.0	29	24
	Clarithromycin	ESI+	748.2	316.6, *558.3*	38	18
Tetracyclines	Chlortetracycline	ESI+	479.2	443.5, *461.5*	36	20
	Doxycycline	ESI+	445.2	*428.3*	32	18
	Minocycline	ESI+	458.3	*441.0*	36	21
	Oxytetracycline	ESI+	461.2	*425.8*	28	19
	Tetracycline	ESI+	445.2	*409.9*, 427.1	28	20
Glycopeptide	Vancomycin	ESI+	724.2	82.9, *100.2*	17	18

Product ions in italics were used for quantification.
