# Supplementary material for: Effectiveness of Implementing Hospital Wastewater Treatment Systems as a Measure to Mitigate the Microbial and Antimicrobial Burden on the Environment
Source: Antibiotics (Basel). 2025 Aug 7;14(8):807. doi: 10.3390/antibiotics14080807 (PMC12382850; doi:10.3390/antibiotics14080807)
Supplement: Supplementary file 1 [file antibiotics-14-00807-s001.zip › Table-S3.pdf]

Table S3. Validation of the method characteristics for analysis of antimicrobials in wastewater.

Classification	Antimicrobials	Recovery (%) (SD, n=3)	LOD (ng/L)	LOQ (ng/L)	Calibration range (ng/mL)	Correlation coefficient (r^2)
β-lactams	Ampicillin	101 (1)	1.6	5.4	0.5–200	0.99
	Benzylpenicillin	80 (8)	0.5	1.7	0.5–200	0.99
	Cefdinir	111 (3)	0.2	0.8	0.5–200	0.99
	Cefpodoxime	97 (6)	0.3	1.1	0.5–200	0.99
New quinolones	Ciprofloxacin	58 (8)	0.7	2.2	0.5–200	0.99
	Levofloxacin	61 (19)	0.3	1.0	0.5–200	0.99
Macrolides	Azithromycin	104 (15)	0.3	1.1	0.5–200	0.99
	Clarithromycin	120 (24)	0.5	1.6	0.5–200	0.99
Tetracyclines	Doxycycline	95 (7)	0.3	1.0	0.5–200	0.99
	Minocycline	122 (5)	0.3	1.0	0.5–200	0.99
Glycopeptide	Vancomycin	86 (5)	0.6	1.9	0.5–200	0.99
