# Supplementary material for: Effectiveness of Implementing Hospital Wastewater Treatment Systems as a Measure to Mitigate the Microbial and Antimicrobial Burden on the Environment
Source: Antibiotics (Basel). 2025 Aug 7;14(8):807. doi: 10.3390/antibiotics14080807 (PMC12382850; doi:10.3390/antibiotics14080807)
Supplement: Supplementary file 1 [file antibiotics-14-00807-s001.zip › Table-S4_mDNAseq-CZID.pdf]

Table S4. Detected counts of sequencing reads for each bacteria genus by metagenomic DNA-Seq analysis

Sampling date	2024-0907	2024-0908			2024-0909			2024-0910			2024-0911			2024-0912		
Taxon Name	TOHO-20240907-Influent_S1	TOHO-20240908-Influent_S2	TOHO-20240908-Ozone_S3	TOHO-20240908-OzoneUV_S4	TOHO-20240909-Influent_S5	TOHO-20240909-Ozone_S6	TOHO-20240909-OzoneUV_S7	TOHO-20240910-Influent_S8	TOHO-20240910-Ozone_S9	TOHO-20240910-OzoneUV_S10	TOHO-20240911-Influent_S11	TOHO-20240911-Ozone_S12	TOHO-20240911-OzoneUV_S13	TOHO-20240912-Influent_S14	TOHO-20240912-Ozone_S15	TOHO-20240912-OzoneUV_S16
DNA conc. (ng/µl)	15.7	18.1	0.6	0.5	17	0.5	1.3	14.7	1.5	0.9	22.4	0.9	1	16.7	0.5	1
reads_after_fastp	9,231,822	6,822,736	6,611,630	7,230,522	#######	7,128,558	220,404	264,340	957,714	196,118	333,654	287,218	318,280	669,062	346,224	4,788
CZ-ID assigned reads	762,019	899,739	891,885	1,007,287	968,443	906,455	88,283	104,902	439,776	88,235	147,609	108,939	150,622	371,072	200,585	2,786
Arcobacter butzleri	42,345	89,507	84,252	124,369	126,026	104,436	3,258	4,823	39,015	4,328	8,941	3,579	5,857	12,166	4,706	32
Sulfurospirillum sp. UCH001	28,250	111,811	96,011	61,836	41,090	63,034	12,349	9,375	24,333	2,680	8,014	12,674	10,417	11,721	2,138	40
Citrobacter freundii	75,375	42,617	22,322	61,409	123,340	73,744	4,640	2,716	23,390	7,604	8,881	6,041	2,938	11,801	7,088	64
Tolumonas auensis	43,598	55,574	43,203	48,316	54,015	49,176	2,357	2,162	12,889	1,789	2,895	2,671	2,356	4,685	1,530	9
Cloacibacterium normanense	28,043	46,982	60,347	41,531	25,347	18,597	2,730	2,604	12,265	921	1,517	2,258	2,433	3,730	790	6
Arcobacter cryaerophilus	21,253	46,661	53,222	47,989	28,734	24,144	1,923	2,670	10,317	1,056	1,884	2,154	2,345	3,232	826	6
Aeromonas caviae	24,082	22,515	24,248	33,539	28,973	23,257	2,981	3,493	17,638	3,029	3,572	3,785	3,508	9,695	2,935	31
Phocaeicola dorei	22,565	14,135	20,408	28,187	23,966	24,993	1,971	3,114	14,763	3,028	4,304	2,444	3,395	5,657	2,031	20
Phocaeicola vulgatus	21,706	12,310	18,524	24,819	18,803	20,942	1,685	2,709	12,142	2,264	3,271	2,039	2,846	4,872	1,524	5
Acinetobacter tandoii	10,086	35,027	8,109	27,823	12,378	21,742	3,427	863	12,167	1,044	3,361	4,782	741	6,420	1,696	20
Prevotella copri	15,803	9,212	16,857	18,933	14,914	23,506	1,506	3,067	10,350	2,000	4,025	1,741	3,302	4,121	1,592	16
Pseudomonas putida	285	339	1,921	3,267	1,925	1,436	83	667	3,089	2,685	3,549	145	18,965	68,067	20,708	239
uncultured bacterium	14,473	14,179	12,534	13,372	13,514	15,120	1,285	1,808	7,359	1,368	2,238	1,604	2,136	3,436	1,238	16
Faecalibacterium prausnitzii	13,762	8,093	12,372	15,665	12,821	17,681	797	1,241	6,817	1,096	1,831	990	1,697	2,781	842	6
Escherichia coli	15,398	10,853	11,494	12,424	11,854	12,298	1,414	2,250	5,891	1,511	2,382	1,565	2,501	3,580	1,406	18
Arcobacter cibarius	9,704	25,042	12,622	16,005	11,611	9,532	967	692	5,105	436	844	780	551	1,543	349	4
Parabacteroides distasonis	11,367	9,569	10,541	13,411	12,783	11,312	1,416	1,771	7,989	1,552	1,844	1,540	1,704	2,965	1,170	7
Bacteroides ovatus	12,448	6,570	9,256	11,322	11,220	10,483	1,039	1,427	6,129	1,427	1,710	1,188	1,497	2,025	945	5
Bacteroides uniformis	9,582	5,601	8,663	12,590	9,650	10,912	947	1,768	7,436	1,458	2,161	1,325	1,880	3,119	1,037	4
Klebsiella pneumoniae	10,148	7,760	9,801	8,102	9,919	8,782	1,314	1,855	5,271	1,598	1,814	1,542	2,240	2,871	1,536	13
Bacteroides fragilis	10,327	7,344	7,985	8,829	7,305	10,439	847	1,260	5,000	1,047	1,628	1,117	1,514	2,071	708	5
Bacteroides thetaiotaomicron	8,092	5,367	8,293	8,222	7,844	10,178	898	1,324	4,576	1,082	1,558	952	1,396	2,070	813	3
Aeromonas hydrophila	4,874	5,448	5,996	9,339	9,523	9,624	599	837	4,545	755	1,586	694	720	2,092	851	10
Citrobacter portucalensis	8,052	9,039	979	7,205	7,641	9,072	564	462	2,704	661	902	866	482	1,201	508	0
Pseudomonas monteilii	41	88	415	850	1,389	1,495	10	105	722	1,223	3,902	21	2,948	18,520	14,889	278
Pseudomonas aeruginosa	2,972	3,374	3,729	3,578	4,019	3,356	535	813	2,382	881	1,865	985	2,628	7,493	5,691	109
Acidovorax sp. 1608163	3,756	5,801	7,032	6,126	5,351	3,841	650	828	2,522	452	619	826	890	1,301	540	4
Comamonas aquatica	7,372	6,692	2,798	9,401	2,950	2,092	664	319	3,547	322	271	848	362	1,760	295	7
Acinetobacter wuhouensis	1,838	4,910	3,876	2,459	2,497	1,761	1,074	875	1,524	357	503	1,431	1,514	8,291	3,344	43
uncultured organism	4,605	2,889	4,179	4,934	4,999	4,943	400	689	3,027	660	782	468	780	1,238	472	8
Aeromonas media	1,528	5,411	4,864	5,503	3,755	4,027	367	530	1,983	310	498	463	523	991	271	2
Laribacter hongkongensis	5,294	4,349	4,305	3,963	5,650	3,404	283	307	1,005	167	395	381	292	583	208	0
Megamonas funiformis	4,484	2,725	4,723	5,159	3,267	4,489	309	486	2,166	375	561	243	493	789	256	2
Pseudomonas oryzae	135	350	1,029	733	805	1,043	58	112	453	763	3,809	237	2,703	8,502	9,410	202
Acinetobacter sp. ACNIH2	436	1,498	478	1,487	965	1,558	137	55	674	92	586	217	106	4,723	15,352	92
Bacteroides xylanisolvens	3,523	2,678	3,291	3,935	3,996	3,562	316	471	2,112	509	641	386	586	799	307	0
Aeromonas veronii	2,086	2,923	3,644	3,581	3,573	4,712	298	367	1,830	431	804	384	401	940	295	5
Arcobacter ellisii	471	1,698	4,884	10,342	1,673	3,165	107	49	3,095	42	91	100	140	347	51	0
Desulfovibrio desulfuricans	3,898	2,913	3,610	2,984	5,089	4,089	293	296	891	193	487	329	311	502	173	2
[Eubacterium] rectale	3,730	1,724	2,687	3,840	3,860	3,699	162	360	2,216	468	600	182	483	1,145	369	4
Alicycliphilus denitrificans	1,992	2,547	3,014	3,091	2,527	2,005	421	810	2,079	469	642	733	675	1,400	2,723	7
unidentified plasmid	4,829	1,224	3,996	3,296	4,531	2,921	246	406	1,147	344	447	294	449	721	204	2
Acidovorax sp. KKS102	2,094	2,817	3,084	3,103	2,728	2,181	340	502	1,569	299	409	910	1,765	1,768	924	4
Acinetobacter baumannii	1,500	2,501	1,771	2,943	1,777	2,563	378	385	1,640	202	460	585	485	4,028	3,196	32
Acinetobacter cumulans	1,636	3,307	8,343	2,150	929	484	747	1,456	1,545	150	114	689	845	644	134	2
Bacteroides caccae	3,417	2,547	2,463	3,133	2,981	2,956	337	363	1,582	398	467	402	417	636	282	4
Megamonas hypermegale	3,131	1,710	3,941	3,588	2,702	2,811	184	453	1,586	326	336	166	379	573	206	2
Pseudomonas alcaligenes	776	1,284	1,434	1,568	1,640	1,672	220	319	971	376	691	337	835	3,829	4,659	83
Anaerostipes hadrus	2,793	1,526	1,989	2,743	2,636	3,130	205	324	1,603	311	427	201	357	627	220	0
Pseudomonas asiatica	5	3	220	405	174	115	5	90	422	345	365	7	2,873	11,038	2,557	26
Sphaerotilus natans	3,171	2,897	2,632	1,998	1,602	1,822	460	305	1,024	196	337	477	273	487	263	4
Bacteroides cellulosilyticus	2,200	1,279	3,571	1,987	1,947	2,400	139	580	1,071	247	468	210	575	411	175	0
Salmonella enterica	2,281	1,917	1,126	2,115	3,142	2,592	202	176	1,098	247	393	251	205	651	263	0
Acetoanaerobium sticklandii	1,561	899	2,282	2,205	5,402	2,967	33	52	439	48	131	44	62	118	39	0
Klebsiella quasipneumoniae	1,174	1,163	2,619	3,728	1,354	3,210	133	148	1,171	137	234	189	171	276	149	5
[Ruminococcus] gnavus	2,307	1,581	1,666	2,007	1,802	2,628	186	239	1,045	214	352	279	287	444	188	4
Pseudomonas guangdongensis	84	186	561	439	418	586	27	62	265	417	1,872	131	1,343	4,423	4,184	105
Dechloromonas sp.	1,046	1,571	2,185	2,624	2,284	1,506	177	266	1,117	180	250	248	212	525	205	2
Acidovorax carolinensis	1,355	1,874	2,246	2,013	1,785	1,330	205	306	990	219	239	347	331	541	283	3
Akkermansia muciniphila	1,318	1,393	1,067	1,821	1,679	1,201	394	358	1,507	416	320	423	465	545	233	10
Enterobacter hormaechei	1,317	1,518	923	1,607	2,679	2,395	122	222	653	235	500	139	224	461	150	2
Bacteroides sp. HF-162	1,711	1,001	1,368	1,846	1,851	1,749	184	273	984	306	311	164	264	403	184	1
Bacteroides sp. HF-5287	1,653	1,096	1,459	1,560	1,840	1,729	126	270	983	275	335	147	251	427	204	1
Bacteroides sp. A1C1	1,502	1,025	1,192	1,767	1,803	1,706	140	261	976	246	319	204	266	434	180	0
Aquaspirillum sp. LM1	835	1,242	3,035	1,788	1,893	1,368	133	176	457	73	120	151	219	248	117	0
Alistipes onderdonkii	1,288	861	1,121	1,246	1,373	2,036	218	312	1,017	307	594	328	341	473	194	0
Aeromonas sp. WP2-W18-CRE-05	1,892	2,196	1,135	1,934	1,497	1,349	165	75	524	71	109	122	81	450	68	2
Bacteroides sp. CACC 737	1,457	998	1,267	1,543	1,758	1,615	137	264	894	254	300	190	260	425	178	0
Bifidobacterium adolescentis	1,544	1,004	1,106	1,800	1,525	1,251	169	261	1,137	181	259	257	321	474	186	0
uncultured human fecal virus	1,565	927	1,114	1,614	1,502	1,829	96	189	1,049	234	301	124	202	430	177	0
Pseudomonas otitidis	74	171	169	322	284	455	6	29	177	175	658	28	417	3,023	5,036	127
Acidovorax sp. JMULE5	1,074	1,425	1,572	1,513	1,340	1,006	183	220	706	120	201	314	495	621	311	0
Enterocloster bolteae	1,858	1,223	1,267	1,347	1,269	1,568	155	182	760	232	218	167	223	332	179	4
Desulfovibrio sp. 86	1,499	874	1,276	941	2,546	2,119	126	150	391	81	232	164	132	204	115	2
Shewanella baltica	1,161	1,259	1,502	1,728	1,771	1,683	69	98	568	77	107	125	125	238	81	0
Bacteroides intestinalis	1,472	1,435	1,174	1,069	1,778	1,671	98	155	566	209	229	136	167	265	110	0

**Enterobacter roggenkampii**	2,240	2,068	730	925	2,803	304	113	227	535	52	61	70	264	87	49	0
**Roseburia intestinalis**	1,253	952	1,394	1,312	1,396	2,037	127	157	683	176	230	158	235	312	105	0
**Dechloromonas aromatica**	639	1,156	1,704	2,149	1,617	975	106	166	817	97	145	146	184	425	171	1
**Bifidobacterium longum**	1,656	1,061	1,114	1,301	1,383	1,184	162	172	825	174	197	225	227	420	149	2
**Lachnospiraceae bacterium**	1,497	792	1,110	1,496	1,506	1,539	67	144	906	161	218	89	189	394	137	1
**Pseudomonas stutzeri**	324	713	440	837	655	662	71	93	518	228	658	138	539	2,039	1,795	37
**Sulfurospirillum cavolei**	982	2,030	1,621	1,177	1,091	1,119	242	163	422	51	149	219	155	190	68	0
**Acidovorax sp. RAC01**	838	1,318	1,520	1,479	1,287	878	148	194	663	112	181	227	244	350	162	1
**Acinetobacter johnsonii**	697	1,745	1,265	953	714	897	178	220	480	57	170	216	902	512	567	9
**Acinetobacter pittii**	238	833	495	758	824	1,416	102	98	339	66	392	100	127	800	2,778	125
**Arcobacter skirrowii**	685	1,694	1,824	1,778	1,381	1,065	59	87	414	56	81	85	85	140	38	0
**Azotobacter chroococcum**	70	140	270	334	501	384	13	31	190	234	766	64	596	3,035	2,566	58
**Lachnospiraceae bacterium GAM79**	1,138	796	1,019	1,064	2,056	1,169	94	97	518	222	185	101	145	244	215	5
**Enterobacter cloacae**	985	1,080	1,125	1,434	1,330	1,289	74	170	463	119	167	153	131	209	104	2
**Ruminococcus sp. SR1/5**	1,213	684	903	1,131	1,069	1,325	123	171	818	197	238	155	220	367	124	0
**Bifidobacterium pseudocatenulatum**	1,171	1,012	931	1,128	1,171	904	179	191	735	181	144	286	204	314	111	2
**[Ruminococcus] torques**	1,256	627	838	1,156	1,147	1,442	81	148	685	227	197	92	159	314	147	0
**Anaerobutyricum hallii**	1,052	834	995	1,251	1,049	1,407	86	167	725	159	173	105	125	262	89	0
**Flavonifractor plautii**	1,291	740	1,013	1,413	1,294	1,252	85	87	576	108	144	79	116	177	64	2
**Bacteroides sp. M10**	1,368	911	914	1,239	1,024	1,139	129	136	590	144	165	158	165	241	97	2
**Akkermansia glycaniphila**	1,617	1,328	1,155	558	783	830	308	153	340	88	237	438	140	186	90	2
**Blautia sp. SC05B48**	1,184	613	827	1,073	1,031	1,218	97	159	771	162	186	135	212	384	139	0
**Epilithonimonas vandammei**	706	1,016	2,142	947	737	791	156	150	532	115	234	173	148	219	108	2
**Pseudomonas sp. LTGT-11-2Z**	2	2	34	99	265	285	0	7	71	159	573	1	222	3,455	2,907	53
**Thauera sp. MZ1T**	928	661	804	1,261	1,217	933	151	177	742	133	210	206	129	400	150	10
**Fusobacterium mortiferum**	1,318	717	1,075	1,078	1,187	1,505	84	96	402	72	172	70	107	161	59	0
**Collinsella aerofaciens**	1,362	715	731	1,136	1,278	1,125	86	105	594	144	147	124	153	244	140	0
**Lachnospiraceae bacterium sunii NSJ-8**	1,250	672	962	835	1,544	1,206	68	87	492	152	163	130	145	225	129	0
**Enterococcus faecium**	833	760	1,669	1,058	971	1,154	81	148	533	88	135	69	184	210	70	0
**Lachnospira eligens**	1,039	1,089	975	1,173	1,027	1,065	120	86	513	91	129	141	116	217	61	0
**Diaphorobacter sp. JS3051**	596	821	971	783	2,353	465	72	133	461	95	106	162	128	240	108	0
**Klebsiella variicola**	1,100	439	2,188	689	587	299	42	442	477	77	50	97	564	215	89	0
**Phage FAKO05_000032F**	1,055	311	989	1,806	711	672	24	143	1,000	61	107	36	79	184	55	0
**Phascolarctobacterium faecium**	1,001	573	707	1,067	1,105	1,121	101	110	546	122	170	99	141	232	101	0
**Acidovorax sp. T1**	637	952	1,177	1,146	911	619	110	157	511	96	121	148	159	272	156	0
**Arcobacter lanthieri**	349	1,300	1,012	1,334	1,479	944	32	46	312	31	78	39	56	108	36	0
**Sulfurospirillum sp. ACSDCE**	280	2,585	1,096	781	536	1,087	89	65	280	21	47	97	56	93	20	0
**Comamonas testosteroni**	697	889	1,066	1,026	870	647	121	157	509	106	129	235	190	278	127	0
**Blautia obeum**	959	530	839	904	803	1,066	113	151	627	155	175	90	162	306	97	0
**Aeromonas sp. ASNIH2**	432	1,308	1,286	1,453	1,151	311	45	57	441	30	57	36	85	214	30	0
**Sulfurospirillum multivorans**	466	1,266	1,655	744	582	778	187	137	354	41	118	197	145	159	36	0
**Ruminococcus bicirculans**	1,447	867	759	695	707	1,377	52	69	314	70	113	85	120	109	59	0
**Odoribacter splanchnicus**	972	670	768	883	866	924	83	126	539	145	189	133	112	268	78	0
**uncultured prokaryote**	927	793	909	692	785	1,024	104	117	398	77	143	108	120	289	172	0
**Pseudomonas nitroreducens**	60	94	191	349	542	396	17	38	221	97	146	30	371	1,997	1,926	29
**Ruthenibacterium lactatiformans**	1,024	676	886	849	1,031	913	91	67	365	73	100	92	66	159	44	0
**Raoultella ornithinolytica**	980	796	662	939	767	674	79	111	499	90	108	119	83	307	71	2
**Acinetobacter piscicola**	860	1,128	763	316	586	736	289	153	169	43	236	377	126	226	114	0
**Streptococcus salivarius**	838	423	601	711	947	1,236	86	117	433	115	186	64	93	181	81	2
**Delftia acidovorans**	596	760	795	890	730	583	88	131	455	104	127	148	159	308	167	2
**Acinetobacter haemolyticus**	312	2,089	381	502	307	468	199	74	338	37	126	305	104	517	256	4
**Citrobacter sp. CF971**	260	241	1,625	360	1,345	1,752	15	37	44	18	26	30	55	152	10	0
**Aliarcobacter faecis**	423	985	942	1,272	1,010	644	49	57	288	36	62	39	43	91	26	0
**Acinetobacter sp. CS-2**	508	326	864	607	1,706	272	43	70	371	52	41	252	251	426	165	2
**Dechloromonas sp. HYN0024**	409	611	922	1,192	871	595	65	108	438	74	93	115	90	230	65	0
**Butyricimonas faecalis**	842	524	701	774	767	823	89	156	369	92	148	95	147	177	72	1
**[Arcobacter] porcinus**	422	1,168	897	589	1,072	707	24	47	313	28	58	39	50	97	22	0
**Azospira sp. I09**	479	597	694	881	720	606	73	96	297	39	84	130	153	266	381	5
**Clostridiales bacterium CCNA10**	848	467	603	777	862	773	60	71	434	102	109	38	90	146	49	1
**[Eubacterium] siraeum**	1,298	423	529	694	633	674	68	77	388	88	112	59	104	142	50	0
**Clostridioides difficile**	756	473	612	783	693	799	62	86	409	76	125	86	103	187	69	0
**Acidovorax avenae**	479	658	773	724	713	512	83	124	412	84	103	139	146	214	98	3
**Paracoccus yeei**	497	495	410	417	494	459	138	240	571	280	259	222	156	360	257	2
**Paraprevotella xylaniphila**	734	438	652	695	728	703	77	125	410	125	99	85	120	151	71	1
**Chryseobacterium manosquense**	840	545	814	1,003	655	526	48	53	420	13	26	40	51	101	12	0
**Klebsiella aerogenes**	695	1,008	779	469	543	515	101	118	247	49	105	133	153	129	34	0
**Bacteroides sp. CBA7301**	570	1,036	716	507	545	678	124	85	261	43	70	145	99	95	51	2
**Bacteroides sp. HF-5141**	651	537	608	655	648	723	64	95	336	78	129	75	82	155	45	0
**Sulfurospirillum deleyianum**	292	1,260	1,009	700	437	413	83	79	246	22	48	105	77	92	17	0
**Veillonella parvula**	760	373	1,001	568	412	608	48	179	348	54	103	54	148	154	41	0
**Chryseobacterium sp. POL2**	672	1,025	1,367	237	594	459	31	47	169	26	41	41	49	64	12	0
**Pseudomonas citronellolis**	60	99	145	201	206	261	11	23	170	99	326	50	302	1,431	1,423	19
**butyrate-producing bacterium SS3/4**	700	418	641	668	618	761	38	80	386	61	87	54	103	136	48	0
**Acidaminococcus intestini**	604	409	599	703	702	462	102	113	428	86	91	74	137	136	62	2
**Parabacteroides sp. CT06**	692	535	582	749	655	560	54	91	355	74	93	57	65	108	39	0
**Arcobacter suis**	238	743	595	783	867	681	39	42	338	48	69	35	43	102	22	0
**Acidovorax sp. HDW3**	414	572	677	684	602	425	67	130	330	56	107	143	115	197	90	0
**Acinetobacter seifertii**	272	897	482	508	337	466	103	70	269	39	88	147	126	424	364	3
**Citrobacter sp. LUTT5**	460	147	62	497	2,259	946	20	6	63	17	16	16	6	31	17	0
**Sulfurospirillum halorespirans**	450	944	854	526	376	530	105	104	220	18	55	111	112	94	29	0
**Arcobacter cloacae**	379	681	637	867	721	550	52	47	251	30	55	55	68	87	45	0
**Amniculibacterium sp. G2-70**	651	988	817	502	371	383	85	69	269	26	33	114	58	116	35	0
**Acidovorax monticola**	391	549	635	595	563	417	75	90	323	48	98	129	205	256	129	5
**Dysosmobacter welbionis**	649	387	536	652	681	660	60	56	331	104	85	48	80	119	53	0
**Bacteroides caecimuris**	604	432	563	651	615	642	61	84	327	74	100	69	89	120	57	1
**Acinetobacter indicus**	330	770	491	609	371	454	110	83	332	35	87	152	75	347	238	0
**Pseudomonas mendocina**	147	196	195	443	345	322	48	40	240	94	323	60	228	924	831	12
**Phocaeicola salanitronis**	602	418	535	580	597	770	47	78	327	85	125	51	69	116	46	0
**Arcobacter sp. L**	249	665	649	906	824	592	30	30	254	23	52	19	41	67	23	0

Alistipes shahii	664	360	449	462	531	465	83	151	412	122	140	155	154	177	85	0
Enterobacter kobei	299	212	221	378	1,467	1,159	16	39	266	44	57	32	49	119	34	0
Paracoccus pantotrophus	444	385	316	287	392	398	112	201	463	177	286	201	136	313	217	1
Ochrobactrum anthropi	401	392	294	423	454	525	78	162	501	168	279	86	147	237	153	2
Aeromonas salmonicida	393	388	406	858	698	388	85	91	373	70	102	83	108	163	65	0
Acidovorax sp. JS42	327	499	674	558	584	345	53	102	461	72	81	99	106	197	71	0
Azotobacter vinelandii	48	68	122	167	222	173	11	21	105	115	311	24	279	1,417	1,082	38
Acinetobacter sp. WCHA45	148	194	290	193	171	208	33	58	98	21	54	44	122	1,579	903	7
Paracoccus denitrificans	428	379	254	286	376	321	132	180	456	179	230	206	140	301	222	2
Paracoccus aminovorans	433	268	259	274	379	359	131	197	502	180	275	157	130	310	215	0
Diaphorobacter sp. HDW4A	365	570	660	539	578	345	73	102	274	53	67	74	94	143	58	2
Acinetobacter bereziniae	191	499	464	355	307	453	81	149	215	36	215	188	168	348	254	9
Oscillibacter sp. PEA192	562	340	434	589	583	608	38	79	267	68	81	32	66	105	46	1
Stenotrophomonas maltophilia	401	423	477	491	448	407	64	98	304	60	99	118	82	239	135	4
Serratia sp. ATCC 39006	628	651	508	565	508	579	3	5	268	10	29	9	22	31	2	0
Serratia marcescens	443	627	391	447	446	457	44	61	360	47	115	68	55	149	94	2
Acinetobacter schindleri	271	597	785	405	245	475	57	60	198	48	54	69	59	368	78	2
Acinetobacter lwoffii	223	745	423	496	292	380	113	71	242	13	68	118	87	346	122	3
Pseudoarcobacter acticola	193	410	399	950	950	249	39	61	187	33	49	59	33	73	27	1
Megasphaera elsdenii	564	346	400	501	502	519	33	73	275	79	90	60	67	120	62	2
Alistipes finegoldii	456	235	445	480	449	412	56	165	316	77	140	86	121	167	63	0
Bacteroides helcogenes	643	325	473	440	541	678	34	41	168	39	57	47	65	79	18	0
Vibrio vulnificus	204	195	166	160	1,342	901	46	26	361	15	31	18	25	89	16	0
Dechlorosoma suillum	242	333	426	641	472	362	27	43	167	31	49	50	118	215	392	4
Prevotella intermedia	454	403	473	570	395	419	63	62	269	50	76	68	94	128	42	0
Roseburia hominis	445	219	388	638	471	480	24	59	411	50	52	23	66	144	42	1
Bacillus subtilis	182	80	154	110	173	161	122	442	568	290	334	129	227	366	156	2
Desulfovibrio sp. G11	564	400	456	405	725	463	54	33	108	20	54	36	33	75	23	0
Coprobacter sp. 2CBH44	455	282	352	513	488	486	44	85	284	51	98	50	72	120	45	0
Azotobacter salinestris	27	38	87	119	153	131	8	9	58	87	257	29	214	1,181	967	28
Diaphorobacter sp. JS3050	278	472	469	475	424	354	45	91	262	54	72	93	70	145	74	2
Comamonas terrigena	300	408	610	575	370	286	55	53	194	46	40	68	68	165	127	0
Coprococcus catus	472	265	396	466	380	526	43	77	287	49	81	55	90	137	36	0
Arcobacter defluvii	218	582	449	479	801	344	19	29	179	15	38	24	37	66	22	0
Ligilactobacillus salivarius	519	303	327	417	579	493	15	21	239	37	52	45	39	108	39	2
Aeromonas sp. ASNIH3	437	573	397	476	437	377	39	48	144	25	59	52	55	66	41	0
Sulfurospirillum sp. JPD-1	157	926	663	361	268	359	50	48	121	11	43	64	45	50	9	0
Malaciobacter pacificus	319	509	525	442	299	553	35	70	188	20	56	56	42	47	12	0
Pulveribacter suum	285	396	450	394	351	326	56	87	270	55	89	90	62	164	92	1
Vogesella sp. LIG4	392	142	141	571	674	489	33	27	324	52	65	27	17	133	62	0
Arcobacter trophiarum	213	476	448	625	596	375	15	34	171	21	36	27	31	50	14	0
Opitutaceae bacterium TAV5	500	378	422	345	427	461	42	48	144	21	73	69	65	82	29	1
uncultured crAssphage	429	203	224	777	459	452	19	37	264	44	59	12	13	52	25	0
Lacrimispora saccharolytica	389	308	481	377	436	477	29	53	174	44	48	34	85	91	34	1
Diaphorobacter polyhydroxybutyrativorans	272	389	430	388	356	299	49	76	219	54	78	85	68	148	135	4
[Clostridium] innocuum	411	334	605	382	307	347	34	64	169	40	56	55	97	108	33	0
Variovorax paradoxus	289	359	408	420	378	283	36	79	235	57	76	85	69	142	81	0
Klebsiella michiganensis	471	308	371	388	295	396	58	70	170	29	73	60	47	185	33	0
Prevotella melaninogenica	403	387	368	359	278	461	51	70	217	40	59	48	77	86	44	0
Melaminivora sp. SC2-9	267	330	426	404	327	239	58	93	252	60	74	75	85	143	77	0
Citrobacter freundii complex sp. CFNIH9	1,125	375	52	56	308	848	12	0	17	7	23	10	3	7	4	0
Acinetobacter towneri	238	297	372	234	178	238	45	61	130	17	106	181	207	344	159	0
Shewanella oneidensis	356	357	264	319	532	750	11	7	129	5	12	8	8	41	6	0
Bacteroides sp. PHL 2737	447	247	309	387	379	464	28	47	192	60	51	35	56	76	21	0
Candidatus Accumulibacter sp.	251	332	394	421	492	359	54	54	170	22	42	40	37	94	22	0
Citrobacter sp. RHBSTW-01013	1,626	561	26	44	407	28	5	3	49	2	9	1	0	5	0	0
Alistipes communis	337	180	361	349	291	306	45	91	318	93	65	60	93	123	37	0
Pseudomonas sp. 13159349	3	3	37	64	36	14	0	8	62	60	60	1	546	1,414	434	4
Lacunisphaera limnophila	476	352	362	312	294	398	46	57	121	26	64	72	52	78	18	0
Lachnospiraceae bacterium Choco86	384	196	304	361	362	439	26	47	251	59	51	43	55	96	39	0
Fusobacterium ulcerans	275	291	344	613	257	246	31	37	305	19	53	35	50	85	14	0
Thauera humireducens	283	284	291	364	381	299	40	66	204	59	75	61	55	116	62	1
Aeromonas sp. ASNIH7	399	188	185	336	406	241	40	52	251	83	30	43	130	172	77	0
Aeromonas sp. ASNIH1	167	255	530	528	355	255	29	61	175	17	53	33	41	100	21	0
Comamonas thiooxydans	255	344	393	385	291	216	45	43	207	49	35	88	53	142	66	1
Bifidobacterium catenulatum	301	282	310	302	355	327	53	81	182	55	35	68	66	118	57	0
Enterococcus faecalis	194	258	496	571	253	230	30	38	286	20	39	23	58	72	17	0
Stenotrophomonas acidaminiphila	241	313	296	313	306	253	55	79	221	50	60	80	44	161	76	0
Chryseobacterium haifense	203	463	636	352	212	208	32	29	142	19	35	27	25	59	14	0
Blautia producta	359	271	288	350	255	384	28	51	179	27	51	31	41	97	29	0
Opitutus terrae	366	335	318	261	335	352	34	41	104	17	64	53	57	62	30	0
Ralstonia solanacearum	243	258	341	322	339	280	39	46	156	23	46	62	51	128	60	2
Pseudomonas chlororaphis	89	92	124	203	176	149	23	21	118	44	155	24	131	599	417	7
Alistipes dispar	366	204	211	250	351	266	55	57	199	64	89	59	54	96	47	1
Sulfuricurvum kujiense	87	901	716	246	63	240	13	7	37	0	8	9	17	16	3	0
Lachnospiraceae bacterium NSJ-4	348	218	316	244	233	512	34	36	129	19	60	36	46	64	26	1
Acidovorax ebreus	208	302	310	312	274	216	55	63	188	38	61	74	61	99	54	0
Bifidobacterium breve	281	216	354	291	206	414	20	57	171	34	56	39	64	72	24	0
Elizabethkingia anophelis	336	377	368	275	255	241	32	32	126	15	101	33	30	52	19	0
Micropruina glycogenica	140	157	122	134	156	135	108	83	369	114	199	95	83	277	108	2
Sulfurospirillum sp. SL2-1	329	414	557	406	120	158	47	17	78	9	27	44	30	38	7	0
Pseudomonas balearica	166	139	172	195	190	197	21	27	93	45	132	43	124	393	313	9
Nibricoccus aquaticus	355	306	281	238	273	352	55	26	99	22	49	58	53	58	21	1
Enterocloster clostridioformis	252	245	231	357	356	300	18	25	181	45	43	20	44	85	29	2
Opitutus sp. GAS368	384	328	259	238	251	346	38	23	98	15	53	58	42	58	20	1
Pelobacter propionicus	169	61	135	199	557	573	11	40	105	38	89	23	22	85	41	0
Pseudomonas fluorescens	136	178	162	223	182	187	20	23	97	42	104	30	110	344	294	3
Pseudoxanthomonas mexicana	187	208	248	244	256	154	58	86	228	69	81	42	50	154	67	0
Pseudomonas oleovorans	149	132	137	207	164	157	35	26	132	38	138	35	106	373	293	7

**Thauera sp. K11**	170	212	268	330	311	268	33	53	158	22	40	43	53	101	62	1
**Azoarcus olearius**	170	218	275	272	320	256	25	54	142	33	56	59	36	118	79	3
**Klebsiella oxytoca**	406	198	248	282	307	317	22	27	109	25	40	22	42	46	20	0
**Novosphingobium sp. ABRDHK2**	42	40	40	40	44	41	8	11	32	10	22	123	168	248	1,234	4
**Tessaracoccus aquimaris**	237	193	261	212	215	201	70	73	198	60	62	54	85	127	53	1
**Rugosibacter aromaticivorans**	200	254	337	359	423	213	23	23	106	11	20	34	31	48	12	0
**Azoarcus communis**	158	247	317	326	287	224	36	43	162	38	40	26	45	77	51	1
**Delftia tsuruhatensis**	152	219	314	385	229	223	23	52	134	25	42	36	48	93	101	0
**Chryseobacterium sp. NEB161**	374	354	272	291	240	214	26	30	98	20	36	16	36	53	7	0
**Erysipelatoclostridium ramosum**	303	261	189	332	189	330	20	17	187	38	50	34	22	62	7	2
**Klebsiella sp. WP7-S18-CRE-02**	201	27	16	234	26	1,482	4	1	12	1	7	6	4	5	4	0
**Sulfurospirillum sp. ACSTCE**	177	248	291	241	247	251	66	54	124	23	47	97	58	58	27	0
**Erysipelothrix phage phi1605**	11	1,836	89	3	6	50	2	4	2	1	0	2	1	1	0	0
**Haemophilus parainfluenzae**	216	272	189	200	240	301	28	65	210	40	61	35	51	68	31	0
**Pseudomonas multiresinivorans**	28	45	61	103	148	105	5	12	70	28	60	14	127	533	626	16
**[Clostridium] scindens**	333	197	200	236	264	301	28	25	110	30	45	30	52	59	41	0
**Bacteroides heparinolyticus**	268	216	250	275	263	315	32	34	102	21	40	28	36	55	15	0
**Shinella sp. HZN7**	182	161	196	176	186	165	61	75	222	60	105	67	51	155	84	0
**Comamonas kerstersii**	240	268	247	301	292	154	28	42	141	35	28	35	32	67	15	0
**Pseudomonas knackmussii**	37	61	79	117	110	86	4	16	78	42	169	19	127	497	418	8
**Azoarcus sp. DD4**	171	206	270	255	269	204	29	60	109	21	50	28	30	95	62	4
**Oryzomicrobium terrae**	137	217	296	306	283	227	19	39	110	17	38	42	38	49	36	0
**Coprococcus sp. ART55/1**	196	113	420	249	277	201	8	57	109	20	29	17	86	44	18	1
**Sulfurospirillum sp. SL2-2**	91	533	219	172	170	352	32	23	73	17	26	50	30	46	5	0
**Klebsiella grimontii**	218	190	160	273	290	238	22	41	169	23	24	24	24	113	20	1
**Streptococcus thermophilus**	240	188	171	217	257	262	29	41	186	38	41	28	31	68	18	0
**Clostridium perfringens**	191	96	154	71	95	156	27	194	171	100	209	65	123	119	37	2
**Paludibacter propionicigenes**	334	270	270	218	254	252	19	12	62	7	24	34	16	27	5	0
**Pseudomonas mosselii**	28	24	67	48	33	44	5	27	68	83	85	18	648	360	246	7
**Ottowia oryzae**	188	183	229	179	242	175	44	52	169	42	84	51	50	57	41	0
**Acinetobacter sp. NEB 394**	123	367	212	216	115	202	38	44	97	17	37	59	69	117	68	0
**Citrobacter sp. RHBSTW-00524**	23	313	32	29	219	1,045	6	9	24	8	25	15	7	11	8	0
**Candidatus Accumulibacter phosphatis**	177	210	281	272	335	200	21	30	78	9	31	42	26	38	18	3
**Pseudomonas sp. TUM18999**	23	38	65	103	90	100	9	12	72	38	137	19	117	490	443	8
**Acinetobacter junii**	139	243	189	153	159	130	26	42	84	14	20	39	23	326	170	2
**Streptococcus parasanguinis**	210	227	201	148	318	334	24	14	109	27	39	17	20	51	17	0
**Prevotella oris**	205	298	216	190	159	312	31	26	126	17	40	38	37	43	16	0
**Barnesiella viscericola**	345	203	205	323	150	147	21	27	189	17	19	13	23	48	21	0
**Citrobacter sp. LY-1**	1,013	166	22	85	294	43	6	5	17	8	29	8	2	17	11	0
**Flintibacter sp. KGMB00164**	296	134	187	218	259	273	20	26	117	20	39	17	22	49	16	0
**Flavobacterium haoranii**	173	332	397	276	143	121	20	18	106	9	12	30	25	14	14	0
**Clostridiales bacterium**	272	179	222	242	236	215	22	26	99	20	22	20	33	50	21	0
**Achromobacter xylosoxidans**	152	189	200	205	243	190	32	38	91	31	54	45	49	88	57	2
**Lachnoclostridium sp. YL32**	193	186	174	323	222	174	31	17	170	44	10	23	29	56	12	0
**Thauera chlorobenzoica**	162	183	231	235	256	182	22	33	105	21	36	42	28	65	48	4
**CrAssphage cat_SB2894**	319	117	183	286	257	206	11	10	150	13	21	16	15	30	10	0
**Oscillibacter valericigenes**	317	206	212	182	294	214	13	21	70	10	14	19	28	29	13	1
**CrAssphage GF1-2_000079F**	245	110	151	342	271	197	14	13	180	25	30	8	11	29	13	0
**Bacteroides zoogleoformans**	241	187	231	199	183	276	19	39	96	19	43	29	23	37	9	0
**Romboutsia ilealis**	120	81	181	170	125	109	42	119	203	86	68	42	73	144	67	0
**Stenotrophomonas rhizophila**	38	35	48	71	71	140	7	9	38	38	89	10	108	545	374	5
**Pseudomonas sp. K2W31S-8**	52	47	51	126	158	115	7	11	77	36	123	24	102	323	362	10
**Providencia rettgeri**	342	301	136	320	172	162	14	18	61	9	11	15	13	38	11	0
**Bifidobacterium bifidum**	222	173	162	182	227	248	26	40	131	37	48	24	26	48	25	0
**Desulfovibrio vulgaris**	156	118	306	181	240	159	16	65	129	19	38	26	48	69	24	0
**Acidovorax sp. 16-35-5**	186	200	205	233	181	155	30	42	111	22	38	58	34	76	20	0
**Leclercia adecarboxylata**	133	128	166	236	404	217	15	16	98	19	21	18	31	55	21	0
**Chryseobacterium indologenes**	207	314	302	233	131	139	28	28	96	13	15	13	16	33	8	0
**CrAssphage ES_ALL_000190F**	227	91	96	702	132	99	10	9	132	6	21	5	5	32	7	0
**Chryseobacterium taklimakanense**	141	168	237	543	156	121	37	20	51	9	21	28	16	22	4	0
**Simplicispira suum**	150	201	245	211	205	180	22	34	103	31	33	31	34	52	36	0
**Citrobacter amalonaticus**	187	318	106	105	298	275	36	18	75	9	23	42	17	35	20	0
**Polaromonas sp. JS666**	203	185	197	217	339	198	8	14	89	4	19	17	18	34	16	0
**Comamonas sp. NLF-7-7**	153	206	196	191	193	136	31	48	131	38	39	50	25	80	35	1
**Thauera aromatica**	152	136	241	221	235	164	25	18	141	12	30	31	21	76	47	0
**Corynebacterium terpenotabidum**	76	55	114	108	155	171	58	83	189	109	127	53	49	143	54	0
**Pseudomonas denitrificans (nom. rej.)**	18	38	51	64	99	119	2	7	63	26	73	15	102	394	465	8
**Ereboglobus luteus**	235	214	197	197	211	228	22	17	57	6	26	39	43	34	13	1
**Citrobacter braakii**	184	197	100	152	164	483	25	26	50	17	53	21	16	19	14	0
**Cupriavidus metallidurans**	122	126	155	254	168	133	22	21	101	14	30	47	88	159	70	1
**Pseudomonas sp. S1-A32-2**	53	69	88	156	109	103	11	22	91	33	116	28	80	273	249	10
**Enterobacter ludwigii**	327	240	106	198	186	101	44	10	92	26	18	56	14	38	27	0
**Enterobacter asburiae**	156	172	126	220	219	251	24	20	80	21	47	19	36	54	11	2
**Klebsiella sp. STW0522-44**	159	259	90	99	167	549	8	10	39	7	10	20	6	18	16	0
**Ramlibacter tataouinensis**	117	170	175	192	193	128	24	39	143	31	38	55	34	73	45	0
**Alistipes indistinctus**	184	172	150	172	195	190	22	44	101	39	39	27	48	47	26	0
**Vibrio cholerae**	139	194	154	215	308	149	21	25	69	15	36	23	19	52	21	0
**Arcobacter aquimarinus**	106	288	200	231	248	174	9	15	75	9	18	13	20	20	5	0
**Aeromonas sp. ASNIH4**	88	174	145	195	264	214	10	32	78	14	44	18	37	78	36	1
**Pseudomonas sp. LPB0260**	32	53	60	106	94	100	12	13	52	32	116	21	122	320	291	3
**Thiomonas intermedia**	157	193	195	160	146	130	37	34	93	29	33	55	40	76	38	0
**CrAssphage LMMB**	195	76	127	309	242	232	18	10	116	7	18	10	8	28	10	0
**Ruminococcus champanellensis**	211	238	177	132	103	316	28	20	46	17	31	27	18	20	14	0
**Bosea sp. ANAM02**	119	97	98	114	111	64	44	100	198	55	79	74	48	122	68	2
**Verrucomicrobia bacterium IMCC26134**	234	186	176	141	159	215	30	17	63	11	35	26	25	38	12	0
**Veillonella nakazawae**	192	109	191	240	92	166	6	29	166	9	31	10	39	74	3	0
**Hydrogenophaga pseudoflava**	105	155	165	211	165	136	26	40	117	25	38	34	39	63	35	3
**Citrobacter sp. RHBSTW-00424**	142	43	22	50	988	84	1	3	4	1	5	4	2	6	1	0
**Arcobacter thereius**	130	218	240	250	186	176	12	20	49	7	15	7	9	27	1	0

**Acidovorax citrulli**	135	160	186	176	200	106	20	26	111	19	36	39	27	49	40	0
**Paracoccus kondratievae**	128	126	107	113	119	120	40	55	119	48	78	78	38	84	69	0
**Aeromonas simiae**	173	162	158	242	180	155	11	13	97	15	15	45	6	25	21	0
**Aeromonas jandaei**	181	135	307	197	115	135	16	26	73	12	22	7	28	39	22	1
**Azoarcus sp. CIB**	131	164	166	179	203	147	16	30	94	14	25	21	34	54	34	1
**Aeromonas sp. ASNIH5**	125	194	134	237	191	144	21	27	92	16	20	16	17	59	17	0
**Azoarcus sp. DN11**	125	171	196	173	191	146	19	26	72	11	26	30	16	54	47	2
**Vibrio anguillarum**	169	182	119	213	143	169	22	20	93	24	27	15	18	23	65	0
**Arcobacter venerupis**	62	164	234	250	217	167	6	11	93	11	12	11	20	22	10	0
**Acinetobacter sp. YH12138**	89	157	340	141	77	74	28	76	99	11	15	31	51	50	50	0
**Prevotella denticola**	128	150	132	290	98	117	16	10	164	15	23	34	16	77	14	0
**Verminephrobacter eiseniae**	125	173	190	167	156	144	21	25	90	21	28	35	25	57	26	0
**CrAssphage YS1-2_2437**	128	94	106	190	214	204	5	10	238	20	17	8	7	33	6	0
**Empedobacter brevis**	76	47	99	377	71	406	27	20	44	9	22	21	23	30	5	0
**Veillonella atypica**	106	83	201	293	112	167	14	30	141	13	29	3	40	26	13	0
**Oscillibacter sp. NSJ-62**	196	134	151	154	210	202	13	11	67	14	30	9	25	36	18	0
**Eggerthella lenta**	248	150	112	131	111	210	21	18	96	20	12	33	30	55	16	3
**Blautia argi**	213	95	125	145	149	263	8	18	110	23	27	12	22	39	15	0
**Hydrogenophaga sp. NH-16**	104	127	165	183	141	113	23	44	133	19	29	27	43	75	38	0
**Azoarcus sp. KH32C**	122	164	185	175	185	136	23	19	67	8	27	32	23	52	40	1
**Rhodoferax sediminis**	118	151	191	176	176	116	20	28	91	24	16	34	28	57	26	1
**Plesiomonas shigelloides**	185	155	211	104	209	190	13	12	70	6	21	11	18	37	10	0
**Sulfurimicrobium lacus**	112	123	210	188	232	130	21	24	79	14	18	36	11	37	16	0
**CrAssphage apr34_000142F**	253	98	99	214	173	150	9	16	150	13	28	9	6	21	8	0
**Bacteroidales bacterium CF**	281	129	183	125	174	187	10	15	49	7	18	24	17	13	7	0
**Citrobacter sp. RHBSTW-00127**	16	39	27	41	45	1,019	8	3	10	3	9	3	1	6	2	0
**Burkholderiaceae bacterium**	95	160	158	140	215	129	22	34	77	11	28	31	22	70	29	3
**Pectobacterium punjabense**	23	25	1,065	15	27	14	7	3	26	2	2	2	5	4	4	0
**Paracoccus zhejiangensis**	121	110	105	85	119	106	38	50	104	56	75	68	31	81	69	1
**Serratia plymuthica**	81	368	125	97	155	248	12	11	50	5	9	7	15	22	6	1
**Culex quinquefasciatus**	101	179	179	171	169	108	24	31	57	10	38	24	31	48	40	0
**Morganella morganii**	80	80	152	200	214	218	3	16	93	14	35	8	27	51	15	0
**Streptococcus dysgalactiae**	16	11	17	17	20	1,077	6	11	11	0	1	4	0	4	5	0
**Sulfurospirillum barnesii**	88	286	203	162	98	135	21	20	63	7	20	32	22	33	6	0
**Mycolicibacterium insubricum**	85	33	47	40	59	44	91	105	171	81	120	94	65	104	56	0
**Chromobacterium violaceum**	147	89	121	210	213	145	11	16	80	20	19	10	17	57	32	1
**Aromatoleum aromaticum**	127	160	165	151	187	109	18	30	61	16	27	32	25	52	18	0
**Anaerostipes caccae**	146	93	129	202	152	200	13	13	97	15	18	16	23	37	11	0
**Agrobacterium tumefaciens**	127	114	110	97	117	129	19	46	104	74	63	39	31	47	44	0
**Alistipes megaguti**	167	121	138	131	138	151	36	39	87	15	19	34	28	42	14	0
**Azoarcus sp. M9-3-2**	102	107	144	179	148	129	15	23	99	30	26	23	31	51	50	1
**Vibrio fluvialis**	114	149	95	240	203	126	9	8	90	8	17	19	13	38	16	0
**Citrobacter sp. RHBSTW-00903**	17	20	24	82	920	17	3	5	10	4	7	4	5	8	6	0
**Pseudomonas sp. ADPe**	20	22	35	65	51	43	1	6	41	24	75	10	65	336	331	6
**Prevotella ruminicola**	157	152	162	144	115	171	12	15	85	5	23	18	19	41	8	0
**Pseudomonas sp. ATCC 13867**	22	37	41	68	57	71	3	9	38	26	68	12	76	265	325	6
**Streptococcus sp. LPB0220**	134	155	115	128	156	226	24	12	59	16	21	12	27	25	13	0
**Caproiciproducens sp. 7D4C2**	204	159	136	119	158	157	16	15	45	9	18	17	13	34	10	0
**Candidatus Melainabacteria bacterium MEL.A1**	262	145	226	68	71	68	21	53	44	8	14	33	39	35	22	0
**Pseudomonas entomophila**	22	35	47	67	63	61	3	9	36	18	60	14	149	306	215	4
**Ralstonia pickettii**	99	119	152	137	126	90	41	42	65	14	29	47	41	68	32	1
**Faecalitalea cylindroides**	185	96	107	152	144	156	23	18	87	25	26	9	20	40	14	0
**CrAssphage ZA**	160	74	99	263	145	93	12	8	172	12	12	7	7	31	7	0
**Citrobacter koseri**	57	183	95	100	280	177	23	9	31	22	31	20	12	23	29	0
**Acinetobacter chinensis**	63	154	254	99	81	36	19	44	49	5	9	27	45	154	51	0
**Shewanella chilikensis**	156	384	57	46	209	89	8	17	38	4	14	12	17	25	11	0
**Rhodoferax sp. BAB1**	108	141	146	149	126	107	20	35	86	15	22	25	25	53	24	0
**Pseudomonas resinovorans**	16	33	41	57	60	60	2	6	46	25	84	15	58	276	293	7
**Amedibacterium intestinale**	182	89	129	122	178	138	14	19	84	17	39	8	10	33	13	0
**Pseudomonas furukawaii**	17	34	58	55	68	66	7	3	54	15	83	12	70	276	243	5
**Comamonas koreensis**	121	128	130	179	145	104	13	34	76	6	28	27	22	33	18	0
**Myroides odoratimimus**	139	112	190	118	182	107	22	26	58	6	12	26	24	26	13	0
**CrAssphage sp. C0531BW4**	145	92	93	243	161	139	6	4	87	19	14	11	11	26	9	0
**CrAssphage sp. C0521BD4**	197	69	63	346	96	128	5	2	84	12	12	10	3	22	6	0
**CrAssphage sp. C0526BW15**	155	65	84	271	141	125	11	16	86	13	17	8	6	42	3	0
**Acinetobacter sp. WCHA55**	78	194	133	162	74	92	17	23	53	12	18	28	25	69	62	0
**Sulfuritalea hydrogenivorans**	90	128	164	161	147	114	16	20	55	18	17	24	21	41	16	2
**Vibrio parahaemolyticus**	112	85	47	170	297	107	7	9	79	18	24	13	16	33	14	0
**Denitratisoma sp. DHT3**	118	128	157	132	164	98	11	23	71	10	11	18	25	40	24	0
**Zoogloea oryzae**	57	109	115	143	172	126	12	33	93	13	12	18	16	47	59	3
**Pseudomonas sp. BJP69**	6	7	29	19	12	277	0	3	24	11	13	4	57	409	152	0
**Pseudomonas fulva**	38	38	60	75	52	80	7	13	57	19	61	14	67	251	172	5
**Hydrogenophaga sp. PBC**	76	102	136	143	153	130	17	32	71	16	20	31	14	40	24	0
**Cruoricaptor ignavus**	193	237	223	168	93	45	6	6	21	0	3	2	0	3	4	0
**Proteus mirabilis**	101	144	169	127	99	116	18	22	56	12	37	24	28	30	19	0
**Leptothrix cholodnii**	94	97	136	128	140	109	20	19	72	30	25	22	13	72	24	0
**crAssphage cr6_1**	57	136	76	35	43	470	17	6	38	4	80	14	6	12	2	0
**Cupriavidus basilensis**	99	102	133	149	129	110	13	16	70	18	17	16	26	57	40	0
**Denitratisoma oestradiolicum**	124	144	123	150	138	113	10	15	50	20	22	23	16	38	7	1
**Lacrimispora sphenoides**	176	132	127	83	120	112	17	19	32	9	16	11	21	20	98	0
**Hydrogenophaga sp. PBL-H3**	73	128	157	119	124	88	18	27	81	21	27	24	21	55	28	0
**Acinetobacter ursingii**	59	156	89	101	116	133	22	20	53	8	23	23	26	93	60	2
**Prevotella dentalis**	106	119	128	151	108	135	10	16	83	12	28	23	19	31	8	0
**Clostridium butyricum**	95	143	59	300	52	35	20	26	134	7	7	23	10	57	4	0
**Dickeya zeae**	118	135	123	128	179	133	6	11	63	8	16	7	14	22	5	1
**butyrate-producing bacterium SM4/1**	149	75	106	137	127	156	13	22	59	15	18	17	21	36	16	0
**Pandoraea norimbergensis**	178	157	157	87	108	123	11	10	42	13	11	24	20	17	8	0
**CrAssphage FA1-2_000172F**	118	71	143	182	148	104	5	9	99	12	27	5	8	24	9	1
**Acinetobacter phage Acj61**	441	330	152	10	0	2	8	2	0	0	0	9	3	0	0	0

**Pseudoxanthomonas suwonensis**	95	54	65	73	65	66	26	52	152	41	66	26	22	108	41	1
**Rubrivivax gelatinosus**	77	90	103	115	97	97	17	34	97	20	30	25	20	72	34	1
**Pseudogulbenkiania sp. NH8B**	133	60	94	139	171	114	8	8	77	8	14	15	20	47	17	2
**Aquipluma nitroreducens**	174	123	136	101	156	115	12	11	31	9	14	16	8	16	3	0
**Pseudomonas protegens**	29	45	44	79	57	61	13	9	48	30	56	11	50	223	157	1
**Pseudomonas psychrotolerans**	29	64	55	65	55	64	5	9	47	15	75	9	55	172	186	7
**Desulfovibrio piger**	153	84	94	112	146	135	9	11	63	15	10	15	14	40	6	0
**Dialister massiliensis**	152	94	75	144	158	83	9	7	74	13	5	21	13	49	9	0
**Cellvibrio sp. KY-GH-1**	57	90	154	124	131	112	23	21	64	26	17	18	37	23	2	0
**Desulfobulbus propionicus**	140	91	62	82	106	128	24	26	64	20	27	30	21	51	22	0
**Pseudomonas sp. SCB32**	24	22	26	47	56	46	4	12	28	21	60	16	63	235	231	3
**Pseudomonas plecoglossicida**	4	14	15	47	24	19	1	2	31	16	74	3	74	348	221	1
**Bacteriophage sp.**	132	66	111	122	138	132	19	10	53	11	17	18	22	27	14	0
**Nakamurella multipartita**	48	36	34	33	38	58	55	30	129	72	94	66	29	93	75	0
**Lachnoclostridium phocaeense**	131	79	118	149	119	116	10	10	55	8	17	13	14	37	9	0
**Diaphorobacter sp. HDW4B**	70	111	120	116	95	100	18	23	70	21	37	21	23	43	10	0
**Dioscorea cayenensis**	11	13	63	34	23	12	11	15	32	11	12	8	124	452	55	2
**Dialister hominis**	145	103	68	151	133	73	9	8	80	18	10	19	16	35	9	0
**Fermentimonas caenicola**	141	113	121	117	125	159	10	4	31	8	7	17	7	7	9	0
**Citrobacter sp. 172116965**	700	8	7	11	85	30	1	4	4	2	7	0	2	6	4	0
**Pseudomonas sp. PONIH3**	6	10	12	17	10	19	0	4	23	18	24	5	152	463	105	0
**Veillonella dispar**	113	56	129	156	63	113	9	24	92	8	18	14	18	45	7	0
**Eubacterium sp. NSJ-61**	105	59	88	167	119	110	13	19	79	18	28	10	9	32	7	0
**Acidaminococcus fermentans**	83	70	99	194	104	72	1	15	102	19	13	11	27	36	17	0
**Shewanella benthica**	4	98	127	182	96	249	1	5	63	2	3	5	3	24	0	0
**Sterolibacterium denitrificans**	96	86	132	133	132	102	12	13	38	15	13	13	19	34	22	0
**Jeongeupia sp. USM3**	110	121	101	130	124	96	18	13	48	8	11	19	18	31	10	1
**Sterolibacteriaceae bacterium M52**	89	129	108	137	121	90	13	18	62	8	15	11	16	30	10	0
**Kinneretia sp. DAIF2**	81	79	93	90	79	76	20	30	87	23	21	26	32	70	48	0
**Microvirgula aerodenitrificans**	78	90	101	123	104	161	14	13	43	10	17	29	21	35	14	0
**Intestinimonas butyriciproducens**	127	83	91	106	151	131	9	10	62	9	23	9	10	18	10	0
**Acinetobacter sp. WCHAc010034**	74	144	135	82	70	80	32	26	54	8	13	41	12	50	27	0
**Muribaculaceae bacterium DSM 108610**	97	156	83	98	100	87	4	18	41	10	20	19	22	81	11	0
**Bordetella hinzii**	74	89	113	141	101	92	8	23	71	13	20	15	23	46	16	2
**Cupriavidus taiwanensis**	82	95	96	107	131	80	16	25	57	12	22	27	20	44	26	0
**Enterococcus avium**	125	81	183	133	70	69	11	11	68	7	8	14	23	32	4	0
**Bifidobacterium dentium**	96	70	191	55	138	126	11	18	28	14	22	10	35	13	12	0
**Diaphorobacter ruginosibacter**	84	112	126	95	105	92	9	18	58	17	20	18	22	44	17	1
**Comamonas piscis**	78	109	119	119	117	79	15	17	58	13	24	24	15	34	13	0
**Serratia fonticola**	118	154	116	107	71	103	5	8	61	5	11	14	12	30	14	0
**Rhodopseudomonas palustris**	124	86	78	70	112	65	13	23	66	20	30	29	26	55	28	0
**Chryseobacterium shandongense**	104	138	146	145	80	81	14	14	43	1	11	11	14	20	3	0
**Clostridia bacterium 12CBH8**	147	76	87	146	101	108	19	7	50	7	23	13	16	11	12	0
**Rhodoferax koreense**	76	89	104	110	113	79	11	18	65	16	13	21	11	63	33	0
**Riemerella anatipestifer**	95	134	146	157	78	82	12	14	42	2	9	5	18	20	7	0
**Acinetobacter sp. SWBY1**	41	129	123	44	56	75	14	23	45	8	20	60	59	90	30	0
**Diaphorobacter aerolatus**	58	97	112	142	96	85	13	17	64	13	23	21	16	36	22	1
**Desulfovibrio fairfieldensis**	130	100	117	77	171	125	11	9	27	1	7	9	13	15	2	0
**Methyloversatilis sp. RAC08**	63	85	81	89	86	67	18	17	48	9	12	28	17	46	147	1
**Fusobacterium varium**	69	47	194	112	97	109	5	30	53	12	18	5	25	28	6	2
**Vibrio metoecus**	54	53	134	295	30	162	1	9	33	3	8	6	7	10	4	0
**Clostridium bornimense**	129	136	113	56	67	161	18	14	41	12	11	20	15	12	2	0
**Aeromonas sp. 2692-1**	60	78	46	156	140	150	6	11	64	13	18	9	9	39	8	0
**Hydrogenophaga sp. BA0156**	67	74	126	79	106	75	12	20	74	9	20	20	40	54	20	0
**Bosea sp. Tri-49**	54	56	57	72	78	57	31	55	139	21	47	36	20	46	25	0
**Streptococcus suis**	118	73	69	135	93	118	11	16	52	10	26	14	17	29	10	2
**Pseudomonas oryzihabitans**	37	25	36	48	55	59	1	4	38	25	55	24	46	177	159	3
**Clostridium beijerinckii**	163	147	94	74	70	124	9	7	29	5	13	12	13	24	6	0
**Chryseobacterium taihuense**	89	134	130	116	111	80	10	16	47	2	16	10	3	22	4	0
**Pseudomonas syringae**	50	52	87	72	84	73	10	12	48	8	41	11	33	105	92	5
**Massilistercora timonensis**	95	73	86	131	96	128	15	12	68	14	14	8	11	22	7	1
**Pseudomonas lalkuanensis**	19	23	34	49	44	56	2	9	27	19	65	9	51	188	182	2
**Prevotella fusca**	100	118	122	82	74	95	7	15	64	12	23	15	18	24	9	0
**Lacticaseibacillus paracasei**	113	150	103	81	87	73	22	18	44	3	17	26	10	20	5	0
**Microlunatus phosphovorus**	44	24	44	29	44	43	19	34	184	52	57	31	40	93	30	0
**Polaromonas naphthalenivorans**	75	110	114	97	100	74	10	14	61	13	14	11	12	34	25	1
**Thermomonas sp. XSG**	97	78	81	83	47	95	16	27	76	13	22	41	24	46	18	0
**Acinetobacter venetianus**	58	105	106	112	53	76	14	17	67	4	15	18	17	59	42	0
**Aquabacterium olei**	55	100	98	103	92	87	15	21	66	12	19	17	26	38	13	0
**Aeromonas schubertii**	185	53	58	105	111	132	8	6	40	9	12	9	6	13	13	0
**Rhodobacter sphaeroides**	74	58	58	81	76	56	30	33	86	28	28	29	31	58	34	0
**Nitrosomonas europaea**	54	30	71	40	66	64	25	33	111	54	62	36	31	56	26	0
**CrAssphage sp. C0521BW15**	97	63	71	118	151	87	5	8	82	8	17	12	6	25	5	1
**Chromobacterium vaccinii**	80	79	70	127	126	104	7	12	49	9	13	9	15	35	21	0
**Acinetobacter nosocomialis**	58	98	76	73	71	69	13	20	39	9	15	18	22	87	81	1
**Xylophilus rhododendri**	74	96	108	96	95	70	17	10	53	7	22	24	18	33	26	0
**Petrimonas sp. IBARAKI**	91	79	94	98	109	89	9	23	59	24	20	8	15	18	9	0
**Edwardsiella ictaluri**	29	33	64	26	62	491	1	1	11	3	3	2	8	7	3	0
**Cupriavidus necator**	58	146	98	97	85	63	13	12	43	10	25	10	18	42	21	2
**Acinetobacter defluvii**	41	86	112	78	46	69	25	26	43	6	8	26	33	99	44	1
**Vitreoscilla filiformis**	95	96	105	103	77	84	24	15	44	9	14	16	17	29	12	0
**Blautia hansenii**	112	66	86	100	77	116	12	8	70	14	22	11	14	22	8	1
**Nitrosomonas sp. H1_AOB3**	49	65	54	52	57	60	17	35	118	43	47	46	29	44	20	0
**Yersinia enterocolitica**	64	126	69	156	111	89	5	7	57	5	6	8	6	21	4	0
**Rahnella aquatilis**	97	88	86	137	65	110	19	11	50	6	14	10	13	17	10	0
**Burkholderia vietnamiensis**	76	76	115	132	91	54	5	19	54	8	21	11	10	35	26	0
**Yersinia intermedia**	68	129	32	48	288	115	5	2	16	5	3	7	1	8	3	0
**Turicibacter sp. H121**	59	23	60	48	70	74	11	80	93	36	28	21	49	55	14	0
**Xanthomonas citri**	77	68	74	100	78	84	13	6	58	8	29	21	18	50	34	0

**Sinorhizobium sp. RAC02**	51	65	69	69	60	73	20	21	74	23	45	31	18	55	44	0
**Yersinia pestis**	114	83	48	75	138	128	6	8	39	10	8	10	8	21	14	0
**Pandoraea apista**	103	88	80	88	78	107	4	22	49	12	6	28	18	15	12	0
**Chlamydia suis**	73	200	79	74	33	87	17	21	56	12	16	5	13	8	14	0
**Sterolibacteriaceae bacterium J5B**	79	97	129	98	86	92	7	7	29	4	22	18	15	18	5	0
**Shewanella putrefaciens**	79	107	25	68	116	179	12	13	37	9	10	7	9	18	7	0
**Faecalibacillus intestinalis**	116	72	91	97	73	111	9	4	51	15	14	10	9	20	3	0
**Pseudomonas sp. TCU-HL1**	18	20	28	44	43	35	6	6	32	7	33	4	46	200	169	2
**Clostridium sporogenes**	105	65	84	84	107	91	11	18	45	27	15	6	15	17	2	0
**Schlegelella thermodepolymerans**	64	68	65	73	90	58	13	20	74	11	13	18	25	66	34	0
**Comamonas serinivorans**	62	90	80	95	89	75	9	19	57	13	18	15	17	35	14	0
**Acinetobacter sp. TTH0-4**	34	77	110	115	65	60	8	19	45	8	17	21	20	59	26	0
**Aminipila sp. JN-18**	106	144	100	54	97	87	3	4	24	4	13	16	5	17	7	0
**Burkholderia multivorans**	56	140	68	95	76	70	9	15	35	10	12	15	23	33	23	1
**Prevotella scopos**	82	106	91	70	62	100	26	12	52	16	17	14	14	15	3	0
**Aquitalea magnusonii**	55	65	107	86	146	101	7	4	52	5	8	15	10	12	6	0
**Alistipes sp. dk3624**	114	46	76	81	80	113	12	19	42	15	24	10	11	25	6	3
**Petrimonas mucosa**	81	76	89	96	123	94	10	5	31	5	17	17	8	17	6	0
**Ensifer adhaerens**	51	60	61	67	68	61	18	34	79	21	32	24	16	38	44	0
**Paraburkholderia fungorum**	23	16	26	15	20	14	41	57	58	61	61	106	76	53	45	1
**Prevotella sp. oral taxon 299**	127	107	90	65	68	80	12	7	45	7	13	14	15	17	5	0
**Tessaracoccus flavescens**	61	63	80	60	84	64	17	13	56	24	39	33	16	33	29	0
**Hydrogenophaga sp. BPS33**	82	59	103	90	87	57	17	17	57	16	10	16	13	36	11	0
**Chromobacterium phragmitis**	78	68	48	107	125	92	12	10	45	6	16	10	3	33	12	0
**Aquitalea sp. USM4**	59	93	53	179	76	93	6	7	43	12	12	4	1	18	9	0
**Variovorax sp. WDL1**	59	85	83	90	85	66	17	15	66	12	17	18	10	22	17	0
**Sphingobium yanoikuyae**	52	46	71	73	52	72	21	14	61	19	17	35	15	49	60	4
**Raoultella planticola**	74	71	98	96	64	74	12	22	40	3	13	16	45	19	10	0
**[Candida] glabrata**	42	75	100	65	52	64	38	29	52	36	18	22	24	34	6	0
**Hafnia alvei**	77	92	115	97	56	86	8	4	34	10	18	22	11	17	7	0
**Desulfovibrio magneticus**	88	64	80	66	112	110	7	14	41	13	7	9	6	23	9	0
**Pseudomonas sp. CC6-YY-74**	28	24	27	69	42	34	1	8	51	12	60	14	27	130	116	5
**Aquitalea sp. THG-DN7.12**	96	58	59	110	105	76	13	8	45	12	17	5	10	24	7	1
**Burkholderia cenocepacia**	63	78	79	75	91	61	16	10	35	7	18	18	19	39	32	1
**Pseudomonas veronii**	29	28	36	50	44	32	3	12	23	20	42	9	35	148	123	8
**Pseudomonas sp. FGI182**	3	7	9	15	16	11	1	6	11	21	26	0	65	324	126	0
**Limnohabitans sp. 63ED37-2**	67	88	111	86	92	69	5	9	31	11	16	18	6	21	10	0
**Acinetobacter sp. WCHAc010052**	27	71	90	53	50	36	16	22	44	3	5	22	28	103	67	0
**Streptococcus pneumoniae**	63	101	68	55	80	146	5	8	38	9	17	10	16	14	5	0
**Paracoccus aminophilus**	58	57	49	54	70	54	20	26	63	30	35	23	19	38	39	0
**Chromobacterium rhizoryzae**	86	78	80	82	113	78	9	5	45	9	12	5	11	15	5	0
**Vibrio tritonius**	48	181	107	24	26	187	6	6	27	1	4	5	6	4	1	0
**Propioniciclava sp. HDW11**	46	59	67	44	61	48	11	30	75	35	32	26	27	42	28	0
**Azoarcus pumilus**	65	74	87	76	93	85	16	12	33	6	7	15	11	28	21	0
**Acidovorax cattleyae**	56	87	74	88	93	60	9	18	36	8	13	13	14	43	16	1
**Rhizobacter gummiphilus**	65	57	71	75	75	51	10	22	66	10	16	17	16	48	27	1
**Prevotella jejuni**	118	97	78	70	72	58	10	14	48	9	6	12	16	11	7	0
**Acinetobacter dispersus**	29	162	79	51	38	60	5	11	28	2	11	14	12	67	55	1
**Halomonas sp. GFAJ-1**	114	131	80	4	44	243	0	0	1	0	0	1	1	1	0	0
**Vibrio furnissii**	101	113	45	88	74	85	7	7	40	6	9	12	10	16	5	0
**Empedobacter falsenii**	92	117	83	85	77	87	8	2	25	8	7	13	4	4	5	0
**Paracoccus sp. BM15**	64	67	43	45	56	58	17	25	58	30	42	28	25	34	24	0
**Sutterella megalosphaeroides**	84	27	50	121	103	56	8	9	68	17	11	10	16	22	8	0
**Pectobacterium carotovorum**	74	58	84	118	66	107	11	5	37	3	12	11	6	12	3	0
**Desulfovibrio carbinolicus**	72	54	86	75	126	83	1	8	23	13	15	4	12	24	9	0
**Sulfurimonas denitrificans**	38	111	124	78	73	91	7	11	32	3	5	12	9	7	2	0
**Yersinia kristensenii**	26	128	74	72	113	104	5	3	42	8	3	4	7	8	5	0
**Tessaracoccus defluvii**	62	65	67	77	66	46	18	19	55	19	21	19	18	25	15	0
**Klebsiella sp. WP3-W18-ESBL-02**	10	12	11	131	372	28	1	3	4	2	5	3	8	0	2	0
**Clostridium botulinum**	93	77	77	48	55	67	12	27	42	13	17	21	17	14	11	0
**Aeromonas sp. 1805**	15	42	45	91	123	148	5	4	38	10	32	1	4	18	11	0
**Thauera hydrothermalis**	55	64	78	74	97	61	11	13	48	5	10	18	14	24	14	0
**Longibaculum sp. KGMB06250**	89	56	71	82	77	102	4	6	52	9	11	4	6	10	6	0
**Prevotella enoeca**	69	103	83	78	52	94	7	10	36	5	6	17	8	15	2	0
**Caproiciproducens galactitolivorans**	112	74	64	64	89	77	7	3	37	5	7	17	8	11	9	0
**Mycolicibacterium phocaicum**	77	32	37	36	36	41	13	56	77	14	38	41	32	35	19	0
**Variovorax sp. HW608**	55	60	79	66	58	47	13	19	51	21	20	23	21	39	11	0
**crAssphage cr50_1**	53	219	53	77	38	26	18	7	44	7	3	18	7	8	4	0
**Enterobacter sichuanensis**	8	3	35	120	53	162	0	7	90	6	39	0	18	34	7	0
**Chitinolyticbacter meiyuanensis**	68	77	76	86	68	68	9	13	37	4	9	13	11	25	10	0
**Campylobacter jejuni**	84	73	66	51	60	78	13	13	46	5	14	13	13	34	10	0
**Hydrogenophaga sp. RAC07**	51	78	82	72	66	54	8	16	49	5	22	14	11	26	17	0
**Dehalococcoides mccartyi**	111	74	86	57	81	67	3	5	30	2	14	12	7	17	3	0
**Xanthomonas campestris**	32	94	130	71	96	33	4	9	26	4	14	16	8	16	16	0
**Hungatella hathewayi**	86	62	58	78	77	61	3	10	59	9	13	8	4	27	13	0
**Micropruina sp.**	64	47	46	46	48	66	20	21	73	20	29	22	14	34	18	0
**Pantoea vagans**	91	77	53	94	113	50	5	5	33	6	6	5	3	14	6	0
**Alcaligenes faecalis**	58	51	57	103	56	56	5	2	33	6	27	9	15	53	30	0
**Caproiciproducens sp. NJN-50**	106	77	63	80	89	75	21	4	21	0	6	3	1	11	1	0
**Rhodoferax saidenbachensis**	58	72	89	62	70	78	9	5	37	5	9	9	7	26	15	0
**Chromobacterium paludis**	44	45	48	79	135	81	6	7	36	9	10	4	6	20	21	0
**Serratia rubidaea**	75	91	43	59	137	47	4	9	29	3	6	6	9	29	3	0
**Porphyromonas gingivalis**	70	75	79	62	69	79	8	8	51	6	10	3	11	15	4	0
**Bordetella pertussis**	49	67	69	67	79	68	9	9	37	8	11	23	12	25	14	2
**Ralstonia mannitolilytica**	56	49	82	87	71	62	10	13	39	8	7	14	10	23	15	1
**Lachnospiraceae bacterium NSJ-29**	74	47	66	71	65	100	8	7	33	4	18	6	18	20	7	0
**Ottowia sp. oral taxon 894**	53	76	67	87	72	46	15	11	37	7	15	16	8	22	7	0
**Vibrio mimicus**	68	58	70	83	77	75	4	6	36	11	12	6	7	18	7	0
**Pseudomonas sp. 14181154**	15	15	11	15	9	7	1	0	8	7	4	0	44	257	144	1

Klebsiella sp. WP8-S18-ESBL-06	77	86	53	48	188	32	3	0	21	4	5	7	2	7	3	0
Victivallales bacterium CCUG 44730	60	66	64	34	78	53	24	20	25	20	9	20	28	24	11	0
Bosea sp. F3-2	44	40	36	46	31	37	13	39	71	33	30	24	20	50	20	1
Citrobacter werkmanii	72	47	77	59	100	88	4	4	24	7	7	10	4	20	11	0
Rhizobium leguminosarum	69	59	56	51	54	41	13	21	55	12	22	13	23	32	13	0
Variovorax boronicumulans	54	56	79	76	67	41	7	17	41	12	10	19	8	30	17	0
Aeromonas rivipollensis	85	59	99	92	41	38	5	21	40	1	2	6	21	19	4	0
Variovorax sp. PMC12	51	53	74	75	64	60	10	11	45	8	16	8	6	43	9	0
Sphingopyxis macrogoltabida	43	41	34	35	37	36	25	25	83	17	26	24	19	17	70	1
Pseudomonas sp. THAF187a	8	20	29	41	49	37	5	7	30	16	42	6	30	108	101	4
Sphingopyxis terrae	25	24	24	35	32	50	13	42	58	14	75	27	9	34	67	2
Cedecea neteri	71	63	42	65	88	107	9	6	30	6	11	10	8	8	6	0
Crenobacter cavernae	62	64	46	70	92	73	6	6	37	6	9	9	10	30	10	0
Dysgonomonas sp. HDW5B	80	82	78	46	91	83	1	5	30	2	3	8	7	6	7	0
Burkholderia cepacia	39	49	64	41	172	45	9	14	14	10	4	16	9	22	19	1
Megasphaera hexanoica	91	38	20	176	62	38	2	6	60	2	5	5	1	16	5	0
Chryseobacterium bernardetii	64	74	113	75	60	60	10	7	28	3	5	11	6	11	0	0
Pseudomonas sp. MSPm1	26	22	19	37	36	47	1	12	36	9	40	6	33	108	94	0
Hylemonella gracilis	52	58	68	77	78	50	7	21	35	4	14	14	13	28	4	0
Pseudomonas syringae group genomosp. 3	34	76	68	47	51	63	11	8	32	7	23	16	11	44	32	0
Lactobacillus paragasseri	64	145	56	21	116	64	16	4	11	1	7	8	0	8	1	0
Acinetobacter radioresistens	50	59	80	69	40	61	6	16	36	5	8	14	11	41	26	0
Rhodoferax ferrireducens	61	72	76	65	69	60	10	3	39	6	4	10	11	23	11	0
uncultured Bacteroides sp.	88	50	34	72	68	64	9	9	51	17	15	12	16	10	3	0
Pseudomonas sp. ABC1	16	19	22	43	35	60	3	7	32	7	33	7	23	132	75	1
Pseudomonas pohangensis	19	59	31	38	143	46	5	5	31	5	17	8	11	59	34	1
Pandoraea pnomenusa	46	73	82	58	42	40	7	15	44	12	12	9	9	42	19	0
Ramlibacter sp. H242	39	61	68	66	58	62	11	14	47	8	17	12	9	26	12	0
Chromobacterium sp. ATCC 53434	69	36	64	81	99	55	4	7	36	3	8	9	10	16	11	1
Yersinia ruckeri	67	68	44	137	59	84	1	4	18	2	5	3	3	10	4	0
Darwinula stevensoni	47	106	86	77	50	56	14	9	22	3	11	9	6	11	2	0
Candida albicans	51	56	60	39	18	56	28	28	56	14	29	23	17	26	4	0
Burkholderia gladioli	54	53	54	58	73	62	8	7	30	10	11	9	18	31	26	0
Proteus vulgaris	51	64	59	44	47	111	9	18	33	4	12	8	15	22	6	0
Chryseobacterium indoltheticum	68	98	98	72	53	41	8	6	18	5	12	6	9	6	1	0
Christensenella minuta	87	38	72	68	79	70	3	3	31	6	4	6	9	22	1	0
Aeromonas sp. CU5	66	67	44	90	91	72	4	6	18	5	12	6	4	7	7	0
Lactobacillus amylovorus	75	156	63	35	81	25	6	1	4	6	6	16	8	6	10	0
Aeromonas phage phiARM81mr	62	51	102	96	25	33	5	33	26	1	5	9	35	14	0	0
Alteromonas sp. RKMC-009	46	19	17	296	48	41	2	2	11	0	3	4	3	3	2	0
Pseudomonas sp. B11D7D	18	23	23	59	35	41	4	5	28	14	36	7	23	92	85	3
Sulfuritortus calidifontis	54	49	76	79	77	47	7	8	36	10	12	8	5	14	11	0
Aeromonas virus AhSzq1	1	38	325	11	4	0	3	56	1	0	1	8	44	0	0	0
Mitsuaria sp. 7	23	52	110	44	46	77	7	12	34	13	17	9	17	17	12	0
Pseudomonas sp. THAF42	12	17	14	41	31	31	6	5	20	9	37	5	32	118	111	1
Eubacterium limosum	91	62	52	62	52	66	9	7	23	8	8	12	15	14	8	0
Azospirillum brasilense	51	108	46	43	64	34	10	11	20	7	17	14	8	34	18	0
[Haemophilus] ducreyi	85	48	77	48	72	57	4	12	16	18	6	15	11	15	0	0
Curvibacter sp. AEP1-3	53	60	86	65	70	48	6	10	28	6	8	8	12	17	7	0
Klebsiella sp. WP7-S18-CRE-03	46	34	256	36	39	19	6	4	17	1	1	4	1	10	10	0
Streptococcus pasteurianus	86	26	39	71	83	48	8	8	55	7	9	6	15	13	9	0
Dysgonomonas sp. HDW5A	77	78	89	46	49	67	2	9	26	6	4	12	3	11	4	0
Sutterella faecalis	33	41	72	85	53	73	2	14	38	6	14	13	9	20	10	0
Xanthobacter autotrophicus	68	42	29	55	57	44	8	8	40	11	26	20	19	33	21	1
Saccharomyces cerevisiae	22	12	28	14	18	14	24	67	67	68	14	16	54	33	31	0
Jeongeupia sp. HS-3	73	52	60	87	65	54	10	6	23	9	3	8	7	20	4	0
Vibrio metschnikovii	66	63	75	84	47	99	2	4	17	4	8	2	4	4	2	0
Ethanoligenens harbinense	99	51	61	53	62	73	9	8	20	1	7	14	10	8	4	0
Streptococcus oralis	42	42	51	54	100	72	8	13	42	5	15	10	12	13	1	0
Streptococcus gordonii	63	25	38	30	75	89	0	24	51	15	8	17	3	28	13	0
Campylobacter coli	54	47	116	50	48	58	10	3	36	6	6	6	7	22	10	0
Thiobacillus sp.	121	55	38	66	61	44	10	9	17	9	3	13	11	12	8	0
Flavobacterium johnsoniae	54	78	96	63	41	52	5	4	27	12	9	14	3	14	4	0
Zobellella denitrificans	29	57	37	103	72	69	4	6	21	4	12	6	11	25	19	1
Flavobacterium columnare	63	88	83	51	49	49	9	11	32	1	8	7	9	9	5	0
Acinetobacter calcoaceticus	42	73	47	61	34	69	7	7	29	0	13	9	12	32	38	1
Acinetobacter soli	31	97	43	57	45	41	11	8	46	2	9	14	3	30	36	0
Pseudomonas sp. LPH1	12	24	31	60	30	39	5	8	29	10	25	6	18	101	75	0
Pseudomonas sp. HLS-6	21	16	101	51	45	23	13	12	42	6	12	11	22	67	30	0
Sphingomonas sp. IC081	26	78	64	48	53	38	18	22	36	8	10	14	14	30	12	0
Dickeya poaceiphila	48	32	87	83	59	107	2	3	25	3	4	3	5	7	1	0
Escherichia fergusonii	63	115	47	67	33	81	3	6	17	4	10	5	8	3	6	0
Vibrio harveyi	87	82	82	54	41	74	2	6	22	1	6	3	3	4	0	0
Hyphomicrobium denitrificans	75	26	16	17	25	21	20	16	101	35	23	22	11	45	14	0
Limosilactobacillus fermentum	71	64	45	30	83	90	1	8	21	9	19	8	5	4	9	0
Arcobacter anaerophilus	12	43	42	69	178	54	4	7	36	4	6	4	2	6	0	0
Kluyvera genomosp. 3	15	26	37	15	68	203	4	2	14	4	65	2	4	6	1	0
Rhodoferax antarcticus	43	59	75	60	70	40	11	7	39	6	8	9	6	23	9	0
Citrobacter sp. RHBSTW-00821	7	53	13	192	7	169	0	3	5	1	4	1	1	4	2	0
Citrobacter sp. RHBSTW-00696	20	19	17	34	266	58	6	1	9	4	6	3	2	12	2	0
Lachnospiraceae bacterium KM106-2	64	43	63	53	69	73	14	7	21	10	8	2	5	19	5	0
Aquitalea denitrificans	52	35	54	70	75	51	6	12	39	6	12	6	11	17	10	0
Moraxella osloensis	92	89	35	46	49	45	10	11	27	5	10	15	9	8	4	0
Pectobacterium brasiliense	83	125	37	43	44	39	6	2	35	3	7	8	6	11	5	0
Streptococcus lutetiensis	40	63	61	53	74	53	13	19	29	7	4	13	6	15	4	0
Draconibacterium orientale	60	68	94	50	64	62	5	7	22	2	6	4	3	4	1	0
Atlantibacter hermannii	29	44	21	53	141	74	6	3	31	8	10	2	7	12	11	0
Bacteroides graminisolvens	72	80	47	49	48	96	3	8	12	1	14	8	0	11	2	0
Variovorax sp. PAMC26660	43	50	54	60	58	52	6	9	30	9	21	17	4	20	18	0

Paucibacter sp. KCTC 42545	35	61	67	51	54	59	10	8	43	5	12	11	10	13	12	0
Cloacibacillus porcorum	99	38	63	42	78	62	8	5	12	4	7	4	10	12	4	2
Dehalobacter sp. CF	77	83	70	38	69	47	9	6	20	3	9	1	4	10	4	0
Vibrio rumoiensis	76	27	103	38	22	54	13	11	29	18	16	11	12	14	6	0
Klebsiella sp. RHBSTW-00464	62	48	11	83	179	40	1	0	10	7	3	0	1	2	2	0
Enterobacteriaceae bacterium	43	28	33	215	23	36	2	8	18	6	9	8	8	7	5	0
Listeria monocytogenes	88	79	86	40	40	67	10	2	10	4	8	5	4	3	2	0
Shewanella khirikhana	38	68	58	85	84	75	1	6	8	3	2	4	5	10	1	0
Methanothrix soehngenii	385	31	8	11	3	6	0	0	3	0	0	0	0	0	0	0
Flavobacterium psychrophilum	75	71	75	58	43	46	8	7	19	4	6	5	6	13	10	0
Megasphaera stantonii	70	39	28	77	77	67	6	6	35	4	5	6	6	13	7	0
Klebsiella sp. WP7-S18-ESBL-04	18	36	13	25	220	28	3	3	18	8	18	5	6	18	27	0
Cupriavidus pauculus	50	43	46	62	68	35	7	9	34	3	15	15	14	27	17	0
Achromobacter sp. AONIH1	66	60	72	49	49	47	4	12	28	3	7	11	11	18	7	0
Acinetobacter gyllenbergii	27	49	52	59	26	49	5	10	23	5	18	13	9	46	52	0
Acinetobacter sp. NCu2D-2	24	35	128	51	49	25	11	25	18	3	5	8	18	27	16	0
Chryseobacterium gleum	45	79	80	44	66	41	5	12	30	6	7	6	6	14	1	0
Bosea sp. AS-1	30	44	35	33	43	25	18	31	51	27	23	21	18	31	12	0
Pantoea dispersa	58	63	52	123	57	47	5	0	10	5	4	0	4	12	0	0
Serpentinomonas mccroryi	50	56	63	60	63	43	5	13	25	11	10	10	6	16	8	1
Escherichia albertii	29	95	26	75	74	71	7	4	22	5	8	8	0	9	6	0
Aeromonas virus AhSzw1	1	42	310	6	1	0	4	38	3	0	1	3	27	3	0	0
Lachnospiraceae bacterium NSJ-38	73	37	43	57	63	67	5	6	34	11	12	5	10	11	4	0
Erysipelotrichaceae bacterium GAM147	69	50	54	71	54	59	2	10	25	9	8	2	6	15	3	0
Methylibium sp. Pch-M	35	48	42	30	62	42	10	15	45	14	17	11	12	38	16	0
Aeromonas allosaccharophila	32	146	35	53	41	36	5	9	27	3	2	9	20	13	6	0
Ruminococcaceae bacterium BL-6	89	43	69	41	72	62	4	5	14	3	5	6	4	15	4	0
Mycolicibacterium mageritense	33	11	30	28	13	25	18	22	70	37	18	42	23	49	17	0
Pseudomonas sihuiensis	18	15	22	64	39	38	13	5	25	8	17	7	21	70	71	1
Proteiniphilum saccharofermentans	62	63	56	43	62	62	9	7	22	1	12	6	1	23	4	0
Pseudomonas alcaliphila	29	29	17	59	39	33	2	3	31	6	28	5	21	77	53	1
Paracoccus jeotgali	39	42	33	45	29	39	18	14	44	17	23	21	17	30	21	0
Brevundimonas diminuta	45	34	55	41	43	33	6	24	64	9	14	13	14	25	9	0
Paracoccus sp. AK26	46	40	32	37	39	47	9	17	46	15	14	18	12	34	22	0
Serpentinomonas raichei	39	37	57	53	76	58	6	6	27	6	12	5	10	19	14	0
Flavobacterium indicum	37	62	97	56	32	59	9	13	25	4	4	7	8	5	7	0
Sinorhizobium fredii	32	57	39	38	51	73	5	13	34	14	11	11	8	21	18	0
Variovorax sp. 38R	25	54	58	60	58	44	6	17	40	10	5	10	13	20	5	0
Rheinheimera sp. F8	15	30	39	69	141	72	4	8	22	5	5	2	4	8	1	0
Aeromonas encheleia	66	49	52	63	47	43	6	5	32	7	8	6	5	21	14	0
Catenovulum sediminis	8	146	3	49	204	4	1	1	1	1	1	0	1	2	1	0
Magnetospirillum gryphiswaldense	74	44	49	67	74	51	2	4	20	0	3	10	6	14	4	0
Vibrio navarrensis	54	59	54	51	60	54	10	12	21	4	11	8	8	10	6	0
Pseudomonas sp. DY-1	8	10	15	26	37	38	2	4	25	12	23	8	22	90	101	1
Pseudomonas parafulva	5	12	14	30	27	25	5	2	18	2	105	2	23	97	54	1
Cyprideis torosa	63	87	62	50	40	48	12	4	18	7	6	9	4	5	6	0
Acinetobacter shaoyimingii	41	70	103	45	20	35	2	13	18	5	8	9	15	18	18	1
Pseudomonas sp. phDV1	33	36	33	50	36	23	7	7	32	7	13	11	19	61	52	1
Tannerella forsythia	48	72	66	50	57	48	11	9	22	6	10	5	5	9	1	0
Thermomonas brevis	50	32	42	60	37	27	9	19	46	17	20	13	22	17	7	0
Limosilactobacillus reuteri	146	130	29	15	26	12	19	0	11	0	2	20	0	7	0	0
Achromobacter denitrificans	48	38	54	43	53	47	8	14	34	6	9	11	14	19	18	0
Gammaproteobacteria bacterium	53	66	53	61	57	43	2	4	19	3	8	1	10	23	11	1
Methylibium petroleiphilum	37	43	36	41	58	39	7	12	51	9	21	13	3	25	20	0
Myroides fluvii	51	39	40	94	79	38	9	9	22	2	3	7	10	9	2	0
Geobacter lovleyi	36	31	59	53	65	96	2	5	26	11	4	8	2	14	2	0
Burkholderia pseudomallei	56	40	39	46	64	48	7	7	23	4	13	7	16	25	17	0
Pantoea agglomerans	36	117	34	83	39	40	6	8	21	2	7	4	7	6	2	0
Dickeya dadantii	118	78	102	17	56	14	2	3	5	0	1	2	3	9	0	1
Kosakonia radicincitans	62	73	41	49	57	59	7	1	22	3	6	10	4	10	6	0
Notodromas monacha	29	50	93	34	54	27	1	11	14	3	4	10	15	26	36	1
Herbaspirillum seropedicae	48	31	52	57	67	34	6	8	30	7	12	10	12	23	9	1
Burkholderiales bacterium YL45	43	17	25	60	101	45	2	5	27	28	2	1	8	22	21	0
Methanobrevibacter smithii	99	29	34	43	83	56	2	8	20	2	10	2	4	14	0	0
Chryseobacterium sp. 7-3A	42	56	94	66	41	49	7	6	17	5	9	3	4	6	0	0
Paracoccus liaowanqingii	35	32	31	40	34	35	9	28	36	13	22	24	14	31	21	0
Aeromonas dhakensis	32	39	48	69	63	62	6	4	41	3	3	6	3	16	8	0
Shewanella decolorationis	29	19	69	64	113	8	13	1	40	3	27	8	4	2	3	0
Aeromonas phage LAh_7	24	76	65	64	34	71	14	16	14	0	3	10	2	10	0	0
Acinetobacter equi	19	21	181	42	25	10	2	13	31	6	2	4	12	19	14	2
Pseudomonas sp. VLB120	1	2	4	14	14	15	0	0	6	11	41	0	39	172	83	1
Chitinibacter sp. 2T18	35	38	44	31	50	53	15	15	25	19	15	13	12	24	13	0
Desulfitobacterium hafniense	79	55	54	48	54	47	3	5	12	1	8	12	6	12	5	0
Bradyrhizobium diazoefficiens	58	25	21	32	82	28	9	16	39	5	13	17	13	31	11	1
Streptococcus anginosus	54	67	50	34	61	56	10	7	28	5	3	3	9	12	2	0
Fusobacterium nucleatum	42	65	43	49	42	67	4	3	43	2	13	6	7	13	2	0
Ochrobactrum sp. MT180101	48	29	29	42	36	41	9	18	36	17	22	16	14	21	22	0
Cupriavidus gilardii	37	46	56	41	61	38	8	11	19	8	13	8	5	43	6	0
Blautia sp. LZLJ-3	67	46	45	42	47	64	3	2	38	3	11	6	7	15	3	0
uncultured bacterium 3e18	51	32	51	78	54	41	3	10	36	2	8	7	7	19	0	0
Sphingopyxis granuli	30	43	24	32	36	18	11	13	63	8	23	21	6	29	42	0
Grimontia hollisae	48	44	72	23	45	71	5	15	37	4	8	7	6	10	1	0
Curvibacter putative symbiont of Hydra magnipapil	68	50	59	59	50	37	4	6	26	5	2	8	4	11	5	0
Kluyvera intermedia	35	68	45	45	94	44	5	7	19	4	8	1	2	11	6	0
Castellaniella defragrans	25	35	46	44	49	44	5	47	30	5	10	22	15	11	6	0
Pseudomonas sediminis	12	13	18	47	45	28	6	8	27	6	25	7	18	63	71	0
Pectobacterium versatile	8	123	36	99	36	52	2	6	9	3	5	1	3	6	4	0
Haematobacter massiliensis	47	34	21	32	31	31	11	24	36	23	17	16	15	30	24	0
Acinetobacter wanghuae	17	50	124	41	23	24	4	27	28	1	4	9	5	21	11	2

Zoogloeaceae bacteirum Par-f-2	47	61	51	58	58	44	5	2	20	5	1	9	5	16	8	0
Variovorax sp. PAMC 28711	40	50	49	47	69	34	2	7	31	7	7	9	10	20	8	0
Elizabethkingia sp. M8	39	53	89	63	48	50	4	15	9	2	1	3	6	6	2	0
Defluviimonas alba	40	39	29	33	31	49	10	11	43	13	22	23	13	20	12	1
Ruminococcus sp. JE7A12	68	58	40	35	42	68	5	6	25	5	6	9	9	7	4	0
Elizabethkingia miricola	34	60	49	77	49	50	9	7	20	4	4	11	2	8	3	0
Propionibacterium freudenreichii	24	29	48	21	65	47	15	13	34	23	7	13	11	29	8	0
[Polyangium] brachysporum	39	43	58	36	52	45	11	12	27	8	8	9	10	15	13	0
Vibrio campbellii	18	73	62	44	92	55	5	7	9	4	4	3	2	5	3	0
uncultured bacterium 2M03	47	36	45	44	58	65	1	3	19	16	7	8	7	19	10	0
Photobacterium damselae	62	42	59	37	61	45	6	7	32	4	5	8	4	8	3	0
Bradyrhizobium sp. CCBAU 53421	16	17	3	13	9	3	26	37	49	27	45	40	28	40	28	0
Enterobacteriaceae bacterium Kacie_13	34	31	56	43	104	62	2	1	22	10	0	4	2	7	1	0
Kosakonia cowanii	57	35	38	78	49	29	2	5	45	5	6	4	0	18	7	0
Casimicrobium huifangae	32	52	48	68	66	38	6	4	22	5	6	7	5	14	5	0
Acinetobacter sp. MYb10	30	42	62	42	26	23	10	16	15	3	9	8	14	46	31	1
Yersinia pseudotuberculosis	16	55	5	70	158	47	2	0	6	2	5	1	2	4	4	0
Bacteroidetes bacterium	59	56	69	39	70	24	8	8	27	3	3	4	2	4	0	0
Cronobacter sakazakii	84	30	50	43	63	38	1	5	24	2	6	3	8	14	4	0
Cupriavidus oxalaticus	39	40	47	39	51	39	4	7	25	8	17	3	12	24	20	0
Enterobacter bugandensis	35	24	16	38	126	42	1	4	13	30	7	4	7	16	12	0
Citrobacter sp. RHBSTW-00599	58	54	28	38	92	44	3	7	12	2	16	7	3	6	4	0
Lysobacter enzymogenes	45	18	43	36	37	41	7	11	38	12	16	11	11	31	15	0
Pasteurella multocida	112	21	37	66	28	63	1	2	11	4	7	7	0	10	2	0
Weeksella virosa	35	40	82	76	39	21	8	11	17	3	13	4	7	12	3	0
Variovorax sp. RA8	27	49	48	47	61	28	11	9	33	9	9	7	8	15	10	0
Lactobacillus gasseri	52	82	80	22	51	28	7	7	13	1	3	8	6	2	7	0
Thiobacillus denitrificans	52	31	54	49	51	42	5	6	30	1	3	9	4	15	16	0
Rhodobacter sp. LPB0142	36	36	20	29	29	32	15	17	44	12	14	17	5	36	24	2
Chryseobacterium carnipullorum	56	52	77	53	39	37	3	3	15	1	9	7	3	8	1	0
Acinetobacter lanii	19	37	74	48	21	21	10	18	23	1	9	13	15	38	17	0
Pseudomonas sp. BIOMIG1BAC	19	25	22	40	58	29	8	9	25	15	14	14	20	42	23	0
Pantoea ananatis	43	67	28	38	60	73	5	3	18	5	10	0	3	5	3	0
Pectobacterium parmentieri	73	37	34	45	48	71	4	2	21	3	7	1	2	6	5	0
Pectobacterium polaris	65	45	36	24	130	30	0	1	9	3	6	2	5	2	1	0
Rhodobacter blasticus	45	29	36	22	41	35	3	14	47	11	13	22	7	19	13	1
Roseateles depolymerans	25	41	39	43	46	40	5	9	32	5	9	18	10	27	8	0
Turicibacter sanguinis	21	35	45	17	34	41	6	24	34	14	25	10	18	21	12	0
Vibrio genomosp. F6	69	44	52	54	46	42	6	2	16	1	5	0	4	11	4	0
Elizabethkingia bruuniana	47	46	64	55	53	33	6	6	19	3	8	6	2	6	2	0
Proteus sp. ZN5	1	23	316	5	4	3	1	1	1	0	0	0	1	0	0	0
Chryseobacterium carnis	36	44	76	62	41	33	3	8	19	6	1	7	6	8	5	0
Achromobacter spanius	32	39	43	53	51	47	2	17	21	3	7	6	10	10	14	0
Streptococcus vestibularis	58	36	17	11	79	85	6	5	17	11	5	3	5	4	12	0
Paracoccus sp. Arc7-R13	38	43	22	28	36	32	19	5	29	15	13	18	11	22	22	0
Achromobacter insolitus	37	42	47	59	48	30	4	11	25	5	9	8	6	16	6	0
Variovorax sp. PBS-H4	29	41	37	44	39	37	5	7	35	10	12	14	10	16	17	0
Delftia lacustris	26	40	44	47	48	38	5	9	24	4	9	6	7	25	20	0
Hydrogenophaga sp. PAMC20947	24	44	62	60	42	33	2	7	20	4	9	12	9	20	4	0
Alloprevotella sp. E39	45	44	38	45	35	59	4	12	26	2	11	2	11	9	8	0
Pseudomonas koreensis	24	18	35	42	23	26	5	4	11	7	20	9	26	64	37	0
Citrobacter sp. ABFQG	347	0	0	0	0	0	0	2	0	0	0	1	0	0	0	0
Ignavibacteria bacterium	62	64	52	55	37	25	6	8	18	2	4	6	3	8	0	0
Erwinia sp. J780	73	40	18	41	73	45	6	7	15	4	2	9	1	14	1	0
Naegleria gruberi	5	0	5	5	11	11	3	2	4	1	7	5	7	110	165	7
Eubacterium callanderi	71	32	39	29	44	49	6	7	15	8	5	9	11	16	6	0
Inhella inkyongensis	33	40	43	50	43	42	6	9	23	5	10	12	8	19	4	0
Rahnella sp. ERMR1:05	30	128	29	33	64	29	1	0	15	2	2	3	3	5	1	0
Aeromonas sp. CA23	36	37	47	34	57	70	6	4	17	1	10	3	5	12	4	0
Herbaspirillum rubrisubalbicans	29	49	54	45	58	33	1	6	22	4	12	7	4	7	12	0
Ruminococcus albus	25	75	36	47	37	80	2	3	13	1	10	2	4	6	2	0
Acinetobacter guillouiae	14	42	60	27	22	22	6	6	18	4	2	16	14	58	32	0
Tatumella citrea	52	39	42	47	59	51	6	4	14	3	6	4	3	11	1	0
uncultured bacterium EB1	42	30	30	53	49	73	4	4	26	1	9	0	2	16	3	0
Hydrogenophaga crassostreae	32	42	47	64	35	48	4	4	15	2	9	9	7	17	6	0
Bradyrhizobium sp. SK17	29	15	5	15	7	12	19	36	35	29	30	31	22	37	17	2
Klebsiella huaxiensis	60	22	46	50	29	64	3	6	17	3	6	3	24	5	2	0
Buttiauxella sp. 3AFRM03	40	69	44	57	30	54	6	1	12	4	5	5	2	8	3	0
Tessaracoccus flavus	36	40	36	26	47	33	9	8	36	13	22	13	6	7	8	0
Porphyromonas cangingivalis	49	49	58	42	40	44	7	6	21	3	5	4	4	6	0	0
Clostridium sp. SY8519	37	28	66	36	40	54	6	3	24	3	1	10	2	22	6	0
Hafnia paralvei	39	101	38	31	50	24	6	4	8	6	5	9	1	4	9	1
Rouxiella badensis	59	28	31	90	66	22	2	3	8	2	6	4	7	6	1	0
Paracoccus sanguinis	33	23	26	21	33	23	10	10	36	12	9	17	23	35	23	1
Muribaculum sp. TLL-A4	51	36	41	43	46	51	5	5	23	6	8	3	2	10	3	0
Malaciobacter halophilus	15	33	40	79	71	30	4	1	32	1	5	2	4	8	7	0
Citrobacter youngae	65	80	18	27	65	35	7	3	9	3	5	1	1	10	2	0
Chryseobacterium balustinum	37	38	78	41	28	40	5	6	24	4	11	3	8	7	1	0
Zymomonas mobilis	63	62	41	13	111	27	0	0	3	1	3	2	1	3	0	0
Chryseobacterium nakagawai	47	59	62	40	33	28	7	5	28	2	4	2	4	6	3	0
Vibrio scophthalmi	39	66	54	46	62	26	4	2	9	3	5	3	0	3	8	0
Citrobacter sp. RHBSTW-00887	16	83	3	53	69	76	4	2	6	0	4	2	3	8	1	0
Dehalobacterium formicoaceticum	61	48	46	28	58	45	5	2	13	5	5	4	4	3	2	0
Flavobacterium sp. P2-65	34	48	78	37	24	48	5	10	15	5	7	4	8	6	0	0
Acinetobacter sp. ACNIH1	24	34	61	52	41	30	5	11	25	0	2	16	8	13	7	0
Pseudomonas mandelii	13	24	21	53	16	48	7	2	31	7	9	1	18	48	31	0
Polaromonas sp. SP1	44	47	50	52	40	35	2	6	14	6	5	6	10	8	3	0
Anaerocolumna cellulosilytica	44	44	49	39	38	63	1	6	8	0	2	7	5	11	11	0
Methanospirillum hungatei	235	36	11	5	17	9	1	0	9	0	1	3	0	0	0	0

Staphylococcus aureus	52	47	37	39	49	47	1	3	12	4	7	11	4	13	1	0
Dickeya fangzhongdai	39	51	39	48	19	66	10	2	13	8	4	6	5	11	6	0
Xanthomonas oryzae	35	28	37	34	56	26	3	7	30	3	9	12	7	23	16	1
Variovorax sp. PBL-H6	29	43	39	38	46	30	10	11	25	5	7	9	5	23	6	0
Paracoccus contaminans	35	32	17	17	28	33	11	16	46	15	22	14	5	21	12	0
Bordetella bronchiseptica	29	23	45	54	41	30	6	10	22	7	13	16	3	21	4	0
Shewanella algae	18	86	40	37	22	71	4	2	17	5	3	4	8	6	1	0
crAssphage cr10_1	80	51	30	39	20	33	5	6	21	2	10	6	8	6	4	0
Mucinivorans hirudinis	56	52	49	25	45	43	4	5	12	1	7	4	6	8	4	0
Pseudomonas brassicacearum	19	13	15	16	28	43	2	2	20	4	15	11	20	61	51	0
Kosakonia arachidis	42	43	38	43	37	76	3	2	14	0	6	4	5	4	2	0
Clostridium kluyveri	60	63	32	37	40	35	1	5	18	3	3	10	3	8	0	0
Vibrio alginolyticus	57	24	37	34	52	51	1	3	8	7	9	3	7	18	7	0
Rheinheimera sp. LHK132	52	23	42	60	18	72	3	3	14	0	3	9	4	11	4	0
Sulfurivermis fontis	51	36	43	39	51	28	1	10	19	4	10	7	7	6	6	0
Xenorhabdus nematophila	49	18	28	33	86	24	11	9	17	5	9	6	7	12	4	0
Noviherbaspirillum sp. UKPF54	35	43	42	52	34	30	10	7	20	3	4	8	9	13	8	0
Pseudoxanthomonas sp.	33	28	28	31	20	26	10	15	40	9	18	10	10	21	19	0
Brucella intermedia	24	38	17	33	33	35	6	6	67	8	11	9	2	16	13	0
Mycolicibacterium mucogenicum	57	14	20	16	20	18	20	26	47	11	11	10	8	29	9	0
Shewanella marisflavi	43	32	38	75	81	29	0	2	7	0	1	1	5	1	1	0
Syntrophobotulus glycolicus	54	42	42	31	45	53	3	4	11	3	3	8	1	9	6	0
Polaromonas sp. Pch-P	28	37	47	46	41	26	3	9	32	3	7	8	4	17	7	0
Enterobacteriaceae bacterium ENNIH1	14	77	16	31	31	101	1	2	30	3	2	2	2	3	0	0
Methanosarcina barkeri	78	8	11	16	22	9	17	10	45	14	21	19	9	28	7	0
Christensenella sp. Marseille-P3954	59	34	38	26	53	61	4	2	15	0	3	5	4	7	3	0
Dickeya dianthicola	32	20	23	55	69	61	4	5	18	3	6	3	3	8	3	0
Sphingobacterium sp. PM2-P1-29	43	36	64	68	25	33	6	4	12	3	7	6	0	2	3	0
Geobacter sulfurreducens	38	31	31	29	51	47	6	3	4	8	5	42	2	7	6	2
Citrobacter farmeri	16	79	52	30	36	35	2	3	19	2	1	7	9	15	6	0
Pseudomonas sp. CCOS 191	9	6	13	20	11	10	1	5	8	4	11	1	60	103	50	0
crAssphage cr53_1	50	10	30	49	38	45	0	1	26	8	16	4	2	28	4	0
Pragia sp. CF-458	21	42	41	35	94	37	1	5	17	2	4	3	2	5	1	0
Acinetobacter sp. TGL-Y2	15	49	72	25	29	28	1	8	17	0	5	5	5	24	25	0
uncultured bacterium fosmid pJB69A5	5	16	117	111	43	3	3	2	1	0	2	1	1	1	2	0
Rhodobacter capsulatus	35	27	21	32	31	33	8	10	23	11	19	12	6	15	24	0
Yersinia rohdei	30	85	36	28	82	12	2	4	5	1	3	5	5	8	0	0
Kosakonia sacchari	46	45	28	40	62	30	5	7	14	0	0	5	4	15	4	0
Aliivibrio salmonicida	25	30	53	51	32	38	6	4	22	4	13	8	7	9	3	0
Tannerella sp. oral taxon HOT-286	56	35	47	28	42	38	3	4	16	4	5	6	4	10	4	0
Herbaspirillum robiniae	25	32	38	42	45	29	3	4	24	6	12	4	8	15	15	0
Enterococcus casseliflavus	57	65	72	15	21	23	4	7	13	3	4	3	9	4	1	0
Acidipropionibacterium acidipropionici	29	19	24	60	14	18	8	9	46	6	11	12	14	20	10	1
Pseudomonas alkylphenolica	14	18	18	24	26	22	0	4	15	6	12	4	24	53	58	3
Burkholderia glumae	36	26	32	35	46	24	5	7	23	6	5	16	7	18	14	0
Pragia fontium	65	47	32	44	36	22	1	3	24	0	5	6	2	5	7	0
crAssphage cr115_1	58	26	28	34	69	37	4	1	23	2	6	2	2	7	0	0
Desulfobulbus oligotrophicus	50	18	26	23	56	38	8	14	14	6	11	4	5	19	6	0
Sulfurimonas sp. CVO	19	47	38	47	51	53	6	4	16	1	3	6	3	2	1	0
Elizabethkingia meningoseptica	31	44	80	20	32	29	2	6	31	2	1	6	4	6	2	0
uncultured Sulfurospirillum sp.	24	51	47	37	12	26	10	4	10	0	6	56	7	5	1	0
Clostridium saccharoperbutylacetonicum	49	62	19	31	27	60	7	6	11	0	10	4	4	1	4	0
Aminobacter sp. MSH1	44	31	30	20	12	20	17	12	40	6	19	7	8	18	11	0
Pantoea sp. MSR2	31	36	51	54	65	8	8	3	12	3	5	4	1	13	1	0
Sinimarinibacterium sp. NLF-5-8	20	35	40	42	46	27	8	6	16	5	13	5	11	10	10	0
Pandoraea thiooxydans	68	53	24	28	35	28	2	3	10	2	6	8	0	13	12	1
Mesorhizobium sp. 8	44	25	31	24	17	15	11	13	48	9	17	9	11	16	3	0
Chryseobacterium sp. SNU WT5	28	34	95	33	33	25	5	6	13	0	5	2	2	10	2	0
Raoultella terrigena	28	57	31	38	63	30	2	3	17	3	1	1	3	13	3	0
Pseudomonas orientalis	24	20	22	39	29	28	4	0	12	7	16	9	11	42	29	1
Acinetobacter variabilis	20	41	41	32	15	63	12	10	20	2	3	10	3	7	14	0
Serratia liquefaciens	39	27	69	31	32	35	2	7	18	3	6	4	3	15	1	0
Massilia putida	36	24	40	40	44	31	4	6	20	7	6	8	7	12	6	1
Dickeya solani	20	34	12	59	74	37	3	1	17	0	2	10	10	7	6	0
Proteus cibarius	20	14	81	43	39	29	1	7	11	2	6	6	17	11	4	0
Pantoea sp. SO10	47	25	30	48	54	36	4	3	15	4	4	6	3	9	1	1
Streptococcus australis	37	36	42	26	37	50	4	6	14	8	10	6	10	1	2	0
Burkholderia ubonensis	39	32	54	35	33	28	4	2	22	2	5	3	10	15	4	0
Paracoccus sp. MC1862	23	19	17	30	29	29	5	13	30	13	14	13	9	24	20	0
Serratia grimesii	61	32	19	60	40	37	2	4	8	2	3	4	1	10	4	0
Vibrio coralliilyticus	57	37	23	47	49	21	3	5	22	0	4	7	2	5	5	0
Blattabacterium cuenoti	44	47	49	28	39	44	2	3	11	0	3	2	8	5	1	1
Lactobacillus crispatus	114	24	20	18	44	26	2	2	16	3	9	4	1	0	3	0
Muribaculum intestinale	43	30	35	29	35	51	4	6	26	3	8	4	2	5	5	0
Pannonibacter phragmitetus	28	21	16	23	41	33	7	10	36	10	13	10	8	21	9	0
Duncaniella dubosii	33	49	35	33	35	34	3	7	18	3	9	4	11	9	2	0
Brenneria nigrifluens	49	39	18	21	53	72	4	4	2	1	3	2	0	14	2	0
Sorangium cellulosum	42	35	27	28	32	35	6	6	23	7	10	6	13	9	5	0
Pluralibacter gergoviae	19	23	39	36	23	55	2	0	37	11	6	15	5	8	5	0
Lautropia mirabilis	21	31	25	29	26	34	8	10	35	9	6	14	3	20	12	0
Klebsiella sp. WP4-W18-ESBL-05	125	26	11	5	63	34	0	3	2	1	1	4	2	3	2	0
Exiguobacterium profundum	37	20	33	24	27	19	3	16	38	7	12	9	7	21	9	0
Bordetella parapertussis	38	29	36	40	46	26	5	3	19	5	3	5	5	14	7	0
Duganella sp. GN2-R2	37	26	37	32	36	38	7	9	21	3	5	4	8	11	7	0
Chryseobacterium sp. 3008163	28	41	71	35	34	24	3	4	20	2	3	4	3	8	1	0
Chlamydia trachomatis	27	31	37	38	37	23	8	8	23	5	10	8	13	9	3	0
Prolixibacteraceae bacterium WC007	36	47	36	44	40	38	1	4	14	3	5	2	3	5	1	0
Pseudomonas soli	5	7	5	7	8	7	1	5	7	5	16	5	47	71	83	0
Elizabethkingia sp. JS20170427COW	38	45	56	43	21	32	4	5	12	1	6	3	4	8	0	0

**Chania multitudinisentens**	36	58	15	50	47	36	6	1	9	2	4	4	4	6	0	0
**Streptococcus mitis**	33	37	36	32	30	34	7	5	22	6	20	3	3	7	3	0
**Xanthomonas translucens**	21	25	25	30	29	29	7	8	34	7	18	5	6	22	12	0
**Vibrio sp. SM1977**	62	24	15	30	76	42	3	3	12	2	2	0	1	5	0	0
**Variovorax sp. SRS16**	31	35	34	36	31	23	5	9	17	1	12	4	12	17	10	0
**Brevundimonas naejangsanensis**	33	21	28	29	17	34	11	14	24	8	10	11	7	17	12	0
**Citrobacter sp. RHB36-C18**	14	7	9	7	202	13	0	4	7	0	5	1	0	4	3	0
**Pseudomonas sp. SK**	4	3	11	10	13	14	1	2	13	5	15	3	25	96	60	1
**Vibrio europaeus**	128	21	9	49	49	10	0	0	4	0	0	0	1	3	1	0
**Vibrio natriegens**	29	22	29	35	75	35	5	0	15	2	0	6	7	14	1	0
**Acinetobacter sp. 10FS3-1**	15	44	57	26	22	20	18	6	25	2	1	7	5	16	11	0
**Citrobacter sp. S39**	72	17	7	11	127	19	6	2	5	2	0	0	1	4	1	0
**Bordetella trematum**	43	27	37	23	42	36	9	6	13	2	7	6	1	8	13	0
**Dickeya aquatica**	31	82	25	54	15	28	0	2	12	6	1	8	3	2	4	0
**Thiomonas sp. CB2**	25	31	47	39	20	27	4	9	16	7	8	8	3	23	5	1
**Haemophilus influenzae**	18	22	25	29	57	60	2	2	15	4	7	10	9	8	4	0
**Pseudomonas sp. KU43P**	5	8	11	8	14	11	1	3	9	5	12	1	27	106	48	3
**Shimwellia blattae**	16	76	28	33	29	51	1	5	10	2	5	5	3	6	1	0
**Yersinia frederiksenii**	63	35	38	29	64	12	5	3	7	2	1	3	2	4	2	0
**[Clostridium] hylemonae**	48	26	33	36	33	39	6	7	8	6	8	4	3	7	6	0
**Caulobacter vibrioides**	34	25	39	36	22	37	5	7	16	6	5	4	11	12	10	0
**Chryseobacterium glaciei**	34	53	57	36	28	28	2	4	9	4	1	4	3	3	2	0
**Pseudomonas sp. CMR5c**	20	13	15	28	32	40	5	4	8	4	16	3	10	38	31	1
**Pseudomonas poae**	9	14	14	34	27	24	6	6	18	6	14	6	15	41	33	1
**Dickeya chrysanthemi**	43	82	38	24	8	28	3	2	11	2	9	5	2	5	5	0
**Anaerocolumna sp. CTTW**	30	43	33	28	39	32	7	4	8	0	6	6	6	8	17	0
**Lelliottia jeotgali**	29	72	16	50	53	22	3	3	6	1	3	4	2	2	1	0
**Pantoea stewartii**	23	32	14	58	67	49	2	1	10	1	4	1	3	1	1	0
**Citrobacter freundii complex sp. CFNIH4**	50	12	9	21	80	64	3	0	7	6	4	0	0	9	1	0
**Capnocytophaga canimorsus**	48	16	28	83	48	23	2	1	5	2	2	1	3	3	1	0
**Oceanisphaera profunda**	36	53	32	44	30	23	7	0	12	5	5	5	2	11	1	0
**Serratia symbiotica**	35	33	21	34	49	53	4	2	22	0	3	2	3	4	1	0
**Escherichia marmotae**	34	28	52	18	68	18	2	4	16	0	5	6	8	7	0	0
**Thermomonas sp. SY21**	27	22	17	23	19	18	7	11	42	6	16	12	7	31	8	0
**Pseudomonas synxantha**	17	8	21	27	22	25	4	5	15	5	20	6	18	37	34	2
**Enterobacter sp. 638**	66	21	19	10	82	32	6	1	6	5	6	4	2	4	1	0
**Tissierellia sp. JN-28**	39	28	34	38	49	40	9	1	10	3	4	7	2	1	0	0
**Pseudomonas fragi**	18	15	16	40	30	19	5	0	17	10	17	2	6	47	22	1
**Flavobacterium sediminis**	28	56	48	37	25	27	4	3	14	2	3	6	4	4	3	0
**Sulfuriferula plumbiphila**	27	40	30	35	37	28	3	8	15	3	5	11	3	16	3	0
**Burkholderia thailandensis**	23	53	46	30	27	27	3	4	17	5	0	4	5	7	13	0
**Yersinia massiliensis**	24	59	26	24	26	51	3	4	19	1	7	5	4	7	2	0
**Serratia proteamaculans**	32	89	44	17	13	28	1	4	9	4	4	4	3	8	0	0
**Pectinatus frisingensis**	30	29	19	47	39	42	2	6	23	2	5	6	2	4	4	0
**Massilia sp. WG5**	17	28	34	39	38	27	3	4	13	6	7	5	7	15	15	2
**Citrobacter sp. RHBSTW-00229**	38	26	14	54	73	29	1	4	4	1	6	4	2	3	0	0
**Variovorax sp. PBL-E5**	28	28	32	40	37	29	3	7	12	4	6	6	4	8	15	0
**Sym plasmid**	25	31	32	33	33	19	6	7	12	8	6	4	9	11	20	3
**Methylophaga frappieri**	7	48	33	34	64	66	0	2	2	0	1	1	0	0	1	0
**Chromobacterium sp. Rain0013**	21	29	22	48	46	31	4	1	15	2	9	2	3	12	12	1
**Cupriavidus nantongensis**	21	29	29	30	27	36	5	8	24	1	11	11	6	13	7	0
**Vibrio qinghaiensis**	1	17	6	16	4	208	0	0	5	1	0	0	0	0	0	0
**Sphingomonas wittichii**	27	21	10	7	13	16	6	21	29	11	9	13	8	47	18	1
**Rhodocyclaceae bacterium**	33	25	31	49	43	31	6	4	12	1	4	1	5	7	4	0
**Aliivibrio fischeri**	25	16	41	20	84	45	0	1	5	2	3	3	3	4	4	0
**Stenotrophomonas sp. CW117**	14	33	36	47	31	23	2	8	18	4	4	7	8	16	5	0
**Flavobacteriaceae bacterium**	100	21	27	35	24	18	1	3	10	1	3	7	3	1	1	0
**Lactobacillus mucosae**	27	55	34	15	61	34	2	3	0	9	2	4	5	4	0	0
**Mesorhizobium loti**	36	14	17	19	25	21	8	10	35	11	12	7	10	21	8	0
**Cupriavidus sp. USMAA2-4**	21	27	39	26	38	24	1	3	28	3	8	5	2	12	17	0
**Acinetobacter sp. LoGeW2-3**	17	25	62	27	30	17	2	8	22	1	6	8	5	16	8	0
**Selenomonas ruminantium**	33	29	28	33	48	30	1	1	19	5	6	4	4	6	6	0
**Rhodovulum sulfidophilum**	25	21	20	16	18	32	5	9	28	1	11	14	10	23	20	0
**Vibrio cincinnatiensis**	58	33	13	56	39	28	2	1	13	1	1	4	2	1	0	0
**Streptococcus agalactiae**	31	20	24	29	32	75	1	7	15	2	5	2	4	5	0	0
**Erwinia billingiae**	39	32	25	46	28	27	0	3	23	3	8	5	4	6	2	0
**Confluentimicrobium sp. EMB200-NS6**	33	33	11	20	21	13	8	10	31	11	10	15	10	16	9	0
**uncultured archaeon**	30	4	1	7	197	3	0	3	4	0	0	1	0	1	0	0
**Janthinobacterium agaricidamnosum**	22	34	26	32	41	30	3	6	18	5	5	2	6	15	6	0
**Salmonella phage SSU5**	40	21	14	42	53	51	3	1	7	2	7	0	1	5	3	0
**Rhodanobacter denitrificans**	35	55	16	17	13	15	6	5	21	7	10	5	10	23	12	0
**Vibrio spartinae**	31	83	26	16	31	34	3	1	9	1	3	3	4	4	1	0
**Labrys sp. KNU-23**	23	21	17	25	21	19	6	16	29	14	11	8	8	24	6	0
**Flavobacterium sp. I3-2**	65	56	22	20	26	26	2	3	15	1	0	6	2	2	1	0
**Massilia sp. UMI-21**	25	29	27	54	33	24	2	7	17	5	6	4	1	6	7	0
**Citrobacter freundii complex sp. CFNIH3**	22	28	27	30	58	36	1	4	14	3	10	2	4	4	4	0
**Collimonas fungivorans**	31	25	41	31	36	30	6	4	15	2	6	5	2	7	4	1
**Sphingopyxis fribergensis**	18	10	15	14	22	14	5	10	15	5	14	21	15	11	56	1
**Pantoea rwandensis**	17	75	49	25	18	26	2	4	10	4	2	8	1	5	0	0
**Edwardsiella tarda**	13	27	65	19	15	55	0	8	11	4	5	5	14	3	2	0
**Histophilus somni**	79	25	21	19	19	21	5	3	14	4	7	4	2	16	6	0
**Ornithobacterium rhinotracheale**	30	28	27	27	41	25	5	6	13	5	8	5	3	18	4	0
**Ralstonia insidiosa**	22	21	25	30	37	27	6	2	26	2	6	5	14	15	6	0
**Methylobacterium radiotolerans**	9	4	6	11	6	8	11	14	28	24	28	26	31	25	12	0
**Chryseobacterium sp. G0201**	37	28	55	34	24	28	1	5	13	1	5	3	3	5	0	0
**Chryseobacterium sp. 6424**	30	27	52	18	60	17	6	1	7	3	2	5	6	7	1	0
**Delftia sp. Cs1-4**	25	34	35	34	24	25	2	9	19	3	4	6	7	11	4	0
**Tessaracoccus lapidicaptus**	22	31	12	28	28	25	5	10	28	5	13	10	6	17	2	0
**Janthinobacterium sp. Marseille**	17	22	47	25	48	32	1	1	22	3	2	3	6	8	5	0

**Chryseobacterium jeonii**	44	36	31	39	24	17	2	6	21	2	4	6	6	3	0	0
**Vibrio sp. ZWAL4003**	36	57	21	38	12	61	1	4	4	1	4	0	0	1	1	0
**Paracoccus mutanolyticus**	29	23	13	15	18	23	9	14	27	13	13	7	8	20	9	0
**Collimonas arenae**	23	38	30	29	45	31	5	2	12	5	4	6	4	6	1	0
**Lactococcus lactis**	23	29	39	18	50	33	10	4	12	0	10	0	5	5	3	0
**Phytobacter ursingii**	17	53	50	33	10	39	3	1	15	2	7	1	2	7	1	0
**Tessaracoccus sp. T2.5-30**	16	31	23	24	24	19	14	9	31	9	7	8	10	12	4	0
**Brenneria goodwinii**	27	36	27	15	64	26	4	2	30	1	1	2	0	5	0	0
**Bacteroides stercoris**	26	21	35	38	12	37	2	7	22	3	13	6	8	10	0	0
**Bosea sp. RAC05**	23	19	21	22	23	12	13	13	39	9	11	5	9	17	4	0
**Labilibaculum antarcticum**	45	39	34	23	33	33	4	4	7	1	3	4	3	5	1	0
**Yersinia entomophaga**	23	49	16	31	54	24	2	4	13	1	1	3	2	10	6	0
**Streptococcus equinus**	17	16	31	14	20	103	3	0	15	1	8	0	1	8	2	0
**Mannheimia granulomatis**	62	18	48	12	40	25	1	2	18	2	2	5	0	2	1	0
**Anaerocolumna sp. CBA3638**	29	31	39	24	40	37	2	6	12	2	4	0	3	6	3	0
**Leclercia sp. W6**	19	40	73	48	24	8	3	0	13	0	3	1	0	5	1	0
**Pseudomonas sp. R2A2**	8	13	11	16	21	11	1	5	17	4	19	4	11	60	36	1
**Massilia oculi**	24	29	35	27	40	23	2	2	11	3	10	4	6	15	6	0
**Massilia albidiflava**	24	32	26	33	55	21	3	0	15	2	6	5	6	7	2	0
**Butyrivibrio proteoclasticus**	37	39	36	32	20	25	3	6	5	5	7	5	4	9	3	0
**uncultured bacterium 4L05**	32	12	36	32	29	42	2	0	23	2	6	2	2	11	5	0
**Thalassolituus oleivorans**	30	15	26	50	28	17	2	5	30	5	4	4	6	10	4	0
**Dokdonella koreensis**	29	14	22	21	12	27	3	13	27	8	9	8	8	25	10	0
**Mycolicibacter minnesotensis**	45	5	10	11	7	8	12	14	33	16	10	16	16	25	6	1
**Clostridium sp. BNL1100**	45	63	22	22	18	25	8	4	13	2	2	7	2	2	0	0
**Chitinimonas arctica**	32	29	30	31	23	41	6	1	16	3	1	3	2	12	5	0
**Chryseobacterium sp. NBC 122**	32	48	39	30	30	20	0	6	11	3	1	8	4	2	0	0
**Massilia umbonata**	27	26	23	33	35	22	5	4	14	2	10	9	5	14	5	0
**Streptococcus gallolyticus**	39	17	35	36	29	13	2	5	26	1	3	3	10	11	3	0
**Malaciobacter mytili**	36	42	29	32	22	41	6	4	10	1	3	3	1	3	0	0
**Desulfovibrio gigas**	22	24	51	20	42	18	2	17	11	2	6	0	8	7	3	0
**Proteus columbae**	14	38	50	19	25	48	1	4	18	0	6	0	5	5	0	0
**Mycolicibacterium aubagnense**	141	4	14	11	3	5	2	6	19	3	4	3	4	13	0	0
**Vibrio cidicii**	47	22	40	42	19	17	0	0	6	1	4	9	5	17	3	0
**Pigmentiphaga sp. H8**	26	23	31	27	20	23	1	7	23	6	5	7	5	16	12	0
**Serratia quinivorans**	14	55	36	43	27	25	3	0	12	2	0	1	4	8	2	0
**Clostridium saccharobutylicum**	44	31	27	27	19	37	1	6	12	6	6	5	5	2	2	0
**Schaalia odontolytica**	37	12	9	32	15	42	4	5	20	4	16	12	8	8	5	0
**Duganella sp. AF9R3**	31	20	29	26	39	32	1	4	9	4	5	7	2	12	8	0
**Bradyrhizobium sp. 6(2017)**	11	14	8	9	7	12	10	18	26	15	31	20	19	13	16	0
**Lactobacillus delbrueckii**	30	34	21	23	55	33	4	0	5	5	5	4	2	3	4	0
**Parvibaculum lavamentivorans**	28	28	28	42	21	24	5	4	18	2	6	5	2	8	6	0
**Candidatus Thiodictyon syntrophicum**	21	28	18	29	10	62	2	2	13	2	12	4	5	11	7	0
**Providencia heimbachae**	12	18	21	21	114	12	0	2	15	3	2	1	2	3	0	0
**Oligotropha carboxidovorans**	32	18	15	21	9	12	2	11	32	14	8	11	12	16	12	0
**Halomonas meridiana**	15	52	37	26	47	13	0	1	10	4	4	3	4	5	4	0
**Yersinia canariae**	15	72	34	32	15	25	1	2	16	1	4	2	0	5	1	0
**Pseudodesulfovibrio profundus**	45	25	16	26	45	39	4	0	6	2	7	3	0	4	2	0
**Enterobacter sp. RHBSTW-00994**	39	17	42	43	23	19	6	3	11	2	8	1	4	5	1	0
**Burkholderia contaminans**	30	21	28	22	30	43	6	4	21	4	0	4	1	8	2	0
**Lachnoclostridium phytofermentans**	26	27	35	32	26	27	3	4	18	1	3	6	8	6	2	0
**Delftia sp. HK171**	25	20	23	30	29	31	6	5	19	1	4	5	2	11	13	0
**Acinetobacter sp. FDAARGOS_724**	16	22	47	28	23	15	1	8	14	3	5	0	6	17	19	0
**Ruminococcaceae bacterium BL-4**	33	31	30	29	21	37	2	0	10	0	11	9	4	4	2	0
**Rhizobium daejeonense**	25	20	28	25	15	14	8	12	35	9	9	1	2	19	1	0
**Citrobacter sp. RHB25-C09**	25	41	31	61	17	26	3	0	5	1	2	3	1	5	2	0
**Cupriavidus campinensis**	28	21	25	33	24	22	3	4	20	1	4	6	7	21	3	0
**Mycolicibacter hiberniae**	21	6	7	15	12	9	12	18	26	19	16	25	12	21	3	0
**Pseudomonas sp. BIGb0427**	6	5	12	21	15	21	1	2	23	2	21	1	9	50	32	1
**Citrobacter pasteurii**	42	16	13	21	78	22	0	3	7	2	3	1	3	9	1	0
**Alkalitalea saponilacus**	33	39	25	29	34	30	4	2	7	0	3	4	4	6	1	0
**Vibrio nigripulchritudo**	30	52	16	30	25	37	3	2	7	2	4	4	6	2	1	0
**Massilia sp. NR 4-1**	25	21	33	30	35	26	2	6	12	4	2	5	6	8	6	0
**Dickeya paradisiaca**	23	23	38	30	33	33	1	3	9	1	5	4	3	13	2	0
**uncultured bacterium 22g15**	18	23	32	40	37	39	1	3	12	0	5	5	3	3	0	0
**Pseudomonas yamanorum**	12	15	16	24	15	66	3	1	14	8	6	3	10	19	9	0
**Butyrivibrio hungatei**	35	29	24	28	26	41	3	5	8	5	2	6	1	4	3	0
**Xenorhabdus bovienii**	20	46	16	32	19	35	5	2	21	0	4	8	3	9	0	0
**Bordetella genomosp. 13**	34	27	22	27	26	14	2	7	19	4	6	6	8	10	7	0
**Flavobacterium sangjuense**	32	39	30	23	20	30	8	4	9	2	4	4	7	4	3	0
**Bordetella petrii**	20	22	21	29	26	18	3	5	10	3	9	4	7	37	5	0
**Methylococcus sp. IM1**	19	18	26	37	24	27	2	6	21	5	4	6	8	11	5	0
**Dyella thiooxydans**	64	21	10	24	14	8	3	9	10	5	14	5	5	14	12	0
**Magnetospirillum magneticum**	27	34	38	31	21	22	3	9	10	0	8	3	2	9	1	0
**Erwinia gerundensis**	24	15	37	13	46	36	4	4	13	1	8	4	4	7	2	0
**Pseudomonas sp. StFLB209**	13	15	23	29	22	18	1	2	13	1	6	6	7	35	27	0
**Enterobacter cancerogenus**	38	26	44	19	39	21	4	2	10	0	2	1	4	5	2	0
**Mycolicibacter terrae**	32	12	10	16	9	8	7	18	35	13	11	17	7	12	10	0
**Lelliottia amnigena**	28	39	36	21	18	24	3	2	26	2	5	2	3	6	1	0
**Immundisolibacter cernigliae**	22	20	41	26	33	21	0	3	16	3	5	6	2	11	7	0
**Aggregatibacter actinomycetemcomitans**	19	14	18	33	86	18	4	1	10	4	2	1	1	4	1	0
**Pseudobutyrivibrio xylanivorans**	29	17	30	24	26	47	2	5	14	0	6	3	4	7	1	0
**Blautia coccoides**	26	23	28	24	26	35	6	8	18	2	3	2	5	5	4	0
**Lelliottia nimipressuralis**	44	33	19	22	25	35	2	5	17	0	2	3	2	1	4	0
**Saccharophagus degradans**	2	0	28	26	107	49	0	0	1	0	1	0	0	0	0	0
**Sphingomonas koreensis**	16	13	32	12	6	20	3	19	19	5	6	12	13	10	27	0
**Colwellia sp. 20A7**	132	11	7	5	3	12	1	1	32	1	1	3	1	2	0	0
**Gibbsiella quercinecans**	48	10	18	30	22	52	0	2	14	5	1	2	3	3	2	0
**Dehalobacter restrictus**	38	36	38	23	32	19	2	1	10	0	2	2	4	5	0	0

Magnetospirillum sp. ME-1	34	20	31	33	29	18	5	5	7	4	4	3	7	5	7	0
Azospirillum lipoferum	33	28	17	26	22	18	7	3	13	6	7	5	3	12	12	0
Desulfovibrio alaskensis	30	24	41	13	38	29	3	6	9	1	3	7	3	2	3	0
Acidihalobacter prosperus	11	36	8	65	11	68	0	1	1	2	0	1	0	4	4	0
Bacteriovorax stolpii	39	33	29	19	28	13	8	2	11	2	8	4	6	1	8	0
Enterococcus cecorum	35	21	52	22	23	28	1	6	11	1	2	3	0	4	2	0
Massilia lutea	32	18	28	34	32	25	3	2	8	3	7	8	3	4	4	0
Draconibacterium sp. M1	29	30	45	21	36	28	1	1	8	2	3	1	2	3	1	0
Nitrincola sp. KXZD1103	8	47	39	35	28	34	4	0	5	1	3	1	0	5	1	0
Pseudomonas cremoricolorata	7	7	5	12	23	20	0	0	6	11	15	3	9	49	44	0
Streptococcus sanguinis	41	12	34	23	24	36	7	1	14	1	6	1	1	9	0	0
Capnocytophaga gingivalis	39	32	53	33	28	13	2	0	1	4	0	3	0	2	0	0
Rhodobacteraceae bacterium QY30	27	20	21	19	26	12	6	6	17	11	7	7	4	13	14	0
Paracoccus suum	25	24	15	17	14	14	5	9	22	2	17	6	6	22	12	0
Peptacetobacter hiranonis	21	5	32	46	27	19	1	3	32	6	1	7	3	6	1	0
Chryseobacterium sp. IHB B 17019	40	25	42	26	17	19	0	7	16	3	5	1	1	3	4	0
Pleomorphomonas sp. SM30	33	11	23	23	35	18	6	8	13	6	6	4	3	13	7	0
Massilia flava	28	29	32	31	39	18	3	3	11	1	1	4	1	4	4	0
Photobacterium gaetbulicola	21	37	16	31	52	17	4	2	13	4	1	1	0	4	6	0
Limnohabitans sp. 103DPR2	20	28	34	28	32	18	1	8	13	3	3	8	0	8	5	0
Mixta theicola	8	21	29	44	69	15	1	0	9	1	1	2	3	6	0	0
Pseudomonas sp. THAF7b	3	5	8	10	18	17	0	1	7	6	17	2	19	54	42	0
Pseudomonas sp. XWY-1	0	0	5	7	11	5	0	0	8	3	27	1	34	72	35	1
Orbus sp. IPMB12	35	6	20	25	80	18	2	3	6	1	2	3	4	3	0	0
Citrobacter rodentium	30	28	10	50	30	44	6	0	4	1	1	0	1	2	1	0
Pseudomonas azotoformans	13	27	11	19	16	17	2	1	14	3	11	4	14	25	31	0
Shewanella sp. YLB-08	6	129	9	5	12	35	2	0	2	1	3	0	1	2	1	0
Christensenella massiliensis	34	18	22	19	35	35	2	2	15	2	8	4	5	6	0	0
Obesumbacterium proteus	27	18	61	27	20	24	0	0	10	1	2	2	7	6	2	0
Sinorhizobium meliloti	25	9	23	32	22	21	6	5	15	6	9	8	9	10	7	0
Ewingella americana	25	47	31	47	8	23	1	4	6	0	5	2	3	4	1	0
Herbaspirillum frisingense	20	29	17	28	31	12	1	3	12	3	3	4	6	28	9	1
uncultured bacterium A1Q1_fos_150	10	8	16	11	91	56	1	2	3	1	2	1	3	1	1	0
Sphingomonas sp. CL5.1	69	8	8	10	10	16	2	2	24	14	12	6	1	14	10	0
Blautia sp. YL58	36	19	26	20	29	33	3	3	15	3	3	2	3	8	3	0
Yersinia aldovae	32	61	29	21	14	12	4	2	13	4	1	2	2	7	2	0
Massilia sp. YMA4	27	30	28	33	20	23	5	2	9	4	7	6	2	6	4	0
Acidihalobacter ferrooxydans	19	20	32	41	34	16	5	4	14	1	2	1	4	7	5	1
Vibrio zhugei	56	19	19	45	8	16	2	2	11	1	9	2	3	8	4	0
Pantoea sp. PSNIH1	34	20	19	23	51	26	1	1	13	1	4	1	5	6	0	0
uncultured bacterium BD_contig00795	33	41	15	31	25	20	2	5	7	1	6	7	2	9	1	0
Chryseobacterium lactis	23	25	45	29	25	20	2	4	18	1	5	3	1	4	0	0
Acinetobacter lactucae	11	15	19	24	11	26	5	5	11	2	6	2	3	13	52	0
Pseudomonas sp. Leaf58	1	2	7	9	9	10	1	1	6	4	15	5	23	61	50	1
Janthinobacterium sp. B9-8	45	23	16	35	37	18	0	3	14	2	2	0	3	5	1	0
Magnetospirillum sp. XM-1	28	22	22	24	35	22	4	5	14	3	1	5	6	6	7	0
Streptococcus sp. FDAARGOS_192	19	13	12	33	28	54	2	6	11	5	9	5	3	2	2	0
Arcobacter sp. FWKO B	15	68	21	29	28	25	1	1	11	1	2	0	2	0	0	0
Halarcobacter ebronensis	12	28	25	27	32	30	17	1	13	3	3	3	3	6	1	0
Enterobacter sp. RHBSTW-00175	36	16	19	25	18	46	1	2	21	2	7	3	1	4	2	0
Caulobacter sp. FWC26	25	30	28	22	19	27	1	4	14	0	4	12	1	12	4	0
Methylobacillus flagellatus	22	25	47	27	23	16	4	5	15	6	4	1	2	5	1	0
Sphingopyxis sp. QXT-31	21	17	12	10	15	8	6	10	18	6	10	14	6	12	38	0
Lysobacter lycopersici	19	6	15	31	12	10	12	9	27	5	7	11	4	28	7	0
Sideroxydans lithotrophicus	18	19	36	32	26	28	5	5	12	2	3	4	4	8	1	0
Bosea vaviloviae	17	19	14	14	18	15	5	12	31	9	12	8	7	9	13	0
uncultured Caudovirales phage	8	12	9	8	10	10	3	1	5	6	8	2	18	81	21	1
Pseudomonas sp. MRSN12121	7	9	10	9	19	17	4	1	7	6	4	4	20	51	35	0
Fusobacterium pseudoperiodonticum	37	28	19	46	17	19	2	4	15	2	2	2	2	6	1	0
Vibrio sp. HDW18	35	20	15	19	40	33	2	5	20	1	2	3	2	3	2	0
crAssphage cr4_1	26	24	35	26	21	25	6	7	15	0	1	4	0	10	2	0
Methylobacterium sp. C1	16	6	9	11	10	10	10	12	18	15	21	22	16	18	8	0
Variovorax sp. PAMC28562	13	16	28	32	36	27	4	4	10	8	6	1	2	10	5	0
Eubacterium maltosivorans	44	19	15	24	32	33	0	6	14	4	3	2	0	5	0	0
Collimonas pratensis	25	34	27	28	19	21	1	2	9	5	10	3	6	7	4	0
Acinetobacter sp. NEB149	21	24	45	15	19	18	5	5	9	4	2	10	9	10	5	0
Achromobacter deleyi	20	21	39	38	26	11	0	6	9	3	5	5	6	8	4	0
uncultured Sphingopyxis sp.	12	18	9	11	15	14	3	11	24	7	8	15	4	12	38	0
Shigella flexneri	34	12	23	21	25	23	7	5	13	8	6	1	8	9	5	0
Flavobacterium gilvum	30	40	44	23	15	15	4	3	10	3	4	2	4	3	0	0
Brenneria rubrifaciens	22	46	16	10	16	41	1	4	17	2	4	2	6	6	7	0
Erwiniaceae bacterium PD-1	18	41	19	5	22	61	4	0	14	2	1	2	2	8	0	0
Azorhizobium caulinodans	34	26	11	21	20	19	2	5	23	5	8	5	7	8	3	0
Klebsiella sp. RHBSTW-00484	27	16	13	64	17	22	4	3	15	1	1	1	3	6	4	0
Oceanimonas sp. GK1	22	30	21	32	32	36	3	1	3	1	1	2	1	8	4	0
Pseudomonas marincola	19	8	40	22	20	15	1	1	10	7	4	1	5	27	17	0
Pseudoalteromonas rubra	30	32	24	23	21	29	2	2	5	3	1	6	3	10	5	0
Salinimonas sp. G2-b	26	67	31	18	24	10	0	3	8	1	1	2	2	1	2	0
Pseudomonas sp. SWI44	2	6	10	10	6	5	0	2	7	3	21	1	18	67	38	0
Flavobacterium nackdongense	22	40	30	16	22	28	4	3	8	8	1	5	2	4	2	0
Massilia sp. LPB0304	20	28	21	32	24	21	4	3	7	3	5	4	4	12	7	0
Pseudomonas sp. R32	6	10	12	21	11	10	3	2	9	3	18	2	12	42	33	1
Desulfovibrio sulfodismutans	46	11	28	14	39	31	2	3	7	3	1	1	3	5	0	0
Shewanella sp. FDAARGOS_354	39	23	22	10	20	49	4	3	6	2	6	0	2	5	2	0
Sphingopyxis sp. OPL5	21	12	9	10	14	14	7	6	29	3	8	17	1	6	36	0
Solimonas sp. K1W22B-7	13	15	28	26	25	22	1	6	15	3	3	7	5	12	12	0
Erwinia persicina	13	16	47	32	34	23	4	1	6	2	2	3	3	4	3	0
Rothia mucilaginosa	11	12	22	15	18	27	6	4	30	15	9	11	5	4	4	0
Anaerostipes rhamnosivorans	31	9	23	37	26	26	0	4	14	4	1	2	6	6	3	0

Lachnospiraceae bacterium TB5	27	23	13	23	21	45	4	4	8	0	3	4	1	11	5	0
Paraburkholderia aromaticivorans	25	22	21	19	21	11	0	6	17	5	10	9	4	12	10	0
Runella sp. SP2	21	25	31	25	19	16	7	4	14	0	0	3	2	11	14	0
Methylorubrum extorquens	20	16	14	27	16	13	2	9	22	7	8	7	4	15	12	0
Malaciobacter canalis	10	24	55	44	24	16	0	5	3	0	1	1	5	4	0	0
Providencia rustigianii	30	24	25	25	18	29	2	1	20	0	1	4	4	6	2	0
Chryseobacterium antarcticum	26	21	35	21	27	24	3	3	14	3	3	4	2	5	0	0
Bifidobacterium animalis	25	7	14	26	34	36	0	3	25	6	5	4	2	3	1	0
Limnobaculum parvum	17	54	18	18	6	33	0	4	18	4	3	3	2	8	3	0
Lactiplantibacillus plantarum	16	41	23	31	33	24	5	1	6	2	2	0	1	4	2	0
Enterobacter sp. E76	13	46	39	31	15	21	2	2	10	1	6	2	0	3	0	0
Pseudomonas sp.	2	2	8	8	7	8	0	0	10	1	13	3	27	75	27	0
Flavobacterium anhuiense	29	37	38	17	17	15	3	5	14	1	2	3	4	4	1	0
Pectobacterium atrosepticum	16	26	13	12	44	34	3	1	17	4	3	5	4	7	1	0
Izhakiella sp. KSNA2	12	23	8	35	42	43	0	4	10	0	1	2	0	7	3	0
Fusarium vanettenii	2	1	3	0	2	0	1	0	3	0	0	0	3	3	172	0
Bacillus cereus	32	16	16	32	24	17	4	0	11	2	7	7	4	15	2	0
Enterobacter sp. EA-1	28	26	40	12	15	32	2	0	13	3	2	4	3	6	3	0
Oceanisphaera avium	22	18	17	50	41	18	1	3	4	0	0	4	5	4	2	0
Herbaspirillum huttiense	19	24	24	16	23	20	3	0	17	2	9	6	11	8	7	0
Acinetobacter baylyi	17	38	46	23	10	13	2	3	9	1	6	5	3	8	4	0
Vibrio taketomensis	17	37	15	15	50	19	7	1	11	0	4	2	3	5	2	0
Sphingopyxis sp. FD7	13	14	8	11	8	18	8	12	18	5	6	15	1	20	31	0
Ruminiclostridium sp. MA18	37	23	31	15	19	30	2	1	8	2	3	4	0	5	6	0
Massilia sp. CCM 8941	22	25	29	29	28	15	6	2	9	1	1	2	7	5	5	0
Butyrivibrio fibrisolvens	22	23	27	19	16	30	5	3	9	2	10	7	5	7	1	0
Pantoea sp. At-9b	17	24	19	26	27	40	4	2	10	0	1	4	3	7	2	0
Massilia violaceinigra	16	26	28	39	19	21	3	4	12	2	3	2	3	5	3	0
Enterobacteriaceae bacterium bta3-1	12	33	36	22	37	21	3	5	6	2	0	6	1	1	1	0
Providencia vermicola	10	54	25	15	23	46	0	0	5	0	2	0	2	4	0	0
Ferrimonas balearica	33	17	7	18	35	32	8	8	11	2	3	1	2	5	3	0
Oscillospiraceae bacterium LBM18003	33	19	24	17	34	31	0	2	9	0	3	1	3	7	2	0
Blastochloris viridis	30	16	24	15	28	23	3	6	11	5	4	6	2	10	2	0
Betaproteobacteria bacterium GR16-43	24	22	25	24	31	19	2	2	11	3	6	1	3	11	1	0
Pseudoalteromonas arabiensis	21	54	14	64	5	23	0	2	0	1	0	0	1	0	0	0
Xanthomonas arboricola	14	10	14	27	18	24	6	7	12	0	7	6	2	15	23	0
Sphingopyxis sp. PAMC25046	10	10	9	9	15	14	5	18	21	4	5	16	7	10	30	2
Pectobacterium wasabiae	18	26	74	8	10	19	6	1	7	0	7	2	3	2	1	0
Bordetella avium	14	22	29	26	16	27	4	4	13	10	2	4	1	8	4	0
Acinetobacter oleivorans	1	11	16	17	11	24	1	3	4	0	9	1	3	23	60	0
Kaistia sp. 32K	52	20	10	15	17	13	1	3	18	3	9	6	2	10	4	0
Enterococcus gilvus	22	127	15	1	2	7	2	0	2	2	1	0	2	0	0	0
Legionella pneumophila	15	10	14	46	54	23	2	1	7	1	1	1	3	5	0	0
Pseudomonas frederiksbergensis	14	13	15	23	15	11	5	1	12	5	12	2	5	35	13	2
Providencia stuartii	14	65	21	12	20	18	0	3	10	5	6	1	3	4	1	0
Shewanella amazonensis	34	50	17	14	17	19	2	3	13	2	6	2	2	1	0	0
Enterobacter cloacae complex sp.	29	12	26	11	7	32	7	4	19	5	5	4	6	13	2	0
Tabrizicola piscis	18	19	16	6	19	23	9	8	15	7	5	6	4	12	15	0
Citrobacter telavivum	14	7	10	51	10	11	3	0	11	4	1	3	6	8	43	0
uncultured eukaryote	12	13	13	13	27	17	8	22	13	4	2	16	7	11	4	0
Phreatobacter cathodiphilus	22	9	16	19	27	18	2	6	25	7	4	7	2	12	5	0
Moritella marina	20	55	15	30	22	25	1	4	5	0	0	0	2	2	0	0
Bradyrhizobium elkanii	13	7	4	3	9	5	9	13	21	11	14	18	13	16	25	0
Adlercreutzia equolifaciens	42	10	19	28	21	20	0	1	22	3	5	1	3	3	2	0
Bradyrhizobium erythrophlei	33	21	11	8	25	14	5	6	17	4	6	6	5	14	5	0
Terasakiella sp. SH-1	31	32	3	65	5	41	1	0	0	0	0	0	1	1	0	0
Lachnoanaerobaculum umeaense	26	20	22	18	24	28	0	6	11	2	3	3	5	12	0	0
Pantoea cypripedii	21	23	22	22	43	20	3	2	4	3	7	2	2	4	2	0
Caulobacter segnis	19	13	23	20	24	25	2	6	9	3	7	4	4	11	10	0
Bacteroides phage DAC15	0	2	0	1	49	102	0	0	1	3	20	0	0	0	2	0
Gallaecimonas mangrovi	40	45	18	23	15	11	3	1	5	0	1	0	3	9	5	0
Bacteroidetes bacterium UKL13-3	39	28	20	27	13	16	1	5	10	1	2	7	2	4	4	0
Enterobacter oligotrophicus	26	33	5	17	65	14	0	3	4	0	5	3	1	3	0	0
Martelella mediterranea	24	21	21	13	22	13	2	5	22	2	4	5	6	12	7	0
Paraburkholderia tropica	21	27	17	29	33	24	2	1	11	1	2	1	3	5	2	0
Photobacterium profundum	17	25	14	28	28	36	4	3	10	1	2	1	3	5	2	0
Gemmobacter sp. HYN0069	16	13	15	7	21	16	7	12	29	6	8	5	5	11	7	0
Mesorhizobium terrae	16	14	10	9	8	14	1	12	24	12	12	11	5	25	5	0
Burkholderia plantarii	15	18	25	23	25	20	1	3	14	3	7	7	5	7	5	0
Pseudomonas mediterranea	13	15	9	16	16	13	2	4	14	4	18	1	3	29	19	2
Bordetella bronchialis	15	14	31	20	33	16	0	3	13	1	6	8	7	7	3	0
Citrobacter sp. RHBSTW-00017	14	13	17	28	62	8	1	3	11	1	7	6	3	1	2	0
Aeromonas sp.	14	21	19	15	14	16	6	10	21	5	1	4	4	20	7	0
Shewanella livingstonensis	13	25	4	47	38	32	2	2	3	1	0	1	1	5	3	0
Pseudomonas saudimassiliensis	12	13	15	17	28	14	2	6	8	0	7	1	4	30	20	0
Scedosporium apiospermum	86	8	0	5	2	2	9	7	7	4	6	5	2	9	24	0
Chitinibacter fontanus	37	9	34	30	29	15	1	3	8	0	1	1	2	5	1	0
Flavobacterium sp. 140616W15	27	30	40	14	21	15	3	0	11	2	0	1	2	8	2	0
Stenotrophomonas sp. KCTC 12332	21	30	12	21	14	12	5	6	18	4	7	10	2	11	3	0
Herbaspirillum hiltneri	17	23	28	19	23	18	2	2	16	2	3	4	1	14	4	0
Scandinavium goeteborgense	17	29	20	22	24	39	3	1	9	4	2	0	4	2	0	0
Pseudomonas amygdali	8	42	6	27	38	13	0	0	9	5	2	6	4	11	5	0
Flavobacterium sp. M31R6	39	22	26	15	15	22	0	3	13	4	0	8	2	4	2	0
Salmonella bongori	30	20	28	26	27	14	1	3	11	1	2	3	2	6	1	0
Mucilaginibacter rubeus	23	23	16	14	25	41	2	4	11	2	3	5	1	4	1	0
Janthinobacterium sp. 17J80-10	20	12	27	24	31	18	0	4	16	2	3	4	4	6	4	0
Burkholderiales bacterium GJ-E10	24	16	26	26	24	16	7	0	9	1	3	5	2	12	3	0
Adlercreutzia sp. 8CFCBH1	24	25	21	12	26	12	4	6	18	0	4	5	9	5	3	0
all taxa with neither family nor genus classification	21	18	29	13	22	27	3	11	12	2	2	2	4	4	4	0

**Candidatus Azobacteroides pseudotrichonymphae**	19	23	33	15	27	30	4	4	9	0	2	3	2	3	0	0
**Janthinobacterium lividum**	18	20	21	29	26	16	3	1	12	3	5	2	6	8	4	0
**Alcanivorax sp. IO_7**	5	73	7	13	13	11	3	4	4	3	5	1	5	6	21	0
**Enterobacter sp. FY-07**	27	22	12	39	17	19	2	1	14	1	2	3	2	11	1	0
**Chryseobacterium sp.**	16	22	47	39	10	7	4	1	5	0	0	4	5	10	3	0
**Polynucleobacter necessarius**	39	16	24	19	14	17	4	7	6	3	4	4	5	6	4	0
**Sebaldella termitidis**	26	21	24	28	16	29	0	2	15	0	2	0	8	0	1	0
**Hyalella azteca**	18	21	35	31	21	14	1	3	9	1	3	1	4	7	3	0
**Aerosticca soli**	15	37	23	10	21	13	4	4	12	3	3	5	5	6	11	0
**Yersinia hibernica**	7	89	41	11	4	10	1	1	2	0	2	0	1	2	1	0
**Acidithiobacillus caldus**	17	25	20	25	25	22	1	4	11	1	3	3	3	7	4	0
**Massilia armeniaca**	14	22	29	16	24	21	4	5	5	2	6	3	1	11	8	0
**gamma proteobacterium HdN1**	12	8	46	40	23	18	3	3	6	0	2	6	3	1	0	0
**Kosakonia sp. CCTCC M2018092**	8	93	3	9	32	8	1	0	15	0	0	0	1	1	0	0
**Pseudomonas sp. NP-1**	4	7	11	10	6	12	1	0	10	3	18	2	22	34	31	0
**Vibrio gazogenes**	25	18	28	20	13	42	1	2	8	1	4	4	2	1	1	0
**Romboutsia sp. CE17**	13	14	19	18	5	16	6	13	18	11	6	10	10	9	2	0
**bacterium**	10	16	27	13	34	25	1	0	8	3	5	1	7	15	5	0
**Enterobacter sp. SA187**	10	26	34	21	43	16	0	4	8	0	3	1	0	2	2	0
**Rhizobium sp. ACO-34A**	20	13	12	32	15	24	3	3	15	5	4	7	2	11	3	0
**Rhodocyclaceae bacterium SHINM1**	15	20	27	35	16	14	0	4	17	2	2	3	3	9	2	0
**Pseudomonas rhodesiae**	9	9	12	13	15	14	1	4	13	5	5	3	14	30	22	0
**Enterobacter soli**	7	21	11	22	73	8	0	4	11	0	5	0	1	5	1	0
**Kosakonia pseudosacchari**	36	28	13	18	47	8	1	0	5	2	1	1	2	4	2	0
**Anaeromyxobacter dehalogenans**	25	27	19	14	24	15	3	6	8	1	2	6	4	12	2	0
**Orrella dioscoreae**	22	18	23	20	23	20	0	2	16	0	6	2	3	7	6	0
**Rheinheimera sp. D18**	73	19	12	12	14	16	2	1	8	1	1	0	3	3	2	0
**Methylorubrum populi**	26	26	14	6	24	13	3	5	12	4	1	10	3	11	9	0
**Stenotrophomonas nitritireducens**	26	24	14	19	8	22	5	4	24	2	3	7	2	5	2	0
**Pedobacter sp. CJ43**	26	27	16	20	23	19	1	2	8	5	5	3	6	5	1	0
**Lacticaseibacillus rhamnosus**	24	46	21	21	12	8	2	3	11	3	4	3	5	2	2	0
**Rhodobacteraceae bacterium**	20	20	12	15	18	11	0	7	13	9	7	6	6	13	10	0
**Planctomyces sp. SH-PL62**	18	16	10	6	20	4	10	10	24	3	10	14	6	11	5	0
**Arcobacter nitrofigilis**	16	35	31	22	14	15	3	1	8	1	3	5	3	6	4	0
**Xanthomonas hortorum**	21	26	20	12	8	24	3	3	10	3	6	3	5	16	6	0
**Miniimonas sp. S16**	21	9	16	10	9	13	6	2	23	7	4	7	12	12	13	2
**Pseudomonas thivervalensis**	7	4	7	8	13	10	6	2	9	4	9	4	14	39	29	1
**Campylobacter iguaniorum**	4	23	53	34	21	13	1	1	5	3	4	0	2	2	0	0
**Pseudomonas sp. EGD-AKN5**	0	2	140	5	3	5	1	0	2	4	0	1	0	2	1	0
**Vibrio zhuhaiensis**	30	18	11	41	43	5	2	0	5	1	4	0	0	1	4	0
**Azospirillum thermophilum**	22	15	23	15	27	21	3	4	8	1	6	2	1	11	6	0
**gamma proteobacterium SS-5**	21	21	18	26	35	12	3	2	6	0	2	3	3	8	5	0
**Microbacterium hominis**	17	12	11	13	18	14	1	3	22	8	6	5	7	21	7	0
**Starkeya novella**	15	8	15	14	12	24	3	12	18	6	9	5	9	9	6	0
**Leminorella richardii**	29	27	16	18	46	8	1	1	8	0	3	0	2	4	1	0
**uncultured Prevotella sp.**	27	22	27	20	13	28	0	0	8	3	3	5	2	4	2	0
**Malaciobacter molluscorum**	24	21	24	24	24	16	0	4	8	1	2	3	4	7	2	0
**Rhizobium phaseoli**	19	12	17	18	23	18	3	2	10	4	12	7	2	10	7	0
**Xenorhabdus doucetiae**	25	25	13	20	39	22	2	2	4	1	1	2	3	4	0	0
**Pantoea alhagi**	23	12	32	36	10	22	2	1	4	1	2	4	4	6	3	1
**Chryseobacterium gallinarum**	22	25	22	20	23	22	1	2	11	0	3	2	3	6	1	0
**Flavobacterium commune**	19	30	30	13	19	23	0	2	7	2	3	4	1	6	4	0
**Acinetobacter sp. Marseille-Q1620**	17	18	37	21	10	13	2	2	6	0	1	3	4	15	14	0
**Yersinia bercovieri**	14	10	20	36	19	28	1	3	25	2	1	1	0	1	2	0
**Leclercia sp. 119287**	7	80	1	5	19	33	0	2	8	1	2	0	2	2	1	0
**Bacteroides phage DAC17**	0	0	0	2	54	84	0	0	1	3	13	0	0	0	6	0
**Hyphomicrobium sp. MC1**	93	28	3	4	3	10	2	0	5	3	3	1	0	7	0	0
**Belliella baltica**	21	20	43	16	21	20	6	3	3	1	2	2	1	0	2	1
**Clostridium baratii**	19	17	22	14	8	13	2	22	15	5	3	2	12	7	1	0
**Sphingopyxis sp. EG6**	16	12	9	12	15	9	7	5	21	4	3	9	1	13	26	0
**Plautia stali symbiont**	10	23	8	8	73	13	0	2	8	1	3	0	1	10	2	0
**Psychromonas ingrahamii**	9	69	7	21	28	8	1	2	7	2	2	1	0	2	3	0
**Lonsdalea populi**	10	26	7	49	24	20	4	1	6	1	1	1	3	6	2	0
**Actinobacillus pleuropneumoniae**	10	46	20	11	9	44	3	4	4	0	0	1	1	6	2	0
**Streptococcus sp. A12**	30	31	21	11	20	16	2	3	10	2	4	6	1	3	0	0
**Flavobacterium sp. Sr18**	28	22	28	20	12	13	4	4	13	5	4	1	3	3	0	0
**Vibrio aquimaris**	20	19	3	20	39	43	0	0	2	0	1	0	6	6	1	0
**Tatumella ptyseos**	16	15	10	22	21	42	2	3	15	1	3	3	2	4	1	0
**Paraburkholderia phytofirmans**	12	15	12	14	20	16	7	8	9	9	9	8	6	12	3	0
**Aggregatibacter aphrophilus**	35	18	18	21	27	24	1	3	7	0	1	0	2	1	1	0
**Klebsiella sp. BDA134-6**	30	42	28	10	5	10	6	2	7	0	0	9	5	5	0	0
**Candidatus Nitrotoga fabula**	19	13	8	29	16	19	7	2	19	1	13	2	0	8	3	0
**Halomonas hydrothermalis**	14	10	6	3	7	10	5	7	16	12	22	11	21	12	3	0
**Enterobacteriaceae bacterium strain FGI 57**	10	15	40	12	33	18	5	2	7	0	2	3	2	9	1	0
**Pseudomonas sp. S-6-2**	9	11	19	21	27	12	0	2	11	2	6	6	3	19	11	0
**Thalassotalea sp. LPB0316**	9	75	13	5	6	32	2	2	6	2	1	1	0	5	0	0
**Pseudoxanthomonas spadix**	20	12	19	20	14	10	4	4	15	3	11	3	1	12	10	0
**Hungateiclostridiaceae bacterium KB18**	20	15	18	14	29	30	2	1	8	5	5	3	3	4	1	0
**Paraburkholderia sp. 7MH5**	18	21	20	20	23	24	0	2	10	1	3	4	1	4	7	0
**Enterobacteriaceae bacterium A-F18**	9	7	2	41	10	70	0	0	9	3	1	2	1	3	0	0
**Gordonibacter pamelaeae**	20	18	19	21	34	21	3	2	12	0	2	1	1	3	0	0
**Pectobacterium odoriferum**	16	15	31	26	18	18	2	1	11	0	2	3	2	8	4	0
**Aminobacter sp. SR38**	10	9	8	20	17	7	2	6	22	6	8	5	5	21	11	0
**Pantoea sp. CCBC3-3-1**	31	16	24	10	27	12	2	4	11	0	6	6	1	5	1	0
**Chitinophaga pinensis**	23	43	14	11	17	23	0	1	7	1	3	2	3	3	5	0
**Selenomonas sputigena**	14	19	22	23	32	15	1	1	6	3	3	1	4	10	2	0
**Providencia alcalifaciens**	14	15	33	37	17	16	3	5	5	0	5	1	3	2	0	0
**Pseudomonas umsongensis**	13	12	19	12	17	16	1	0	4	7	8	2	10	20	15	0
**Clostridium septicum**	12	9	35	9	19	11	5	9	8	4	6	8	8	6	7	0

Aeromonas sobria	8	13	24	31	17	8	0	10	20	2	6	4	2	11	0	0
Pseudomonas rhizosphaerae	7	9	4	16	13	10	2	1	11	3	10	3	8	31	28	0
Enterobacter sp. JUb54	4	11	66	19	16	16	1	0	7	0	4	2	1	6	3	0
Shewanella sp. Arc9-LZ	3	63	14	24	8	13	3	1	13	0	4	3	0	3	4	0
Pseudoalteromonas ruthenica	3	100	19	15	8	2	0	0	7	0	2	0	0	0	0	0
Methanobacterium lacus	39	6	16	19	24	18	2	2	8	9	4	2	2	4	0	0
Citrobacter sp. Y3	22	4	4	18	59	10	0	0	4	7	24	2	0	1	0	0
Bosea sp. PAMC 26642	17	15	16	14	13	8	5	6	20	5	7	7	2	15	4	1
Sphingobium baderi	14	0	7	27	76	7	1	1	6	1	1	2	2	5	5	0
Myroides phaeus	25	47	26	12	15	12	3	2	6	0	2	0	0	3	1	0
Paraburkholderia xenovorans	16	22	9	23	8	14	3	3	13	6	2	9	6	13	7	0
uncultured Acidovorax sp.	16	26	15	30	33	9	2	10	5	0	0	2	1	4	1	0
Chryseobacterium arthrosphaerae	15	26	28	18	17	21	2	2	10	0	1	5	0	8	1	0
Nissabacter sp. SGAir0207	14	26	14	14	14	35	2	1	15	0	4	3	2	5	5	0
Pseudoalteromonas atlantica	14	38	19	10	24	29	1	2	9	1	0	1	3	3	0	0
Aminipila butyrica	30	35	21	14	14	20	1	0	3	2	1	6	3	3	0	0
Novosphingobium tardaugens	18	13	16	14	12	8	5	9	28	3	7	3	7	6	4	0
Weissella confusa	23	14	2	16	27	19	0	0	19	8	8	0	0	12	4	0
Usitatibacter palustris	22	19	16	20	28	16	2	3	11	2	1	3	0	7	2	0
Microbacterium sp. No. 7	14	10	15	11	7	9	8	3	18	9	8	4	14	15	6	1
Paraburkholderia caribensis	12	14	14	16	19	28	5	5	11	2	5	1	3	11	5	1
Allochromatium vinosum	11	21	19	17	25	23	3	3	9	1	4	2	3	8	3	0
uncultured bacterium 1i11	28	10	8	24	19	30	3	4	10	0	4	0	5	6	0	0
Olsenella sp. GAM18	24	9	18	25	24	27	0	1	8	0	3	7	0	5	0	0
Acutalibacter muris	24	14	18	18	28	23	1	2	6	3	4	2	1	3	4	0
Ignavibacteriae bacterium	19	18	29	23	22	19	1	1	7	1	4	0	2	4	1	0
Citrobacter sp. RHBSTW-00986	18	17	5	21	66	11	1	4	3	0	1	3	1	0	0	0
Marinobacter hydrocarbonoclasticus	14	17	8	36	15	11	4	2	23	0	5	2	4	5	5	0
Pseudomonas corrugata	5	13	24	12	16	11	0	1	15	3	4	2	8	19	17	1
Weissella cibaria	46	2	12	30	17	12	4	0	13	0	4	4	0	6	0	0
Defluviicoccus vanus	41	15	8	16	14	4	4	6	14	4	8	4	1	2	9	0
Kosakonia sp. MUSA4	28	19	37	12	11	26	0	2	5	1	1	5	0	2	1	0
Ancylobacter sp. TS-1	23	14	13	16	23	13	5	2	8	5	7	0	7	12	2	0
Usitatibacter rugosus	18	14	19	26	19	17	5	0	11	1	1	5	5	3	6	0
Flavobacterium sp. xlx-214	17	26	19	12	20	19	5	5	9	4	2	2	3	5	2	0
Thalassotalea sp. HSM 43	12	35	11	4	39	39	0	1	3	2	0	1	1	1	1	0
Kerstersia gyiorum	5	5	12	16	69	13	1	2	5	2	0	3	3	10	4	0
Citrobacter sp. CRE-46	21	6	1	22	41	40	1	1	3	0	2	1	1	5	4	0
Buttiauxella agrestis	21	22	25	14	9	18	2	6	14	0	3	6	2	7	0	0
Azospirillum ramasamyi	17	10	33	16	24	12	2	1	10	3	4	1	4	9	3	0
Cupriavidus malaysiensis	14	16	18	23	17	16	1	1	15	2	8	0	2	11	5	0
Salinivibrio sp. YCSC6	13	9	28	26	41	14	1	3	6	1	2	3	2	0	0	0
Neisseria elongata	12	24	20	16	18	19	4	1	13	1	3	5	4	7	2	0
Shewanella sp. M2	12	39	7	33	18	10	5	2	6	4	2	1	3	6	1	0
Pseudoalteromonas sp. R3	9	12	9	41	13	43	3	1	4	1	1	3	2	6	1	0
Verrucomicrobium sp. GAS474	36	21	13	11	13	20	5	2	11	3	3	3	3	3	1	0
Citrobacter sp. SNU WT2	24	11	9	17	15	23	1	3	14	1	2	7	14	5	2	0
Janthinobacterium sp. SNU WT3	20	7	21	12	15	49	1	1	5	6	3	2	0	3	3	0
Neisseria meningitidis	19	16	19	24	25	14	1	0	8	4	5	4	3	5	1	0
Vibrio chagasii	19	4	13	4	71	30	0	1	3	0	0	0	0	3	0	0
Citrobacter sp. FDAARGOS_156	19	11	14	24	22	32	2	0	11	0	3	3	3	2	2	0
Chryseobacterium sp. T16E-39	18	22	31	20	18	20	0	2	12	0	0	1	2	2	0	0
Chryseobacterium aureum	15	34	19	18	15	19	0	4	13	1	0	5	2	3	0	0
Arachidicoccus soli	14	32	38	20	12	14	0	0	12	0	5	1	0	0	0	0
Vibrio sp. EJY3	12	23	17	35	10	22	1	0	12	3	2	7	1	3	0	0
Agrobacterium rhizogenes	11	14	20	11	20	20	3	14	5	10	7	5	2	3	3	0
Pseudomonas sp. SNU WT1	6	9	12	14	12	10	3	0	4	2	13	3	12	23	24	1
Rhodanobacter glycinis	39	16	14	17	12	7	0	3	10	4	6	6	1	8	4	0
Methyloprofundus sp.	30	28	6	35	3	27	0	1	14	2	0	0	0	1	0	0
Streptococcus pyogenes	20	20	15	24	14	17	1	4	11	2	1	4	4	10	0	0
Paludibaculum fermentans	18	8	17	12	6	18	5	5	6	1	5	42	1	2	1	0
Pandoraea sp. XY-2	18	16	22	21	16	13	1	3	15	2	2	4	3	2	9	0
Chryseobacterium sp. StRB126	17	15	24	16	15	23	3	0	10	2	4	2	3	11	2	0
Edwardsiella piscicida	15	13	26	17	9	18	6	2	10	3	16	2	3	2	5	0
Acinetobacter sp.	14	16	41	17	7	7	2	10	7	2	1	2	0	10	11	0
Anaerotignum propionicum	32	13	24	9	13	25	1	1	10	2	4	5	0	2	5	0
Roseomonas mucosa	24	9	11	17	11	11	2	4	11	2	12	10	2	15	5	0
Yokenella regensburgei	23	50	15	5	11	12	1	0	16	1	5	1	3	3	0	0
Chryseobacterium piperi	23	24	26	21	19	15	3	0	9	1	1	0	2	2	0	0
Bradyrhizobium japonicum	17	15	12	9	10	8	3	8	12	5	6	16	8	14	3	0
Rhizobium oryzihabitans	15	13	13	15	9	17	5	3	24	3	7	1	3	12	6	0
Alteromonas macleodii	15	10	13	8	52	14	5	4	2	2	3	5	4	7	2	0
Neisseria mucosa	14	28	10	25	12	13	6	1	10	14	2	1	1	7	2	0
Pseudopropionibacterium propionicum	13	10	17	12	15	16	3	7	24	2	6	0	7	5	9	0
Bradyrhizobium sp. CCBAU 21365	12	4	5	6	5	5	10	11	17	8	11	16	16	10	10	0
Lysobacter capsici	11	7	23	15	9	17	1	8	27	2	3	5	2	11	5	0
Mycobacterium dioxanotrophicus	9	3	8	10	6	2	6	16	21	8	12	7	13	15	10	0
Flavobacterium sp. MDT1-60	30	14	24	17	10	18	3	1	10	1	0	6	4	5	2	0
uncultured bacterium BD_contig01900	16	35	13	12	17	28	4	0	5	0	4	2	0	5	4	0
Flavihumibacter sp. SB-02	15	22	21	27	10	13	3	2	10	1	3	2	8	2	6	0
Pelagibaca abyssi	12	18	6	9	16	7	4	7	22	11	10	2	8	7	6	0
Micrococcus luteus	10	18	16	21	10	10	7	9	17	1	6	8	6	4	2	0
Bacillus aryabhattai	8	3	7	7	7	21	0	1	7	2	4	2	7	34	35	0
Pseudomonas sp. 02C 26	5	9	9	15	14	6	1	0	8	6	8	3	10	28	23	0
Mannheimia varigena	34	22	32	6	11	17	3	2	6	3	1	2	2	2	1	0
Oblitimonas alkaliphila	27	29	6	7	5	24	1	1	0	2	6	0	2	18	16	0
Rhizobium pusense	17	9	15	8	16	16	2	9	15	6	2	5	7	11	6	0
Salinimonas lutimaris	39	20	12	12	12	17	3	0	9	1	7	4	2	3	2	0
Glaciecola amylolytica	26	20	30	4	17	16	4	8	4	2	1	3	2	6	0	0

**Oceanospirillaceae bacterium ASO5**	25	10	6	41	8	25	0	2	5	1	6	2	1	8	3	0
**Yersinia mollaretii**	21	16	10	11	25	35	1	3	8	1	5	2	1	2	2	0
**Ruminococcaceae bacterium CPB6**	19	14	23	11	14	35	4	1	4	2	8	3	2	3	0	0
**Cupriavidus pinatubonensis**	17	18	17	16	22	13	1	2	4	1	5	5	4	7	11	0
**Mixta intestinalis**	14	7	13	31	32	15	4	1	11	2	4	4	1	4	0	0
**Sphingopyxis alaskensis**	7	15	12	11	8	11	3	4	13	0	5	14	3	11	26	0
**Elusimicrobium minutum**	55	30	22	4	5	13	1	0	2	3	1	2	0	2	2	0
**Faecalibacterium virus Taranis**	28	12	19	15	14	27	4	4	9	0	2	0	1	5	2	0
**Achromobacter pestifer**	18	14	19	13	16	20	1	4	5	3	6	5	4	6	8	0
**Marichromatium purpuratum**	17	11	24	10	18	14	2	1	11	1	6	3	2	13	9	0
**Mannheimia haemolytica**	12	13	19	15	37	18	0	1	9	2	7	3	2	4	0	0
**Sphingobacterium hotanense**	8	1	12	6	86	8	2	3	8	0	2	1	3	2	0	0
**Shigella sonnei**	7	6	7	12	20	41	4	1	11	4	6	7	3	8	5	0
**Vibrio tapetis**	5	10	36	26	30	13	3	0	12	0	3	3	1	0	0	0
**Methylomonas sp. LW13**	50	10	23	24	8	5	0	0	5	2	2	4	1	6	1	0
**Capnocytophaga sputigena**	25	17	19	13	19	22	4	1	4	3	6	4	0	2	2	0
**Sulfuriferula sp. AH1**	10	12	14	21	20	41	2	0	5	1	2	4	1	8	0	0
**Rhodobacter sp. CZR27**	9	9	17	20	9	10	3	3	13	5	9	10	8	10	6	0
**Parolsenella catena**	25	6	13	7	33	24	0	6	12	6	1	2	2	3	0	0
**Flavobacterium crocinum**	23	21	22	8	20	15	2	3	5	2	7	5	2	4	1	0
**Cupriavidus sp. USMAHM13**	20	16	15	20	9	10	4	3	13	2	7	2	7	8	4	0
**Lactobacillus harbinensis**	20	42	46	4	1	2	0	3	3	0	0	7	4	6	2	0
**Geobacter sp. FeAm09**	15	13	14	21	20	26	2	2	6	7	6	0	0	1	7	0
**Lysobacter antibioticus**	12	14	17	12	15	5	7	2	18	5	9	2	11	8	3	0
**Lysobacter maris**	10	14	10	10	8	5	6	5	16	2	4	6	27	9	8	0
**Cronobacter dublinensis**	4	19	14	28	6	45	1	3	15	1	3	0	1	0	0	0
**Edwardsiella hoshinae**	25	9	8	46	24	6	2	1	7	3	2	1	1	3	1	0
**Rhizobium etli**	19	10	14	14	20	14	2	3	11	4	14	0	3	6	5	0
**Sphingobium sp. TKS**	14	7	13	5	15	12	5	7	16	2	6	6	4	16	11	0
**uncultured bacterium 52B7**	9	13	39	29	9	13	4	0	10	0	6	2	2	3	0	0
**Gardnerella vaginalis**	22	18	33	6	16	11	1	1	13	0	2	2	0	9	4	0
**Enterococcus saigonensis**	13	16	18	10	11	32	0	6	12	3	4	4	0	8	1	0
**Metakosakonia sp. MRY16-398**	8	19	4	39	49	7	1	2	5	0	1	2	0	1	0	0
**Photorhabdus asymbiotica**	7	16	33	12	15	22	4	2	9	3	1	4	3	3	4	0
**Sphingobium herbicidovorans**	77	5	4	7	6	6	2	5	5	0	0	1	2	8	9	0
**Pseudomonas brenneri**	54	4	7	8	10	11	0	1	6	2	4	1	4	17	8	0
**Oceanihabitans sp. IOP_32**	36	16	20	13	18	13	2	1	6	0	4	1	1	5	1	0
**Methylocaldum marinum**	22	16	19	19	20	15	3	2	5	1	3	3	0	3	5	0
**Thioalkalivibrio sulfidiphilus**	18	20	12	14	10	10	2	21	5	0	4	4	2	9	5	0
**Massilia plicata**	18	23	15	18	17	9	1	3	7	1	3	4	5	8	3	1
**Flavobacterium sp. KBS0721**	17	21	20	18	20	17	2	4	7	0	1	5	2	2	0	0
**Erwinia tasmaniensis**	15	8	24	15	29	18	2	2	17	2	1	1	0	1	1	0
**Bordetella sp. N**	14	9	22	16	21	12	1	2	8	4	6	3	2	7	9	0
**Photorhabdus laumondii**	22	22	17	18	10	22	1	0	10	4	2	0	3	3	1	0
**Kaistella daneshvariae**	16	20	26	13	14	25	0	3	7	4	4	1	1	1	0	0
**uncultured bacterium BD_contig02839**	30	8	15	12	11	40	7	2	6	0	0	2	0	1	0	0
**Gordonibacter urolithinfaciens**	25	13	31	20	14	9	0	3	7	1	1	2	4	1	3	0
**Luteimonas sp. YGD11-2**	21	9	14	11	11	6	6	5	22	1	3	5	3	12	5	0
**Citrobacter sp. RHBSTW-00881**	17	13	8	10	31	25	0	4	6	1	5	1	1	6	6	0
**Cronobacter turicensis**	13	58	5	7	10	20	0	3	6	3	2	2	2	2	1	0
**Acidipropionibacterium jensenii**	9	10	10	9	20	14	3	6	14	4	4	7	1	16	7	0
**Emticicia oligotrophica**	9	35	15	10	18	31	2	2	6	0	1	2	1	2	0	0
**Bordetella sp. J329**	18	18	19	20	22	9	1	2	8	0	2	6	2	3	3	0
**Fluviicola taffensis**	18	17	25	10	20	19	3	3	9	0	1	1	1	2	4	0
**Burkholderia ambifaria**	14	13	15	17	17	25	2	1	9	3	1	5	2	6	2	1
**Xenorhabdus hominickii**	14	15	28	15	15	11	1	5	9	1	4	4	6	5	0	0
**Burkholderia lata**	11	16	25	15	10	16	2	4	12	0	3	5	3	6	5	0
**Pseudoalteromonas tunicata**	46	13	10	25	11	9	0	2	4	2	3	2	0	2	3	0
**Leucobacter triazinivorans**	24	5	11	9	7	7	6	6	19	3	6	8	2	12	7	0
**Flavobacterium nitrogenifigens**	17	17	29	10	10	18	3	2	10	1	2	5	2	5	1	0
**Celeribacter indicus**	16	7	4	10	12	16	3	11	20	5	7	2	6	4	9	0
**Vibrio casei**	15	15	27	14	12	12	2	3	21	5	0	3	1	2	0	0
**Ferriphaselus amnicola**	14	22	11	23	20	16	2	3	6	1	2	2	2	4	4	0
**Aliivibrio wodanis**	14	20	28	12	14	8	3	6	8	1	2	6	6	4	0	0
**Chelatococcus sp. CO-6**	13	8	12	11	9	13	1	4	22	7	5	8	2	12	5	0
**Thermomonas carbonis**	9	12	11	10	10	8	1	4	18	9	3	8	5	17	7	0
**Pseudomonas sp. SWI6**	5	8	6	3	6	7	1	2	2	6	3	4	8	54	15	2
**Treponema sp. RCC2812**	19	11	18	19	13	28	1	1	1	4	3	1	2	5	5	0
**Sideroxydans sp. CL21**	16	19	10	19	24	17	3	2	8	0	3	1	1	6	2	0
**Malaciobacter marinus**	10	17	13	40	17	17	0	2	8	3	2	1	0	0	1	0
**Pseudomonas extremaustralis**	4	8	8	16	6	5	2	1	3	3	12	1	13	28	20	1
**Clostridium pasteurianum**	26	24	10	16	19	19	2	3	9	0	1	1	0	0	0	0
**Erwinia amylovora**	25	11	13	20	15	14	2	0	10	1	4	0	4	9	2	0
**Alkaliphilus metalliredigens**	21	13	13	18	15	30	1	0	7	0	2	1	4	4	1	0
**Bordetella holmesii**	16	16	18	15	18	18	5	1	5	2	4	5	1	1	5	0
**Shewanella piezotolerans**	16	1	7	32	32	7	6	4	8	2	5	0	2	7	1	0
**Cedecea sp. FDAARGOS_727**	15	11	10	44	8	23	2	1	6	0	1	2	1	5	1	0
**Glaesserella parasuis**	15	20	17	19	11	13	2	4	8	6	2	0	3	6	4	0
**Novosphingobium aromaticivorans**	11	7	6	9	7	7	2	9	14	2	5	12	6	10	23	0
**Sphingomonas paucimobilis**	5	9	13	11	10	13	1	5	13	6	5	12	4	7	16	0
**Enterobacter sp. RHB15-C17**	34	11	32	6	5	27	1	2	5	1	1	1	0	1	2	0
**Chryseobacterium sp. G0186**	22	21	22	12	19	15	2	4	6	0	1	1	2	0	2	0
**Polymorphum gilvum**	20	13	9	13	14	10	0	4	16	4	5	2	4	10	5	0
**Stenotrophomonas sp. LM091**	13	20	15	21	12	12	2	1	10	1	1	1	4	11	5	0
**Flavobacterium sp. SLB02**	12	22	29	16	24	6	1	3	1	3	5	1	2	4	0	0
**Stella humosa**	21	12	9	5	35	7	0	1	10	7	2	3	5	9	2	0
**Mixta calida**	21	5	12	43	13	13	2	3	5	1	3	0	0	4	3	0
**Faecalibacterium virus Brigit**	17	8	16	11	11	41	2	0	12	2	2	0	4	0	2	0
**Agrobacterium vitis**	14	9	18	16	18	13	1	3	12	5	4	3	0	8	4	0

Thiocystis violascens	12	22	12	16	13	16	6	0	13	0	1	4	5	4	4	0
Burkholderia stabilis	11	13	16	18	14	13	3	2	12	3	4	4	3	6	6	0
Erysipelothrix rhusiopathiae	9	6	14	56	16	13	0	1	4	0	1	2	4	2	0	0
Yersinia sp. KBS0713	8	22	10	21	42	6	2	2	5	1	2	2	0	3	2	0
Pseudomonas salegens	6	32	6	9	6	12	2	2	5	23	4	3	1	8	8	1
Pseudomonas fuscovaginae	3	4	2	11	16	7	0	1	6	4	11	4	6	33	20	0
Buchnera aphidicola	24	21	14	14	13	18	4	0	9	2	2	2	1	3	0	0
Solitalea canadensis	24	18	17	15	8	17	4	3	5	0	2	7	1	6	0	0
Salinimonas sediminis	20	33	14	12	17	19	1	2	2	0	2	1	0	4	0	0
Sphingobium hydrophobicum	9	3	7	7	4	4	3	2	9	2	8	26	9	9	25	0
Bordetella genomosp. 9	8	9	19	13	24	15	3	1	8	5	2	4	2	7	7	0
Rhodospirillum rubrum	22	15	20	14	15	12	2	3	1	2	6	3	1	5	5	0
Klebsiella sp. FDAARGOS_511	22	38	18	9	1	6	8	2	9	1	0	4	5	2	1	0
Neorhizobium galegae	16	13	10	8	17	15	1	3	12	2	5	4	4	10	6	0
Polaribacter sp. L3A8	13	24	20	13	18	21	2	5	3	1	3	0	0	2	1	0
Alcanivorax pacificus	12	7	15	22	21	16	0	1	7	0	1	4	3	3	14	0
Vibrio sp. 2521-89	5	20	26	26	5	32	1	0	4	1	1	1	1	0	3	0
Pseudomonas migulae	4	22	2	16	12	6	1	3	13	4	7	3	6	13	14	0
Peptoclostridium acidaminophilum	26	18	27	12	14	13	1	0	10	0	0	0	3	1	0	0
Agarivorans gilvus	26	11	19	24	14	10	0	2	9	1	2	1	1	4	1	0
Corallococcus coralloides	23	9	11	3	9	10	5	2	22	1	2	14	3	8	3	0
Phreatobacter stygius	16	4	13	9	13	18	5	5	9	8	8	6	3	3	5	0
Xanthomonas sacchari	14	16	18	9	4	14	1	3	14	3	6	4	4	11	4	0
Pigmentiphaga aceris	13	9	13	18	16	20	6	2	7	3	2	4	4	3	5	0
Pseudomonas xanthomarina	7	7	7	15	15	9	1	3	10	0	7	0	4	20	20	0
Dyella sp. G9	16	16	8	13	18	14	1	6	4	3	4	7	3	8	3	0
Xanthomonas hyacinthi	13	10	13	10	14	12	2	3	14	2	5	8	5	9	4	0
Mameliella alba	11	8	12	12	14	10	3	6	7	6	5	4	6	14	6	0
[Enterobacter] lignolyticus	11	27	13	10	8	36	0	2	3	2	2	1	3	4	2	0
Vibrio mediterranei	9	39	9	12	11	27	0	3	4	2	2	2	2	1	1	0
crAssphage cr124_1	7	11	4	13	5	56	0	5	8	0	9	0	2	4	0	0
Citrobacter sp. RHBSTW-00570	24	19	6	14	19	17	1	4	6	2	4	2	0	3	2	0
Herbaspirillum sp. meg3	22	10	14	16	12	17	1	2	9	1	1	5	2	9	2	0
Sulfuricella denitrificans	16	21	17	17	17	8	2	2	10	1	2	4	1	3	2	0
Yersinia aleksiciae	16	17	15	18	12	21	1	1	10	1	2	6	0	3	0	0
Parastrongyloides trichosuri	14	14	8	18	15	12	5	4	11	5	1	1	2	7	6	0
Bordetella genomosp. 8	10	14	16	20	11	25	2	0	8	3	3	2	1	8	0	0
Romboutsia hominis	10	7	8	10	12	11	11	12	10	5	6	7	5	6	3	0
Candidatus Symbiobacter mobilis	7	14	15	22	22	13	5	3	9	0	2	1	2	4	4	0
Azospirillum thiophilum	15	15	10	20	19	12	2	2	9	1	2	4	3	6	2	0
Duncaniella sp. C9	15	14	11	18	14	24	3	3	10	0	2	3	0	4	1	0
Blastochloris tepida	23	13	13	12	9	12	3	1	9	1	2	10	1	7	5	0
Flavobacteriaceae bacterium 3519-10	16	30	21	11	18	6	1	2	9	1	3	2	1	0	0	0
Pseudomonas sabulinigri	15	12	15	15	6	17	0	3	7	3	4	2	3	14	5	0
Pseudoalteromonas sp. SM1988	12	0	19	42	21	18	0	1	4	0	2	0	1	1	0	0
Salipiger pacificus	11	15	3	12	10	13	0	8	15	3	6	6	7	7	5	0
Shewanella sp. YLB-09	10	2	80	3	5	8	1	2	2	0	0	1	2	4	1	0
Proteus hauseri	8	41	12	19	15	11	0	2	4	3	1	0	1	3	1	0
uncultured Limnohabitans sp.	6	67	12	7	11	5	0	2	4	4	0	0	0	2	1	0
Erwinia sp. QL-Z3	4	18	25	7	33	17	0	0	6	1	3	0	0	6	1	0
Legionella spiritensis	1	0	49	2	0	68	0	0	0	0	0	0	0	0	1	0
Starkeya sp. ORNL1	24	11	6	10	11	7	1	1	11	7	4	10	5	11	1	0
Campylobacter fetus	23	21	18	18	11	13	0	0	6	1	3	1	2	1	2	0
Friedmanniella luteola	16	10	5	12	12	9	3	4	17	3	3	4	5	13	4	0
Salinivirga cyanobacteriivorans	16	22	13	14	16	17	0	1	8	1	2	3	1	5	1	0
Achromobacter sp. MFA1 R4	13	15	19	9	15	16	0	4	10	4	6	1	2	3	3	0
Gallionella capsiferriformans	11	15	10	33	19	10	1	2	10	2	1	3	0	2	1	0
Paeniclostridium sordellii	10	15	19	17	8	12	3	6	10	4	2	5	3	4	2	0
Bacillus thuringiensis	9	30	16	1	13	11	2	2	4	5	4	4	5	10	4	0
Leclercia sp. 29361	7	15	14	10	37	14	3	0	5	1	4	4	0	3	3	0
Bordetella pseudohinzii	15	14	16	13	12	16	1	1	9	2	4	6	1	7	2	0
Mucilaginibacter gossypii	15	19	20	12	17	11	5	1	8	2	3	1	0	4	1	0
Chryseobacterium sp. JV274	14	13	16	19	15	16	5	4	6	3	1	1	2	2	2	0
Leadbetterella byssophila	12	18	10	22	14	11	4	2	4	0	4	7	2	1	8	0
Bradyrhizobium sp. CCBAU 51753	12	5	6	6	10	2	4	10	9	4	15	11	10	11	4	0
Chryseobacterium joostei	12	26	18	23	12	9	1	1	5	1	1	3	0	4	3	0
Luteimonas sp. MC1572	11	7	6	13	13	10	5	5	16	2	8	6	4	6	7	0
Janthinobacterium sp. 1_2014MBL_MicDiv	11	13	14	14	15	16	3	3	14	1	0	7	0	5	3	0
Acidovorax temperans	11	25	23	23	7	17	2	0	3	0	0	3	1	4	0	0
Pseudomonas litoralis	4	26	9	10	22	10	0	1	5	1	7	4	4	10	5	1
Pseudomonas sp. ZM1	0	0	5	4	5	7	0	1	3	1	6	0	4	38	44	1
Devosia sp. D6-9	48	10	7	8	8	2	4	0	17	0	5	2	1	5	1	0
Desulfolutivibrio sulfoxidireducens	25	12	17	15	19	13	1	0	3	1	3	4	0	2	3	0
Phenylobacterium zucineum	21	10	10	8	20	2	1	3	9	4	6	4	5	9	6	0
Flavobacterium faecale	20	18	17	16	15	12	3	1	7	1	3	3	0	1	1	0
Nitrobacter hamburgensis	19	4	10	14	6	8	6	3	15	3	8	9	2	5	6	0
Cellvibrio japonicus	18	9	35	10	7	20	1	3	2	0	2	2	3	3	3	0
Sphingomonas sp. MM-1	16	8	11	10	5	10	1	4	22	3	6	6	4	8	4	0
Xanthomonas phaseoli	13	22	19	12	18	15	1	1	3	0	1	1	3	6	3	0
Azospirillum sp. TSH100	11	5	15	15	10	13	5	8	10	4	2	6	2	5	6	1
Pandoraea pulmonicola	11	15	19	20	14	9	4	0	4	2	2	5	2	8	3	0
Ligilactobacillus ruminis	11	35	20	10	7	6	6	2	4	1	1	11	0	4	0	0
Lysobacter oculi	8	7	13	7	22	13	3	5	15	1	3	3	4	12	2	0
Methanoregula formicica	77	15	3	7	5	6	0	0	1	0	0	0	1	2	0	0
Chryseobacterium sp. G0162	18	14	27	12	15	18	1	1	9	1	0	0	0	1	0	0
Asticcacaulis excentricus	13	17	6	10	7	24	5	1	13	2	0	4	2	8	5	0
Streptococcus sp. I-P16	13	19	16	13	9	18	2	4	5	4	3	3	2	6	0	0
Winogradskyella forsetii	11	46	11	9	21	5	1	1	5	0	1	2	1	1	2	0
Phage FAKO27_000238F	10	16	20	16	14	22	0	4	7	0	0	2	2	4	0	0

**Mixta gaviniae**	9	23	15	13	19	13	3	4	7	2	3	0	2	2	2	0
**Iodobacter sp. H11R3**	8	40	11	16	13	12	3	3	3	1	0	0	2	5	0	0
**Flavobacterium sp.**	7	24	23	16	18	12	1	0	11	0	1	0	2	2	0	0
**Vibrio owensii**	7	14	8	29	16	14	1	3	10	0	4	2	5	4	0	0
**Ferrovum myxofaciens**	14	17	12	22	25	13	0	0	2	0	2	3	1	4	1	0
**Bradyrhizobium guangdongense**	13	2	10	8	13	9	6	3	17	3	8	6	3	9	6	0
**Aquisphaera giovannonii**	13	17	11	11	14	5	3	3	12	1	5	5	5	9	2	0
**Luteimonas granuli**	11	13	8	11	16	8	0	5	15	1	10	3	2	11	2	0
**Shewanella frigidimarina**	11	15	7	10	31	16	2	0	14	3	2	0	0	4	1	0
**Sphingobium amiense**	10	5	10	6	14	6	5	3	16	2	2	7	1	11	18	0
**Haliscomenobacter hydrossis**	10	13	27	16	15	15	2	0	8	2	0	2	3	1	2	0
**Xenorhabdus poinarii**	9	17	7	17	44	4	2	2	2	1	1	1	1	4	4	0
**[Pasteurella] aerogenes**	2	7	52	3	28	9	0	3	7	0	0	2	3	0	0	0
**Pseudodesulfovibrio aespoeensis**	27	13	17	14	15	11	4	2	3	3	0	1	2	2	1	0
**Salinivibrio costicola**	24	34	8	14	13	7	3	1	2	1	2	1	1	2	2	0
**Streptococcus sp. oral taxon 061**	21	8	20	11	12	19	4	0	3	0	7	0	1	7	2	0
**Vibrio panuliri**	16	11	9	19	10	15	1	1	21	2	0	3	3	1	3	0
**Mesorhizobium japonicum**	15	9	7	7	12	7	3	7	18	3	2	1	9	9	6	0
**Desulfomicrobium baculatum**	14	8	16	13	8	16	1	5	16	2	0	0	2	10	4	0
**Streptococcus koreensis**	14	14	16	14	10	18	1	0	6	1	13	2	2	3	1	0
**Clostridium chauvoei**	11	9	14	8	6	8	2	13	13	2	5	4	10	9	1	0
**Lysobacter sp. II4**	10	9	13	13	9	8	2	4	20	2	2	7	2	8	6	0
**Thermomonas sp. HDW16**	9	17	16	6	11	11	5	6	7	4	6	1	2	12	2	0
**Acinetobacter larvae**	8	11	18	15	12	15	1	3	5	3	3	2	3	10	6	0
**Halomonas aestuarii**	6	6	3	3	26	29	2	2	2	1	3	3	7	13	9	0
**Bradyrhizobium sp. BTAi1**	27	10	6	3	10	6	1	4	4	3	3	10	8	3	16	0
**Desulfarculus baarsii**	18	12	12	9	24	18	1	3	4	1	3	1	3	3	2	0
**Actinobacillus succinogenes**	16	8	19	8	32	11	1	1	6	0	3	1	3	3	2	0
**Bdellovibrio bacteriovorus**	15	14	16	16	12	17	4	2	10	0	0	2	0	6	0	0
**Empedobacter stercoris**	14	7	23	21	13	17	1	1	5	0	1	2	2	3	4	0
**Rhodovulum sp. MB263**	13	14	11	8	11	5	2	2	17	1	7	10	5	4	4	0
**Arcobacter peruensis**	12	14	14	17	15	19	0	2	6	2	1	2	4	6	0	0
**Lacimicrobium alkaliphilum**	10	11	11	33	6	12	1	4	8	1	3	4	2	4	4	0
**uncultured bacterium A1Q1_fos_660**	8	8	10	16	19	13	8	3	10	0	3	6	1	5	4	0
**Mesorhizobium sp. Pch-S**	7	10	9	11	8	12	3	2	13	1	11	3	9	9	6	0
**Sphingobacterium sp. UDSM-2020**	7	11	49	17	6	13	1	2	2	0	1	1	3	1	0	0
**Pseudoalteromonas luteoviolacea**	39	8	5	8	33	10	1	0	1	2	1	1	2	2	0	0
**Klebsiella sp. WP3-S18-ESBL-05**	22	13	4	8	7	19	9	2	7	6	5	4	2	4	1	0
**Cellulosilyticum sp. WCF-2**	21	18	16	9	16	14	4	0	3	2	2	2	2	4	0	0
**Desulfosarcina alkanivorans**	18	15	15	11	17	19	2	1	3	1	6	3	0	2	0	0
**Agrobacterium fabrum**	18	3	8	30	6	11	2	3	11	4	2	6	2	4	3	0
**Bradyrhizobium symbiodeficiens**	17	7	9	11	12	11	4	5	15	2	10	5	0	3	2	0
**Cedecea lapagei**	17	6	9	6	46	10	1	1	9	0	2	1	0	5	0	0
**Dyella japonica**	15	8	14	13	11	14	3	2	16	0	2	5	2	4	4	0
**Streptococcus mutans**	15	13	23	14	13	20	0	0	5	4	4	0	2	0	0	0
**Novosphingobium sp. THN1**	11	5	3	9	4	10	4	5	12	0	3	7	6	12	22	0
**Pseudomonas sp. 7SR1**	9	3	4	12	8	14	3	0	7	3	9	3	6	21	11	0
**Intestinibaculum porci**	9	21	14	8	16	15	0	5	8	1	6	3	1	5	1	0
**Flammeovirga pectinis**	9	10	12	9	57	6	1	3	2	0	2	0	1	1	0	0
**Luteimonas sp. JM171**	5	8	9	10	9	14	5	4	22	5	5	3	3	8	3	0
**Geoalkalibacter subterraneus**	21	12	7	14	20	14	2	2	6	0	3	5	1	5	0	0
**Geobacter sp. M18**	19	16	13	10	17	20	0	1	3	1	5	2	0	3	2	0
**Lysobacter soli**	15	4	10	11	10	11	2	4	15	3	4	6	2	9	6	0
**Acidihalobacter aeolianus**	15	19	12	6	10	9	2	4	5	1	3	0	3	19	4	0
**Acetobacter sp. KACC 21233**	13	17	12	21	18	13	3	0	7	1	1	1	4	1	0	0
**Vibrio azureus**	12	8	11	12	12	44	1	2	3	1	2	1	2	1	0	0
**Pseudomonas simiae**	10	4	8	18	14	3	1	0	6	0	7	2	4	23	12	0
**Massilia sp. Se16.2.3**	10	10	16	12	15	14	0	5	7	1	2	6	2	8	4	0
**Lysobacter gummosus**	7	17	6	10	11	12	0	0	12	5	5	11	6	7	3	0
**Achromobacter sp. B7**	6	13	24	17	10	16	1	1	6	1	3	1	2	4	7	0
**Nitratiruptor labii**	4	4	6	52	4	4	6	6	4	5	5	10	0	1	1	0
**Shewanella sp. SNU WT4**	27	6	21	11	7	15	2	2	9	3	0	1	1	2	4	0
**Bartonella apis**	25	6	1	71	2	2	0	0	0	1	1	0	0	2	0	0
**Mesorhizobium sp. N3**	15	11	9	11	17	6	2	4	10	6	1	1	6	10	1	1
**Cellvibrio sp. KY-YJ-3**	14	13	20	6	14	22	0	4	8	3	5	0	1	0	1	0
**Pseudomonadaceae bacterium SI-3**	10	5	7	10	30	6	0	0	9	1	3	3	2	16	9	0
**Oxalobacter formigenes**	10	17	10	7	15	16	3	5	9	0	5	3	3	4	4	0
**Alcaligenaceae bacterium SJ-26**	9	26	11	12	14	9	4	4	4	2	3	4	2	3	4	0
**Pseudomonas sp. SWI36**	2	1	0	4	3	4	0	0	3	2	4	0	14	48	26	0
**Clostridium cochlearium**	25	8	14	10	22	22	0	0	4	1	1	0	2	0	1	0
**Bradyrhizobium lablabi**	22	8	4	35	4	5	3	0	11	4	2	2	2	7	1	0
**Thermochromatium tepidum**	17	11	12	13	18	10	1	3	5	3	2	3	3	8	1	0
**Flavobacterium crassostreae**	17	15	20	12	8	21	3	3	5	2	0	1	1	1	1	0
**Acidithiobacillus ferrivorans**	16	9	15	12	23	8	3	2	4	0	9	1	1	5	2	0
**Planctomycetes bacterium ETA_A1**	15	8	6	9	15	9	4	7	12	1	8	4	1	7	3	1
**Paraburkholderia hospita**	13	10	11	10	16	8	5	3	9	4	8	6	1	3	3	0
**Paraburkholderia sp. DHF22**	13	6	21	12	18	12	2	2	11	1	1	1	4	5	1	0
**Leuconostoc lactis**	9	1	11	7	12	41	1	2	11	0	8	0	7	0	0	0
**Pseudomonas agarici**	4	9	11	12	9	10	0	0	5	1	5	0	2	23	19	0
**Pseudomonas trivialis**	4	2	11	15	9	8	3	2	5	0	5	3	9	17	17	0
**Clostridium carboxidivorans**	28	22	12	9	13	8	0	0	6	0	4	2	1	2	2	0
**Erwinia pyrifoliae**	20	7	14	13	14	12	3	2	15	1	3	1	0	3	1	0
**Salipiger profundus**	15	6	12	8	11	6	7	6	11	7	2	6	2	6	4	0
**Arenimonas daejeonensis**	12	6	14	10	7	12	5	2	12	1	5	5	3	10	5	0
**Indioceanicola profundi**	10	38	14	10	11	6	2	2	6	2	1	1	3	2	1	0
**Yangia sp. CCB-MM3**	8	11	12	9	15	6	4	2	14	6	3	7	2	5	5	0
**Pseudomonas arsenicoxydans**	7	4	11	5	6	15	2	0	5	1	10	1	12	19	11	0
**Hahella sp. KA22**	17	25	7	5	32	14	0	0	2	0	1	0	0	4	1	0
**Skermanella pratensis**	16	11	9	17	14	8	2	3	8	2	5	1	2	7	3	0

Citrobacter sp. RHBSTW-00678	15	10	20	9	10	6	3	4	12	2	4	2	4	5	2	0
Sphingopyxis sp. 113P3	13	8	2	4	9	17	3	5	19	3	2	2	2	9	10	0
Luteimonas sp. MC1750	12	5	9	5	10	11	5	7	11	6	2	3	1	12	9	0
uncultured Sulfurimonas sp.	12	13	24	24	9	9	3	1	6	0	0	0	3	2	2	0
Desulfuromonas sp. DDH964	9	3	9	11	49	12	1	2	5	0	2	2	0	1	2	0
Alteromonas sp. I4	9	26	17	13	14	12	0	0	11	1	1	1	1	2	0	0
Acinetobacter sp. C16S1	5	9	22	13	9	17	1	2	9	1	0	1	6	8	5	0
Shewanella sp. WE21	5	10	23	13	6	41	1	0	1	4	1	0	0	3	0	0
Pseudomonas savastanoi	4	3	4	18	2	52	0	1	3	2	3	0	1	10	4	1
Methylocystis parvus	42	31	7	2	2	4	1	3	4	3	2	2	2	2	0	0
Pseudopedobacter saltans	17	15	14	10	18	14	1	1	4	0	3	1	3	6	0	0
Geosporobacter ferrireducens	17	10	18	11	25	15	3	3	1	0	2	1	0	1	0	0
Pandoraea fibrosis	16	14	17	5	16	11	5	2	5	4	3	0	1	4	4	0
Rhodoblastus sp.	14	7	10	13	10	7	1	9	15	1	3	1	8	4	4	0
Marinovum algicola	12	12	5	5	17	10	3	6	9	3	4	1	3	8	9	0
Vibrio diabolicus	12	37	5	12	7	15	4	2	3	1	3	1	2	2	1	0
Stella vacuolata	9	7	9	19	11	13	3	5	9	0	5	1	5	8	3	0
Pseudomonas sp. CMR12a	3	6	7	9	7	8	0	0	4	4	5	1	7	29	16	1
Salinivibrio kushneri	32	19	17	3	13	7	2	1	2	0	2	3	1	3	1	0
Lonsdalea britannica	18	15	7	27	16	8	0	0	4	0	2	1	1	2	5	0
Serratia odorifera	16	17	13	12	10	17	0	2	8	2	2	3	2	1	1	0
uncultured bacterium Ad_113_I18_contig2	14	8	11	18	13	19	0	6	9	0	1	2	3	1	1	0
Candidatus Enterovibrio luxaltus	14	4	1	7	73	3	0	1	2	0	1	0	0	0	0	0
Pseudolysobacter antarcticus	12	20	4	35	9	8	1	4	4	3	1	0	2	3	0	0
Burkholderia sp. DHOD12	10	9	22	8	7	11	2	4	13	1	2	4	3	8	2	0
Porphyromonas creviorіcanis	10	17	13	19	10	17	3	0	5	2	2	2	1	3	2	0
Phaeobacter inhibens	8	9	5	18	11	13	2	3	9	4	3	7	1	6	7	0
Ferrimonas sp. S7	8	7	27	26	11	8	3	2	5	2	0	2	1	2	2	0
Pseudomonas lurida	7	2	8	7	9	6	2	0	5	1	5	0	2	22	30	0
Pseudomonas coronafaciens	7	7	6	10	10	18	5	1	13	1	2	1	5	13	7	0
Vibrio alfacsensis	7	8	12	38	22	4	1	0	11	0	0	0	2	1	0	0
Roseburia inulinivorans	5	8	10	11	18	12	2	2	12	3	9	2	4	8	0	0
Nitrosomonas stercoris	60	4	8	6	7	5	0	0	8	0	2	1	0	4	0	0
Proteobacteria bacterium	20	8	13	10	13	19	1	3	2	1	3	4	3	2	3	0
Erysipelotrichaceae bacterium I46	17	11	21	19	4	9	1	0	3	2	1	5	6	4	2	0
Duncaniella sp. B8	15	12	15	13	14	13	1	0	8	3	0	3	5	2	1	0
Rhodobacteraceae bacterium SH-1	10	11	11	7	15	8	3	2	11	6	5	3	2	3	8	0
Nitrospira defluvii	10	16	11	9	19	6	2	1	8	2	6	7	7	1	0	0
Panacibacter ginsenosidivorans	9	10	12	10	10	10	2	3	12	4	4	3	4	9	3	0
Raoultella electrica	8	15	8	14	9	13	2	1	17	0	0	3	0	12	3	0
Halomonas sp. JS92-SW72	6	10	3	11	8	7	0	4	3	4	9	2	5	21	12	0
Pseudomonas asturiensis	6	28	9	13	5	15	1	0	6	0	2	0	4	7	9	0
crAssphage cr7_1	6	5	8	14	9	35	0	1	14	1	7	0	0	4	1	0
Oceanospirillaceae bacterium xL5	5	7	6	19	42	11	2	3	1	0	2	2	2	2	1	0
Pseudomonas sp. DG56-2	4	2	4	7	11	22	1	0	5	2	11	2	8	17	9	0
Algicoccus marinus	20	10	15	16	17	13	1	3	1	0	2	2	3	1	0	0
Rhodospirillaceae bacterium	16	4	13	9	13	16	6	2	6	5	4	4	2	4	0	0
Luteimonas chenhongjianii	14	5	15	11	11	9	1	4	16	4	6	0	1	4	3	0
Burkholderia oklahomensis	13	10	15	10	18	9	5	2	3	0	2	3	5	6	3	0
Pseudoalteromonas sp. 16-SW-7	13	27	2	25	21	0	0	0	13	0	1	0	1	1	0	0
Photorhabdus thracensis	10	20	19	15	10	8	1	1	9	0	2	3	2	4	0	0
Cronobacter malonaticus	9	8	12	12	19	21	1	3	5	1	4	5	1	3	0	0
Sphingomonas melonis	8	11	6	11	7	9	0	0	17	4	5	7	2	9	8	0
Acanthamoeba castellanii	8	1	3	4	4	4	0	4	1	0	0	0	10	45	20	0
Sphinx1.76-related DNA	6	20	6	9	8	3	3	2	6	0	1	12	8	8	12	0
Pseudomonas asplenii	3	6	7	9	8	9	0	1	7	4	10	2	7	17	14	0
Haemophilus haemolyticus	24	12	23	10	9	9	0	3	5	2	0	3	1	2	0	0
Mesorhizobium sp. DCY119	13	8	10	10	9	5	1	3	15	2	7	3	7	8	2	0
Pseudoalteromonas spongiae	11	24	15	8	24	9	1	1	5	1	0	0	2	2	0	0
Phage DP SC_6_H4_2017	10	9	5	24	18	8	0	2	9	4	2	3	0	8	1	0
Clostridium intestinale	10	7	8	4	4	14	1	10	3	4	4	2	1	6	25	0
uncultured bacterium A1Q1_fos_1134	5	12	18	13	20	11	0	4	6	2	2	2	0	6	2	0
Shewanella pealeana	40	8	19	5	6	9	1	1	4	0	3	0	3	3	0	0
Stappia indica	17	8	15	10	12	8	1	3	6	0	3	1	3	5	10	0
Auraticoccus monumenti	7	10	3	9	12	11	4	4	11	3	7	3	3	14	1	0
Methylomonas sp. DH-1	7	7	25	30	8	6	1	2	8	2	1	3	0	0	2	0
Mariniflexile sp. TRM1-10	7	28	10	9	10	19	2	1	3	0	1	3	2	5	2	0
Camellia sinensis	5	7	1	6	3	8	10	9	14	6	10	7	0	9	7	0
Achromobacter sp. LM16	2	3	3	10	4	12	4	0	2	4	16	6	6	20	10	0
Vibrio algivorus	26	7	4	11	12	13	2	2	7	4	3	3	4	2	1	0
Ruminiclostridium cellulolyticum	23	17	11	11	9	7	6	1	4	0	2	5	0	3	2	0
Hypericibacter adhaerens	17	9	8	7	13	15	2	4	7	2	1	3	3	8	2	0
Janibacter indicus	17	9	12	3	12	7	3	4	4	3	3	5	4	12	2	1
Streptococcus sp. oral taxon 431	16	14	20	9	12	11	0	2	0	4	9	0	2	2	0	0
crAssphage cr110_1	16	12	13	17	9	13	0	1	7	4	2	2	0	4	1	0
uncultured microorganism	12	8	9	15	16	13	0	1	14	0	3	1	1	5	3	0
Petrocella atlantisensis	12	12	18	11	16	19	0	1	5	0	2	0	2	2	1	0
Erwinia tracheiphila	11	13	17	9	9	8	0	2	5	4	7	3	3	6	4	0
Pseudoalteromonas piscicida	11	17	15	5	18	13	4	2	9	0	1	2	4	0	0	0
Paraburkholderia sp. 7Q-K02	9	11	17	10	13	16	3	2	8	0	2	1	2	4	3	0
Paraburkholderia caffeinilytica	3	8	16	5	12	12	4	4	11	1	7	5	7	5	1	0
Anaeromyxobacter sp. Fw109-5	23	10	10	13	7	15	2	0	6	1	1	3	1	5	3	0
Citrobacter sp. BDA59-3	16	13	12	27	5	5	0	1	5	1	4	2	2	4	3	0
Lachnospiraceae bacterium oral taxon 500	15	3	9	28	8	11	2	0	11	2	4	1	2	3	1	0
Chromatiaceae bacterium No.7	14	20	14	14	14	8	2	1	3	0	4	1	1	2	2	0
Olsenella timonensis	13	10	10	16	14	14	4	1	7	2	3	1	0	4	1	0
Pantoea sp. 201603H	11	16	7	13	14	14	0	0	10	0	4	3	1	5	2	0
Shewanella aestuarii	9	15	8	11	28	12	0	1	7	1	0	3	2	3	0	0
Methylomonas denitrificans	8	16	10	12	25	10	2	1	2	1	3	3	1	3	3	0

**Eggerthella sp. HF-1101**	7	10	14	10	20	19	4	1	2	0	7	1	3	2	0	0
