# Supplementary material for: Effectiveness of Implementing Hospital Wastewater Treatment Systems as a Measure to Mitigate the Microbial and Antimicrobial Burden on the Environment
Source: Antibiotics (Basel). 2025 Aug 7;14(8):807. doi: 10.3390/antibiotics14080807 (PMC12382850; doi:10.3390/antibiotics14080807)
Supplement: Supplementary file 1 [file antibiotics-14-00807-s001.zip › Table-S5 Detected counts of sequencing reads for each antimicrobial resistance genes (ARGs) by metagenomic DNA-Seq analysis.pdf]

Table S5. Detected counts of sequencing reads for each antimicrobial resistance genes (ARGs) by metagenomic DNA-Seq analysis

Sampling date	2024-0907	2024-0908			2024-0909			2024-0910			2024-0911			2024-0912		
sample	TOHO-20240907-Influent_S1_L001_R1_001	TOHO-20240908-Influent_S2_L001_R1_001	TOHO-20240908-Ozone_S3_L001_R1_001	TOHO-20240908-OzoneUV_S4_L001_R1_001	TOHO-20240909-Influent_S5_L001_R1_001	TOHO-20240909-Ozone_S6_L001_R1_001	TOHO-20240909-OzoneUV_S7_L001_R1_001	TOHO-20240910-Influent_S8_L001_R1_001	TOHO-20240910-Ozone_S9_L001_R1_001	TOHO-20240910-OzoneUV_S10_L001_R1_001	TOHO-20240911-Influent_S11_L001_R1_001	TOHO-20240911-Ozone_S12_L001_R1_001	TOHO-20240911-OzoneUV_S13_L001_R1_001	TOHO-20240912-Influent_S14_L001_R1_001	TOHO-20240912-Ozone_S15_L001_R1_001	TOHO-20240912-OzoneUV_S16_L001_R1_001
nRead	336756	228478	281539	247409	559114	231582	7825	9877	31592	6470	11557	8755	11508	23204	14773	197
n16S	91.60	65.39	76.83	68.52	143.34	66.87	0.64	1.10	6.28	1.12	1.08	1.21	2.06	4.53	4.11	0.00
nCell	20.85	14.84	17.20	15.12	33.08	14.86	0.41	0.59	1.82	0.30	0.61	0.54	0.50	1.21	0.90	0.00
aminoglycoside__AAC(6')-30/AAC(6')-Ib' fusion protein	9.05	0.00	0.00	0.00	0.00	0.00	0.00	0.00	0.00	0.00	0.00	0.00	0.00	0.00	0.00	0.00
aminoglycoside__AAC(6')-31	130.24	215.74	99.21	89.40	98.56	46.94	0.00	0.00	172.03	0.00	470.26	0.00	0.00	234.22	0.00	0.00
aminoglycoside__AAC(6')-IIa	0.00	23.79	19.30	0.00	0.00	23.47	0.00	0.00	0.00	0.00	0.00	0.00	0.00	0.00	0.00	0.00
aminoglycoside__AAC(6')-Ib	0.00	0.00	19.30	0.00	0.00	0.00	0.00	0.00	0.00	0.00	0.00	0.00	0.00	0.00	0.00	0.00
aminoglycoside__AAC(6')-Ib'	0.00	23.79	0.00	0.00	9.72	0.00	0.00	0.00	0.00	0.00	0.00	0.00	0.00	0.00	0.00	0.00
aminoglycoside__AAC(6')-Ib-cr	30.39	0.00	0.00	0.00	8.99	22.49	0.00	0.00	0.00	0.00	0.00	0.00	0.00	0.00	0.00	0.00
aminoglycoside__AAC(6')-Ib11	0.00	0.00	37.59	0.00	0.00	0.00	0.00	0.00	0.00	0.00	0.00	0.00	0.00	0.00	0.00	0.00
aminoglycoside__AAC(6')-Ib9	0.00	0.00	0.00	47.00	8.81	0.00	0.00	0.00	0.00	0.00	0.00	0.00	0.00	0.00	0.00	0.00
aminoglycoside__AAC(6')-Ie-APH(2'')-Ia	12.40	0.00	7.42	0.00	0.00	0.00	0.00	0.00	0.00	0.00	0.00	0.00	0.00	0.00	0.00	0.00
aminoglycoside__AAC(6')-Ii	0.00	0.00	19.52	0.00	0.00	0.00	0.00	0.00	0.00	0.00	0.00	0.00	0.00	0.00	0.00	0.00
aminoglycoside__AAC(6')-Il	19.54	0.00	23.37	0.00	23.53	0.00	0.00	0.00	0.00	0.00	0.00	0.00	0.00	0.00	0.00	0.00
aminoglycoside__ANT(3'')-Ii-AAC(6')-IId fusion protein	6.41	0.00	0.00	0.00	7.73	0.00	0.00	0.00	0.00	0.00	0.00	0.00	0.00	0.00	0.00	0.00
aminoglycoside__ANT(6)-Ib	0.00	0.00	0.00	0.00	0.00	15.15	0.00	0.00	0.00	0.00	0.00	0.00	0.00	0.00	0.00	0.00
aminoglycoside__APH(3'')-Ib	40.46	76.03	75.00	64.73	24.37	69.16	0.00	379.20	312.75	0.00	0.00	427.79	0.00	0.00	0.00	0.00
aminoglycoside__APH(3')-IX	0.00	0.00	13.35	0.00	0.00	0.00	0.00	0.00	0.00	0.00	0.00	0.00	0.00	0.00	0.00	0.00
aminoglycoside__APH(3')-Ia	11.74	0.00	0.00	0.00	0.00	0.00	0.00	0.00	0.00	0.00	0.00	0.00	0.00	0.00	0.00	0.00
aminoglycoside__APH(3')-VI	34.40	16.90	13.71	15.61	13.81	50.02	0.00	0.00	0.00	0.00	0.00	0.00	0.00	0.00	0.00	0.00
aminoglycoside__APH(3')-VIa	0.00	33.80	13.71	15.61	20.72	33.34	0.00	0.00	0.00	0.00	0.00	0.00	0.00	0.00	0.00	0.00
aminoglycoside__APH(6)-Id	64.60	0.00	128.99	44.25	6.43	33.22	0.00	0.00	113.86	0.00	0.00	0.00	0.00	0.00	0.00	0.00
aminoglycoside__aadA	19.03	26.57	13.51	27.43	39.02	51.56	0.00	0.00	0.00	0.00	0.00	0.00	0.00	0.00	0.00	0.00
aminoglycoside__aadA10	0.00	0.00	0.00	0.00	6.46	0.00	0.00	0.00	0.00	0.00	0.00	0.00	0.00	0.00	0.00	0.00
aminoglycoside__aadA12	0.00	0.00	0.00	15.37	0.00	0.00	0.00	0.00	0.00	0.00	0.00	0.00	0.00	0.00	0.00	0.00
aminoglycoside__aadA16	10.57	0.00	0.00	0.00	0.00	15.37	0.00	0.00	0.00	0.00	0.00	0.00	0.00	0.00	0.00	0.00
aminoglycoside__aadA2	11.47	16.90	0.00	0.00	20.72	16.67	0.00	0.00	0.00	0.00	0.00	0.00	0.00	0.00	0.00	0.00
aminoglycoside__aadA22	0.00	0.00	13.51	0.00	0.00	0.00	0.00	0.00	0.00	0.00	0.00	0.00	0.00	0.00	0.00	0.00
aminoglycoside__aadA23	22.93	0.00	0.00	0.00	6.91	16.67	0.00	0.00	0.00	0.00	0.00	0.00	0.00	0.00	0.00	0.00
aminoglycoside__aadA25	11.29	0.00	13.51	0.00	0.00	0.00	0.00	0.00	120.36	0.00	0.00	0.00	0.00	0.00	0.00	0.00
aminoglycoside__aadA3	22.58	49.93	27.01	15.37	6.80	0.00	0.00	0.00	0.00	0.00	0.00	434.30	0.00	0.00	0.00	0.00
aminoglycoside__aadA5	0.00	0.00	13.56	0.00	6.83	0.00	0.00	0.00	0.00	0.00	0.00	0.00	0.00	0.00	0.00	0.00
aminoglycoside__aadA7	0.00	0.00	13.40	0.00	0.00	0.00	0.00	0.00	0.00	0.00	0.00	0.00	0.00	0.00	0.00	0.00
aminoglycoside__aadA8	0.00	16.64	27.01	15.37	6.80	49.26	0.00	0.00	0.00	0.00	0.00	0.00	0.00	0.00	0.00	0.00
aminoglycoside__aadA8b	0.00	0.00	0.00	15.37	13.60	0.00	0.00	0.00	0.00	0.00	0.00	0.00	0.00	0.00	0.00	0.00
aminoglycoside__aadS	72.43	76.25	12.38	14.08	12.46	60.18	0.00	0.00	0.00	0.00	0.00	0.00	0.00	0.00	0.00	0.00
bacitracin__bacA	48.19	0.00	95.96	65.86	45.11	55.57	0.00	0.00	0.00	0.00	0.00	0.00	0.00	0.00	0.00	0.00
beta_lactam__ACI-1	10.46	0.00	0.00	0.00	0.00	0.00	0.00	0.00	0.00	0.00	0.00	0.00	0.00	0.00	0.00	0.00
beta_lactam__AER-1	0.00	0.00	0.00	13.30	0.00	0.00	0.00	0.00	0.00	0.00	0.00	0.00	0.00	0.00	0.00	0.00
beta_lactam__CFE-1	0.00	0.00	0.00	0.00	4.69	0.00	0.00	0.00	0.00	0.00	0.00	0.00	0.00	0.00	0.00	0.00
beta_lactam__CMY-10	0.00	0.00	0.00	0.00	0.00	0.00	0.00	0.00	82.86	0.00	0.00	0.00	0.00	0.00	0.00	0.00
beta_lactam__CMY-112	0.00	0.00	0.00	0.00	4.69	11.33	0.00	0.00	0.00	0.00	0.00	0.00	0.00	0.00	0.00	0.00
beta_lactam__CMY-115	0.00	0.00	0.00	0.00	4.69	0.00	0.00	0.00	0.00	0.00	0.00	0.00	0.00	0.00	0.00	0.00
beta_lactam__CMY-135	0.00	0.00	9.32	0.00	0.00	0.00	0.00	0.00	0.00	0.00	0.00	0.00	0.00	0.00	0.00	0.00
beta_lactam__CMY-19	0.00	11.46	9.30	0.00	0.00	0.00	0.00	0.00	0.00	0.00	0.00	0.00	0.00	0.00	0.00	0.00
beta_lactam__CMY-33	0.00	0.00	0.00	0.00	4.72	0.00	0.00	0.00	0.00	0.00	0.00	0.00	0.00	0.00	0.00	0.00
beta_lactam__CMY-41	0.00	0.00	0.00	0.00	4.69	0.00	0.00	0.00	0.00	0.00	0.00	0.00	0.00	0.00	0.00	0.00
beta_lactam__CMY-48	0.00	0.00	9.32	0.00	4.76	0.00	0.00	0.00	0.00	0.00	0.00	0.00	0.00	0.00	0.00	0.00
beta_lactam__CMY-59	0.00	0.00	0.00	0.00	0.00	0.00	0.00	0.00	0.00	0.00	0.00	279.27	0.00	0.00	0.00	0.00
beta_lactam__CMY-69	0.00	0.00	0.00	0.00	0.00	0.00	0.00	0.00	0.00	0.00	0.00	0.00	0.00	0.00	177.67	0.00
beta_lactam__CMY-8	23.32	0.00	27.89	10.58	14.05	11.30	0.00	0.00	0.00	0.00	0.00	0.00	0.00	0.00	0.00	0.00
beta_lactam__CMY-85	0.00	0.00	0.00	0.00	4.69	0.00	0.00	0.00	0.00	0.00	0.00	0.00	0.00	0.00	0.00	0.00
beta_lactam__CMY-9	0.00	0.00	0.00	0.00	0.00	0.00	0.00	0.00	82.86	0.00	0.00	0.00	0.00	0.00	0.00	0.00
beta_lactam__CTX-M-157	0.00	15.04	0.00	0.00	0.00	0.00	0.00	0.00	0.00	0.00	0.00	0.00	0.00	0.00	0.00	0.00
beta_lactam__CblA-1	0.00	0.00	12.00	0.00	0.00	0.00	0.00	0.00	0.00	0.00	0.00	0.00	0.00	0.00	0.00	0.00
beta_lactam__CcrA beta-lactamase	0.00	0.00	0.00	0.00	7.18	0.00	0.00	0.00	0.00	0.00	0.00	0.00	0.00	0.00	0.00	0.00
beta_lactam__CepS	0.00	11.46	9.30	0.00	0.00	0.00	0.00	0.00	0.00	0.00	0.00	0.00	0.00	0.00	0.00	0.00
beta_lactam__CfxA	0.00	13.63	0.00	0.00	0.00	0.00	0.00	0.00	0.00	0.00	0.00	0.00	0.00	0.00	0.00	0.00
beta_lactam__CfxA2	0.00	13.63	22.13	25.18	16.72	0.00	0.00	0.00	0.00	0.00	0.00	0.00	0.00	0.00	0.00	0.00
beta_lactam__CfxA3	9.25	0.00	0.00	0.00	0.00	0.00	0.00	0.00	0.00	0.00	0.00	0.00	0.00	0.00	0.00	0.00
beta_lactam__CfxA4	0.00	13.63	0.00	0.00	0.00	13.45	0.00	0.00	0.00	0.00	0.00	0.00	0.00	0.00	0.00	0.00
beta_lactam__CfxA5	0.00	0.00	0.00	25.18	0.00	13.45	0.00	0.00	0.00	0.00	0.00	0.00	0.00	0.00	0.00	0.00
beta_lactam__CfxA6	0.00	0.00	21.46	0.00	0.00	26.09	0.00	0.00	0.00	0.00	0.00	0.00	0.00	0.00	0.00	0.00
beta_lactam__GES-11	0.00	0.00	0.00	0.00	6.23	0.00	0.00	0.00	0.00	0.00	0.00	0.00	0.00	0.00	0.00	0.00
beta_lactam__GES-14	113.81	122.00	49.50	211.25	118.41	135.41	0.00	0.00	110.29	0.00	0.00	0.00	0.00	0.00	0.00	0.00
beta_lactam__GES-15	227.63	137.25	123.76	309.83	236.81	225.69	0.00	0.00	220.58	1077.07	0.00	397.98	0.00	0.00	0.00	0.00
beta_lactam__GES-17	0.00	15.25	0.00	0.00	6.23	0.00	0.00	0.00	0.00	0.00	0.00	0.00	0.00	0.00	0.00	0.00
beta_lactam__GES-18	0.00	0.00	0.00	0.00	6.23	15.05	0.00	0.00	0.00	0.00	0.00	0.00	0.00	0.00	0.00	0.00
beta_lactam__GES-19	10.35	0.00	12.38	0.00	0.00	0.00	0.00	0.00	0.00	0.00	0.00	0.00	0.00	0.00	0.00	0.00
beta_lactam__GES-20	41.39	15.25	24.75	0.00	43.62	30.09	0.00	0.00	0.00	0.00	0.00	0.00	0.00	0.00	0.00	0.00
beta_lactam__GES-21	10.35	15.25	0.00	0.00	12.46	0.00	0.00	0.00	0.00	0.00	0.00	0.00	0.00	0.00	0.00	0.00
beta_lactam__GES-24	0.00	0.00	0.00	0.00	24.93	15.05	0.00	0.00	0.00	0.00	0.00	0.00	0.00	0.00	0.00	0.00
beta_lactam__GES-4	10.35	0.00	12.38	0.00	49.85	0.00	0.00	0.00	0.00	0.00	0.00	0.00	0.00	0.00	0.00	0.00
beta_lactam__GES-5	10.35	15.25	12.38	14.08	6.17	29.94	0.00	0.00	0.00	0.00	0.00	0.00	0.00	0.00	0.00	0.00
beta_lactam__GES-6	10.35	15.25	0.00	14.08	0.00	0.00	0.00	0.00	0.00	0.00	0.00	0.00	0.00	0.00	0.00	0.00
beta_lactam__IMP-40	0.00	0.00	0.00	0.00	7.27	0.00	0.00	0.00	0.00	0.00	0.00	0.00	0.00	0.00	0.00	0.00
beta_lactam__Klebsiella pneumoniae OmpK37	7.94	11.70	28.49	0.00	9.56	0.00	0.00	0.00	84.64	0.00	0.00	305.40	0.00	0.00	0.00	0.00
beta_lactam__LCR-1	0.00	16.83	13.66	0.00	6.88	0.00	0.00	0.00	121.74	0.00	0.00	0.00	0.00	0.00	0.00	0.00
beta_lactam__Laribacter hongkongensis ampC beta-lactamase	0.00	0.00	0.00	0.00	4.59	0.00	0.00	0.00	0.00	0.00	0.00	0.00	0.00	0.00	0.00	0.00
beta_lactam__MIR-3	0.00	0.00	0.00	0.00	0.00	0.00	0.00	0.00	0.00	0.00	0.00	0.00	0.00	0.00	0.00	0.00
beta_lactam__MOX-1	7.77	0.00	18.60	10.58	4.68	11.30	0.00	0.00	0.00	0.00	0.00	0.00	0.00	0.00	0.00	0.00
beta_lactam__MOX-3	0.00	0.00	0.00	0.00	4.68	0.00	0.00	0.00	0.00	0.00	0.00	0.00	0.00	0.00	0.00	0.00
beta_lactam__MOX-4	7.77	0.00	9.30	10.58	4.68	0.00	0.00	0.00	0.00	0.00	0.00	0.00	0.00	0.00	0.00	0.00
beta_lactam__MOX-7	0.00	11.43	0.00	0.00	0.00	0.00	0.00	0.00	0.00	0.00	0.00	0.00	0.00	0.00	0.00	0.00
beta_lactam__MYO-1	0.00	0.00	0.00	0.00	6.72	0.00	0.00	0.00	0.00	0.00	0.00	0.00	0.00	0.00	0.00	0.00
beta_lactam__NPS-1	11.42	0.00	0.00	31.09	27.52	16.61	0.00	389.41	0.00	0.00	0.00	0.00	0.00	0.00	0.00	0.00
beta_lactam__OXA-1	136.89	79.76	331.21	136.48	80.72	102.67	0.00	0.00	311.41	1344.56	0.00	0.00	0.00	0.00	0.00	0.00
beta_lactam__OXA-10	11.38	0.00	0.00	0.00	0.00	0.00	0.00	0.00	0.00	0.00	0.00	0.00	0.00	0.00	0.00	0.00
beta_lactam__OXA-119	11.16	0.00	0.00	0.00	26.90	0.00	0.00	0.00	0.00	0.00	0.00	0.00	0.00	0.00	0.00	0.00
beta_lactam__OXA-140	37.91	18.62	0.00	0.00	22.83	0.00	0.00	0.00	0.00	0.00	0.00	0.00	0.00	0.00	0.00	0.00
beta_lactam__OXA-142	0.00	0.00	0.00	0.00	6.72	0.00	0.00	0.00	0.00	0.00	0.00	0.00	0.00	0.00	0.00	0.00
beta_lactam__OXA-147	0.00	0.00	0.00	0.00	6.72	0.00	0.00	0.00	0.00	0.00	0.00	0.00	0.00	0.00	0.00	0.00
beta_lactam__OXA-2	14.01	0.00	12.92	0.00	0.00	0.00	0.00	0.00	0.00	0.00	0.00	0.00	0.00	0.00	0.00	0.00
beta_lactam__OXA-205	0.00	0.00	13.35	0.00	6.72	0.00	0.00	0.00	0.00	0.00	0.00	0.00	0.00	162.02	0.00	0.00
beta_lactam__OXA-209	0.00	0.00	12.96	0.00	0.00	0.00	0.00	0.00	0.00	0.00	0.00	0.00	0.00	0.00	0.00	0.00
beta_lactam__OXA-33	11.60	51.29	0.00	15.79	0.00	0.00	0.00	0.00	0.00	0.00	0.00	0.00	0.00	0.00	0.00	0.00
beta_lactam__OXA-347	32.51	63.89	12.96	0.00	26.11	15.76	0.00	0.00	0.00	0.00	0.00	0.00	0.00	0.00	0.00	0.00
beta_lactam__OXA-37	0.00	0.00	0.00	0.00	6.72	0.00	0.00	0.00	0.00	0.00	0.00	0.00	0.00	0.00	0.00	0.00
beta_lactam__OXA-427	11.25	16.58	13.45	0.00	20.32	16.36	0.00	0.00	0.00	0.00	0.00	0.00	0.00	0.00	0.00	0.00
beta_lactam__OXA-464	0.00	69.20	14.04	47.93	28.28	0.00	0.00	0.00	0.00	0.00	0.00	0.00	0.00	0.00	0.00	0.00
beta_lactam__OXA-58	0.00	0.00	0.00	0.00	0.00	0.00	0.00	361.59	125.61	0.00	0.00	0.00	0.00	0.00	0.00	0.00
beta_lactam__PER-1	0.00	0.00	0.00	0.00	0.00	0.00	0.00	0.00	102.77	0.00	0.00	0.00	0.00	0.00	0.00	0.00
beta_lactam__SHV-6	0.00	0.00	0.00	0.00	6.88	0.00	0.00	0.00	0.00	0.00	0.00	0.00	0.00	0.00	0.00	0.00
beta_lactam__TEM-117	0.00	0.00	13.93	0.00	0.00	0.00	0.00	0.00	0.00	0.00	0.00	0.00	0.00	0.00	0.00	0.00
beta_lactam__VEB-1	19.86	43.99	17.85	0.00	0.00	21.70	0.00	0.00	0.00	0.00	0.00	0.00	0.00	0.00	0.00	0.00
beta_lactam__VEB-9	9.93	14.64	11.88	0.00	5.98	14.44	0.00	0.00	0.00	0.00	0.00	0.00	0.00	0.00	0.00	0.00
chloramphenicol__Campylobacter coli chloramphenicol acetyltrar	0.00	0.00	0.00	0.00	8.60	0.00	0.00	0.00	0.00	0.00	0.00	0.00	0.00	0.00	0.00	0.00
chloramphenicol__catB3	0.00	0.00	0.00	19.25	8.52	0.00	0.00	0.00	0.00	0.00	0.00	0.00	0.00	0.00	322.34	0.00
chloramphenicol__catD	0.00	0.00	0.00	0.00	8.64	0.00	0.00	0.00	0.00	0.00	0.00	0.00	0.00	0.00	0.00	0.00
chloramphenicol__catII from Escherichia coli K-12	0.00	0.00	16.68	0.00	0.00	0.00	0.00	0.00	0.00	0.00	0.00	0.00	0.00	0.00	0.00	0.00
chloramphenicol__cmlA4	0.00	0.00	8.48	0.00	0.00	0.00	0.00	0.00	0.00	0.00	0.00	0.00	0.00	0.00	0.00	0.00
chloramphenicol__cmlA5	7.61	0.00	33.44	9.65	0.00	0.00	0.00	0.00	0.00	0.00	0.00	0.00	0.00	0.00	0.00	0.00
chloramphenicol__cmx	0.00	11.19	0.00	0.00	0.00	0.00	0.00	0.00	0.00	0.00	0.00	0.00	0.00	0.00	0.00	0.00
fosfomycin__FosA	0.00	0.00	0.00	0.00	0.00	0.00	0.00	0.00	0.00	0.00	0.00	0.00	0.00	0.00	0.00	0.00
macrolide-lincosamide-streptogramin__Erm(52)	0.00	0.00	0.00	13.08	0.00	0.00	0.00	0.00	0.00	0.00	0.00	0.00	0.00	0.00	0.00	0.00
macrolide-lincosamide-streptogramin__ere(A)	0.00	23.36	0.00	0.00	0.00	12.41	0.00	0.00	0.00	0.00	0.00	0.00	0.00	0.00	0.00	0.00
macrolide-lincosamide-streptogramin__ere(B)	0.00	0.00	0.00	0.00	0.00	10.31	0.00	0.00	0.00	0.00	0.00	0.00	0.00	0.00	0.00	0.00
macrolide-lincosamide-streptogramin__ere(D)	0.00	0.00	0.00	29.72	26.30	0.00	0.00	0.00	0.00	0.00	0.00	0.00	0.00	0.00	0.00	0.00
macrolide-lincosamide-streptogramin__erm(B)	12.12	0.00	14.50	0.00	0.00	0.00	0.00	0.00	0.00	0.00	0.00	0.00	0.00	0.00	0.00	0.00
macrolide-lincosamide-streptogramin__erm(F)	0.00	16.45	106.82	30.39	40.34	32.47	0.00	0.00	0.00	0.00	0.00	0.00	0.00	0.00	254.48	0.00
macrolide-lincosamide-streptogramin__erm(G)	12.17	0.00	0.00	33.13	14.66	0.00	0.00	0.00	0.00	0.00	0.00	0.00	0.00	0.00	0.00	0.00
macrolide-lincosamide-streptogramin__lnu(B)	0.00	0.00	0.00	0.00	0.00	32.35	0.00	0.00	0.00	0.00	0.00	0.00	0.00	0.00	0.00	0.00
macrolide-lincosamide-streptogramin__lnu(C)	36.21	0.00	0.00	0.00	0.00	0.00	0.00	0.00	0.00	0.00	0.00	0.00	0.00	0.00	0.00	0.00
macrolide-lincosamide-streptogramin__lsa(E)	11.12	0.00	0.00	0.00	6.70	0.00	0.00	0.00	0.00	0.00	0.00	0.00	0.00	0.00	0.00	0.00
macrolide-lincosamide-streptogramin__macA	0.00	3.93	5.85	0.00	6.67	3.88	0.00	0.00	0.00	0.00	0.00	0.00	0.00	0.00	0.00	0.00
macrolide-lincosamide-streptogramin__macB	4.58	6.75	0.00	4.19	5.52	4.45	0.00	0.00	0.00	79.51	0.00	0.00	0.00	0.00	0.00	0.00
macrolide-lincosamide-streptogramin__mef(B)	21.78	32.10	26.05	19.76	21.86	52.79	0.00	0.00	0.00	0.00	0.00	0.00	0.00	0.00	0.00	0.00
macrolide-lincosamide-streptogramin__mef(En2)	7.41	0.00	0.00	0.00	4.46	0.00	0.00	0.00	0.00	0.00	0.00	0.00	0.00	0.00	0.00	0.00
macrolide-lincosamide-streptogramin__mel	0.00	10.81	26.60	0.00	13.23	10.66	0.00	0.00	0.00	0.00	0.00	0.00	0.00	0.00	0.00	0.00
macrolide-lincosamide-streptogramin__mph(A)	9.70	0.00	0.00	0.00	5.94	0.00	0.00	0.00	0.00	0.00	0.00	0.00	0.00	0.00	0.00	0.00
macrolide-lincosamide-streptogramin__mph(B)	0.00	0.00	0.00	25.58	0.00	0.00	0.00	0.00	0.00	0.00	0.00	0.00	0.00	0.00	0.00	0.00
macrolide-lincosamide-streptogramin__mph(G)	0.00	0.00	12.08	0.00	0.00	0.00	0.00	0.00	0.00	0.00	0.00	0.00	0.00	0.00	0.00	0.00
macrolide-lincosamide-streptogramin__vat(B)	14.01	0.00	0.00	19.07	8.44	20.37	0.00	0.00	149.31	0.00	0.00	0.00	0.00	0.00	0.00	0.00
multidrug__Escherichia coli mdfA	14.31	10.94	0.00	19.76	26.83	32.39	0.00	0.00	0.00	0.00	0.00	0.00	0.00	0.00	0.00	0.00
multidrug__Klebsiella pneumoniae acrA	0.00	0.00	0.00	0.00	1.65	3.45	0.00	0.00	0.00	0.00	0.00	0.00	80.24	0.00	0.00	0.00
multidrug__MexA	0.00	0.00	0.00	0.00	0.00	0.00	0.00	0.00	0.00	0.00	0.00	0.00	0.00	0.00	0.00	0.00
multidrug__MexB	2.93	7.02	6.90	6.49	1.72	9.66	0.00	0.00	0.00	0.00	0.00	0.00	27.64	13.69	0.00	0.00
multidrug__MexC	2.57	0.00	0.00	3.50	1.55	11.22	0.00	0.00	0.00	0.00	0.00	0.00	0.00	0.00	0.00	0.00
multidrug__MexD	0.00	0.00	2.27	1.29	1.14	1.38	0.00	0.00	0.00	0.00	0.00	0.00	0.00	27.57	0.00	0.00
multidrug__MexE	4.99	0.00	0.00	3.39	11.90	3.63	0.00	0.00	0.00	0.00	0.00	0.00	0.00	0.00	0.00	0.00
multidrug__MexF	0.93	1.37	0.00	2.54	0.56	0.00	0.00	0.00	0.00	0.00	0.00	0.00	27.35	0.00	0.00	0.00
multidrug__MuxA	0.00	0.00	0.00	0.00	0.00	0.00	0.00	0.00	0.00	0.00	0.00	0.00	0.00	33.72	0.00	0.00
multidrug__MuxB	0.00	1.40	0.00	0.00	1.71	0.00	0.00	0.00	0.00	0.00	0.00	0.00	0.00	0.00	0.00	0.00

multidrug__MuxC	0.00	1.41	0.00	0.00	0.00	0.00	0.00	0.00	0.00	0.00	0.00	0.00	0.00	0.00	0.00	0.00
multidrug__OpmH	0.00	0.00	0.00	0.00	0.00	0.00	0.00	0.00	0.00	0.00	0.00	0.00	0.00	0.00	0.00	0.00
multidrug__OprJ	0.00	0.00	0.00	0.00	1.24	0.00	0.00	0.00	0.00	0.00	0.00	0.00	0.00	0.00	0.00	0.00
multidrug__OprM	0.00	3.12	0.00	0.00	1.27	6.15	0.00	0.00	0.00	0.00	0.00	0.00	0.00	0.00	0.00	0.00
multidrug__OprN	0.00	0.00	0.00	2.85	0.00	0.00	0.00	0.00	0.00	0.00	0.00	0.00	0.00	0.00	0.00	0.00
multidrug__PmpM	0.00	0.00	0.00	0.00	0.00	0.00	0.00	0.00	0.00	0.00	0.00	0.00	0.00	0.00	0.00	0.00
multidrug__RanA	0.00	16.96	0.00	15.67	0.00	0.00	0.00	0.00	0.00	0.00	0.00	0.00	0.00	0.00	0.00	0.00
multidrug__RanB	0.00	0.00	28.08	15.98	7.07	0.00	0.00	0.00	0.00	0.00	0.00	0.00	0.00	0.00	0.00	0.00
multidrug__abeM	0.00	9.77	7.93	9.02	3.99	0.00	0.00	0.00	0.00	0.00	0.00	0.00	0.00	0.00	0.00	0.00
multidrug__acrE	0.00	0.00	0.00	0.00	3.10	0.00	0.00	0.00	0.00	0.00	0.00	98.89	0.00	0.00	0.00	0.00
multidrug__acrF	7.64	8.45	6.86	6.50	10.36	11.11	41.08	0.00	0.00	0.00	0.00	0.00	55.89	13.85	21.76	0.00
multidrug__adeF	0.93	1.38	0.00	0.00	0.56	0.00	0.00	0.00	0.00	0.00	0.00	0.00	0.00	0.00	0.00	0.00
multidrug__adeH	0.00	0.00	0.00	0.00	1.23	0.00	0.00	0.00	0.00	0.00	0.00	0.00	0.00	0.00	0.00	0.00
multidrug__adeI	0.00	3.88	5.99	0.00	0.00	0.00	0.00	0.00	0.00	0.00	0.00	0.00	0.00	0.00	0.00	0.00
multidrug__amrB	0.00	1.40	0.00	0.00	0.00	1.38	0.00	0.00	0.00	0.00	0.00	0.00	0.00	0.00	0.00	0.00
multidrug__ceoB	0.00	0.00	0.00	0.00	0.00	0.00	0.00	0.00	0.00	0.00	0.00	0.00	0.00	0.00	0.00	0.00
multidrug__emrA	2.54	11.22	3.04	6.91	4.59	7.38	0.00	0.00	0.00	0.00	0.00	0.00	0.00	0.00	0.00	0.00
multidrug__emrB	3.89	8.63	4.65	2.63	4.67	0.00	0.00	0.00	20.61	0.00	0.00	0.00	0.00	0.00	44.24	0.00
multidrug__emrD	15.29	0.00	0.00	31.04	41.04	21.92	0.00	0.00	0.00	0.00	0.00	0.00	0.00	0.00	0.00	0.00
multidrug__emrK	0.00	0.00	0.00	0.00	1.70	0.00	0.00	0.00	0.00	0.00	0.00	0.00	0.00	0.00	0.00	0.00
multidrug__emrY	0.00	0.00	0.00	0.00	0.00	2.81	0.00	0.00	0.00	0.00	0.00	0.00	0.00	28.06	0.00	0.00
multidrug__mdtE	0.00	3.19	5.80	3.85	10.24	3.74	0.00	0.00	0.00	0.00	0.00	0.00	0.00	0.00	0.00	0.00
multidrug__mdtF	4.77	0.00	2.28	2.60	4.59	1.39	0.00	0.00	0.00	0.00	0.00	0.00	0.00	0.00	0.00	0.00
multidrug__mdtH	21.27	21.78	8.84	0.00	4.45	10.74	0.00	0.00	0.00	0.00	0.00	284.13	0.00	0.00	0.00	0.00
multidrug__mdtK	38.75	9.58	0.00	8.53	15.23	9.11	279.64	0.00	0.00	0.00	0.00	0.00	0.00	0.00	0.00	0.00
multidrug__mdtL	7.56	0.00	27.16	0.00	45.57	10.99	0.00	0.00	0.00	0.00	0.00	0.00	221.11	0.00	0.00	0.00
multidrug__mdtM	7.19	21.20	0.00	0.00	8.66	31.37	0.00	0.00	0.00	0.00	0.00	0.00	0.00	0.00	0.00	0.00
multidrug__mdtN	0.00	4.25	0.00	0.00	5.40	4.42	0.00	0.00	0.00	0.00	0.00	0.00	0.00	0.00	0.00	0.00
multidrug__mdtO	1.45	0.00	1.73	1.97	1.75	6.32	0.00	0.00	0.00	0.00	0.00	0.00	0.00	0.00	0.00	0.00
multidrug__mdtP	6.11	6.04	0.00	2.76	3.67	11.80	0.00	0.00	0.00	0.00	59.47	0.00	0.00	0.00	0.00	0.00
multidrug__mexK	0.00	2.85	1.16	1.31	0.00	0.00	0.00	0.00	10.29	0.00	0.00	0.00	0.00	0.00	0.00	0.00
multidrug__mexN	0.00	0.00	0.00	0.00	0.00	0.00	0.00	0.00	0.00	0.00	0.00	0.00	0.00	0.00	0.00	0.00
multidrug__mexW	0.00	0.00	0.00	2.65	0.59	0.00	0.00	0.00	0.00	0.00	0.00	0.00	0.00	14.17	22.25	0.00
multidrug__msbA	5.10	0.00	0.00	0.00	12.29	0.00	0.00	0.00	0.00	0.00	0.00	0.00	0.00	0.00	0.00	0.00
multidrug__mtrD	0.00	1.37	0.00	0.00	0.00	0.00	0.00	0.00	0.00	0.00	0.00	0.00	0.00	0.00	0.00	0.00
multidrug__oqxB	0.00	1.39	0.00	0.00	0.00	0.00	0.00	0.00	0.00	0.00	0.00	0.00	27.59	0.00	0.00	0.00
multidrug__qacE	0.00	0.00	0.00	0.00	16.26	0.00	0.00	0.00	0.00	0.00	0.00	0.00	0.00	0.00	0.00	0.00
multidrug__qacEdelta1	180.75	76.12	61.77	70.29	186.63	262.84	0.00	0.00	275.25	0.00	0.00	2979.66	0.00	0.00	0.00	0.00
multidrug__qacH	0.00	0.00	0.00	36.74	65.04	0.00	0.00	0.00	0.00	0.00	0.00	0.00	0.00	0.00	615.37	0.00
multidrug__sdeY	3.77	2.78	1.13	3.85	1.13	1.37	0.00	0.00	0.00	0.00	0.00	0.00	0.00	0.00	0.00	0.00
multidrug__smeE	2.84	2.78	2.26	0.00	1.71	2.75	0.00	0.00	0.00	0.00	0.00	0.00	0.00	0.00	0.00	0.00
multidrug__tolC	6.06	5.95	4.36	0.00	9.67	2.94	0.00	0.00	21.40	0.00	0.00	0.00	0.00	29.32	0.00	0.00
mupirocin__Bifidobacteria intrinsic ileS conferring resistance to r	0.00	0.00	0.00	0.00	0.00	7.80	0.00	0.00	0.00	0.00	0.00	0.00	0.00	0.00	0.00	0.00
other_peptide_antibiotics__microcin efflux pumu gene yojI	10.86	0.00	12.99	0.00	6.54	23.68	0.00	0.00	0.00	0.00	0.00	0.00	0.00	0.00	0.00	0.00
polymyxin__arnA	27.00	6.63	5.38	12.25	40.64	19.63	0.00	0.00	0.00	0.00	0.00	0.00	0.00	0.00	102.56	0.00
polymyxin__eptA	10.86	0.00	12.99	0.00	13.08	15.79	0.00	0.00	57.87	0.00	0.00	0.00	0.00	0.00	0.00	0.00
polymyxin__pmrF	0.00	13.59	0.00	0.00	22.22	26.82	0.00	0.00	0.00	0.00	0.00	0.00	0.00	0.00	0.00	0.00
polymyxin__rosA	11.01	10.81	0.00	5.00	4.42	5.32	0.00	0.00	0.00	0.00	0.00	0.00	0.00	0.00	0.00	0.00
polymyxin__rosB	5.27	7.78	3.15	3.59	9.59	7.81	0.00	0.00	0.00	0.00	0.00	0.00	0.00	38.27	0.00	0.00
polymyxin__ugd	45.92	11.28	45.77	10.42	23.05	22.26	0.00	0.00	0.00	0.00	0.00	0.00	0.00	0.00	0.00	0.00
quinolone__QnrS2	0.00	0.00	16.29	0.00	0.00	0.00	0.00	0.00	0.00	0.00	0.00	0.00	0.00	0.00	0.00	0.00
quinolone__QnrS6	0.00	0.00	0.00	0.00	8.20	0.00	0.00	0.00	0.00	0.00	0.00	0.00	0.00	0.00	0.00	0.00
quinolone__QnrVC1	0.00	0.00	16.29	0.00	0.00	0.00	0.00	0.00	0.00	0.00	0.00	0.00	0.00	0.00	0.00	0.00
sulfonamide__sul1	314.98	395.64	339.53	195.96	333.31	479.86	1754.50	0.00	802.78	553.98	280.93	943.97	0.00	470.99	0.00	0.00
sulfonamide__sul2	42.44	32.24	13.11	0.00	52.21	15.88	0.00	0.00	0.00	0.00	0.00	0.00	320.65	0.00	0.00	0.00
tetracycline__tet(32)	4.65	13.70	5.56	0.00	13.99	0.00	0.00	0.00	0.00	0.00	0.00	0.00	0.00	0.00	0.00	0.00
tetracycline__tet(36)	69.60	102.58	49.95	31.58	53.10	67.47	0.00	158.20	98.92	0.00	0.00	0.00	0.00	0.00	0.00	0.00
tetracycline__tet(37)	0.00	40.53	0.00	0.00	0.00	0.00	0.00	0.00	0.00	0.00	0.00	0.00	0.00	0.00	0.00	0.00
tetracycline__tet(39)	0.00	33.24	35.97	10.23	0.00	21.86	0.00	256.32	0.00	0.00	0.00	0.00	0.00	0.00	0.00	0.00
tetracycline__tet(40)	0.00	0.00	0.00	9.96	4.41	0.00	0.00	0.00	0.00	0.00	0.00	0.00	0.00	0.00	0.00	0.00
tetracycline__tet(44)	4.64	6.84	0.00	0.00	2.79	0.00	0.00	0.00	0.00	0.00	0.00	0.00	0.00	0.00	0.00	0.00
tetracycline__tet(A)	0.00	0.00	0.00	11.29	5.00	10.04	0.00	0.00	0.00	0.00	0.00	0.00	0.00	0.00	0.00	0.00
tetracycline__tet(C)	46.78	11.19	18.17	20.67	23.12	10.90	0.00	538.54	0.00	0.00	0.00	0.00	0.00	0.00	0.00	0.00
tetracycline__tet(E)	0.00	0.00	8.77	10.16	4.49	0.00	0.00	0.00	0.00	0.00	0.00	0.00	0.00	0.00	0.00	0.00
tetracycline__tet(G)	0.00	0.00	0.00	10.34	4.57	0.00	0.00	0.00	80.96	0.00	0.00	0.00	0.00	0.00	0.00	0.00
tetracycline__tet(M)	13.94	6.85	5.56	0.00	2.80	6.76	0.00	0.00	0.00	0.00	0.00	0.00	0.00	0.00	0.00	0.00
tetracycline__tet(O)	9.29	0.00	11.12	6.33	2.80	0.00	0.00	0.00	0.00	0.00	0.00	0.00	0.00	0.00	0.00	0.00
tetracycline__tet(Q)	59.77	33.97	49.87	81.55	41.51	40.42	0.00	157.95	49.38	0.00	0.00	0.00	0.00	0.00	0.00	0.00
tetracycline__tet(W)	4.65	13.70	5.56	0.00	2.80	13.52	0.00	0.00	0.00	0.00	0.00	178.75	0.00	0.00	0.00	0.00
tetracycline__tet(X)	0.00	0.00	0.00	0.00	0.00	11.19	0.00	0.00	0.00	0.00	0.00	0.00	0.00	0.00	0.00	0.00
tetracycline__tet(Y)	8.05	0.00	0.00	0.00	0.00	0.00	0.00	0.00	0.00	0.00	0.00	0.00	0.00	0.00	0.00	0.00
tetracycline__tetA(46)	0.00	0.00	0.00	0.00	1.56	0.00	0.00	0.00	0.00	0.00	0.00	0.00	0.00	0.00	0.00	0.00
tetracycline__tetB(46)	2.57	0.00	0.00	0.00	0.00	0.00	0.00	0.00	0.00	0.00	0.00	0.00	0.00	0.00	0.00	0.00
tetracycline__tetX2	9.34	25.34	11.17	0.00	0.00	0.00	0.00	0.00	0.00	0.00	0.00	0.00	0.00	0.00	0.00	0.00
trimethoprim__dfrA1	0.00	0.00	0.00	51.49	0.00	0.00	0.00	0.00	0.00	0.00	0.00	0.00	0.00	0.00	0.00	0.00
vancomycin__vanB	22.12	0.00	10.39	0.00	16.19	0.00	0.00	0.00	0.00	0.00	0.00	0.00	0.00	0.00	0.00	0.00
vancomycin__vanG	0.00	0.00	10.18	0.00	0.00	0.00	0.00	0.00	0.00	0.00	0.00	0.00	0.00	0.00	0.00	0.00
vancomycin__vanHB	9.19	0.00	0.00	0.00	16.61	13.37	0.00	0.00	0.00	0.00	0.00	0.00	0.00	0.00	0.00	0.00
vancomycin__vanO	0.00	0.00	0.00	11.68	0.00	0.00	0.00	0.00	0.00	0.00	0.00	0.00	0.00	0.00	0.00	0.00
vancomycin__vanXB	0.00	0.00	0.00	0.00	17.71	64.13	0.00	0.00	0.00	0.00	0.00	0.00	0.00	0.00	0.00	0.00
vancomycin__vanYB	0.00	21.88	0.00	0.00	0.00	0.00	0.00	0.00	0.00	0.00	0.00	0.00	0.00	0.00	0.00	0.00
vancomycin__vanYD	0.00	0.00	0.00	0.00	5.04	0.00	0.00	0.00	0.00	0.00	0.00	0.00	0.00	0.00	0.00	0.00
